# Supplementary material for: Characterization of structural changes in modern and archaeological burnt bone: Implications for differential preservation bias
Source: PLoS One. 2021 Jul 28;16(7):e0254529. doi: 10.1371/journal.pone.0254529 (PMC8318310; doi:10.1371/journal.pone.0254529)
Supplement: S1 Appendix — (PDF) [file pone.0254529.s001.pdf]

S1 Appendix: SE microscopy imaging instrument and magnification details

Secondary electron (SE) images were acquired for two samples of archaeological bone and three samples of modern bone annealed at different temperatures (300˚, 700˚, and 1200˚C) in a Quattro environmental scanning electron microscope (eSEM), manufactured by ThermoFischer Scientific. The SE images were obtained at an accelerating voltage of either 10kV or 20kV and with an electron beam width, or size spot, of 3.0. Spot 3 is commonly used to attain sufficient signal without compromising resolution. To prevent the buildup of charge on sample surfaces, the SE images were acquired in low vacuum mode with partial pressure of water set at 400 Pa. A low vacuum detector (LVD), which is optimized for this pressure range, was used to measure the SE image signal.
