# Supplementary material for: Characterization of structural changes in modern and archaeological burnt bone: Implications for differential preservation bias
Source: PLoS One. 2021 Jul 28;16(7):e0254529. doi: 10.1371/journal.pone.0254529 (PMC8318310; doi:10.1371/journal.pone.0254529)
Supplement: S1 Table — (PDF) [file pone.0254529.s002.pdf]

S1 Table: Experimental modern bone FTIR-ATR relevant peak height values.

Sample	Species	Temperature (˚C)	1650 cm^{-1}	1415 cm^{-1}	1035 cm^{-1}	874 cm^{-1}	625 cm^{-1}	605 cm^{-1}	595 cm^{-1}	565 cm^{-1}
UCDX4_1505_unburnt_1.1	Cow	25	0.027	0.031	0.11	0.041	0.039	0.083	0.077	0.117
UCDX4_1505_unburnt_1.2		25	0.0211	0.025	0.0885	0.0323	0.0338	0.071	0.0626	0.0969
UCDX4_1500_unburnt_1.1	Cow	25	0.037	0.042	0.128	0.05	0.052	0.01	0.091	0.139
UCDX4_1500_unburnt_1.2		25	0.037	0.037	0.115	0.044	0.049	0.091	0.082	0.122
UCDX4_1501_unburnt_1.1	Cow	25	0.005	0.018	0.137	0.026	0.031	0.081	0.07	0.13
UCDX4_1501_unburnt_1.2		25	0.004	0.019	0.132	0.026	0.032	0.077	0.068	0.126
UCDX4_2120_unburnt_1.1	Cow	25	0.042	0.05	0.132	0.052	0.053	0.105	0.098	0.14
UCDX4_2120_unburnt_1.2		25	0.041	0.049	0.132	0.052	0.059	0.099	0.093	0.129
UCDX4_4101_unburnt_1.1	Horse	25	0.041	0.05	0.141	0.052	0.057	0.108	0.099	0.144
UCDX4_4101_unburnt_1.2		25	0.041	0.051	0.136	0.051	0.053	0.104	0.095	0.141
UCDX4_1505_100_30_1.1	Cow	100	0.0088	0.012	0.0403	0.0213	0.0177	0.0377	0.0352	0.0531
UCDX4_1505_100_30_1.2		100	0.0134	0.0165	0.0449	0.0247	0.0216	0.0413	0.038	0.0571
UCDX4_H1_100_30_1.1	Horse	100	0.0115	0.0147	0.033	0.0187	0.0174	0.0291	0.0273	0.0377
UCDX4_H1_100_30_1.2		100	0.02	0.0154	0.0314	0.0194	0.017	0.0275	0.0262	0.0359
UCDX4_1505_200_30_1.1	Cow	200	0.007	0.0097	0.0309	0.0164	0.0134	0.0273	0.0259	0.0414
UCDX4_1505_200_30_1.2		200	0.0019	0.0044	0.0271	0.0106	0.0079	0.024	0.0217	0.0368
UCDX4_H1_200_30_1.1	Horse	200	0.0164	0.0209	0.0563	0.0269	0.025	0.0483	0.0447	0.0652
UCDX4_H1_200_30_1.2		200	0.0151	0.0189	0.0487	0.0238	0.0248	0.044	0.0409	0.0597
UCDX4_H2_200_30_1.1	Horse	200	0.0223	0.0277	0.0759	0.0325	0.03	0.0633	0.0577	0.0886
UCDX4_H2_200_30_1.2		200	0.0219	0.0271	0.0717	0.0308	0.0332	0.0586	0.0524	0.0802
UCDX4_1505_300_30_1.1	Cow	300	0.014	0.027	0.106	0.034	0.034	0.074	0.064	0.106
UCDX4_1505_300_30_1.2		300	0.0001	0.0055	0.0421	0.0084	0.0108	0.0312	0.0251	0.0469
UCDX4_1500_300_30_1.1	Cow	300	0.017	0.032	0.154	0.035	0.049	0.103	0.088	0.148
UCDX4_1500_300_30_1.2		300	0.019	0.037	0.184	0.043	0.057	0.12	0.101	0.18
UCDX4_H1_300_30_1.1	Horse	300	0.016	0.031	0.113	0.031	0.032	0.08	0.069	0.111
UCDX4_H1_300_30_1.2		300	0.022	0.042	0.151	0.038	0.053	0.101	0.087	0.135
UCDX4_H2_300_30_1.1	Horse	300	0.025	0.042	0.151	0.041	0.047	0.1	0.087	0.14
UCDX4_H2_300_30_1.2		300	0.019	0.033	0.121	0.032	0.035	0.081	0.07	0.113
UCDX4_H2T2_300_30_1.1	Horse	300	0.024	0.041	0.14	0.037	0.038	0.096	0.08	0.139
UCDX4_H2T2_300_30_1.2		300	0.027	0.047	0.17	0.042	0.04	0.109	0.091	0.154

UCDX4_1505_300_30x30_1.1	Cow	300	0.009	0.023	0.111	0.032	0.031	0.078	0.068	0.122
UCDX4_1505_300_30x30_1.2		300	0.009	0.021	0.101	0.03	0.03	0.069	0.061	0.103
UCDX4_H1_300_30x30_1.1	Horse	300	0.015	0.032	0.152	0.033	0.039	0.103	0.085	0.148
UCDX4_H1_300_30x30_1.2		300	0.017	0.037	0.157	0.036	0.048	0.0106	0.089	0.15
UCDX4_H1T2_300_30x30_1.1	Horse	300	0.02	0.041	0.159	0.042	0.046	0.106	0.091	0.156
UCDX4_H1T2_300_30x30_1.2		300	0.022	0.046	0.184	0.046	0.048	0.121	0.102	0.133
UCDX4_H1_300_50_1.1	Horse	300	0.0125	0.026	0.0971	0.0299	0.0373	0.0639	0.0605	0.0988
UCDX4_H1_300_50_1.2		300	0.021	0.041	0.149	0.042	0.054	0.093	0.084	0.136
UCDX4_H2_300_50_1.1	Horse	300	0.025	0.053	0.223	0.045	0.059	0.129	0.114	0.205
UCDX4_H2_300_50_1.2		300	0.024	0.05	0.221	0.042	0.064	0.134	0.112	0.203
UCDX4_1505_400_30_1.1	Cow	400	0.004	0.019	0.12	0.027	0.03	0.076	0.067	0.123
UCDX4_1505_400_30_1.2		400	0.008	0.022	0.114	0.03	0.033	0.079	0.066	0.119
UCDX4_1500_400_30_1.1	Cow	400	0.019	0.039	0.213	0.014	0.052	0.13	0.11	0.191
UCDX4_1500_400_30_1.2		400	0.016	0.036	0.204	0.014	0.062	0.126	0.106	0.194
UCDX4_1501_400_30_1.1	Cow	400	0.011	0.024	0.114	0.032	0.035	0.075	0.069	0.12
UCDX4_1501_400_30_1.2		400	0.012	0.026	0.117	0.033	0.038	0.076	0.071	0.119
UCDX4_H1_400_30_1.1	Horse	400	0.011	0.034	0.161	0.035	0.038	0.098	0.084	0.152
UCDX4_H1_400_30_1.2		400	0.011	0.035	0.159	0.035	0.036	0.095	0.085	0.153
UCDX4_H2_400_30_1.1	Horse	400	0.02	0.049	0.214	0.043	0.054	0.125	0.102	0.192
UCDX4_H2_400_30_1.2		400	0.018	0.039	0.15	0.036	0.04	0.096	0.143	0.08
UCDX4_1505_500_30_1.1	Cow	500	0.0066	0.018	0.114	0.023	0.028	0.07	0.056	0.106
UCDX4_1505_500_30_1.2		500	0.006	0.016	0.101	0.03	0.033	0.075	0.063	0.118
UCDX4_1500_500_30_1.1	Cow	500	0.015	0.033	0.192	0.042	0.047	0.119	0.098	0.183
UCDX4_1500_500_30_1.2		500	0.011	0.029	0.166	0.036	0.052	0.11	0.088	0.161
UCDX4_H1_500_30_1.1	Horse	500	0.0104	0.0232	0.09	0.0252	0.0319	0.0648	0.0537	0.0929
UCDX4_H1_500_30_1.2		500	0.0107	0.024	0.0926	0.0277	0.0298	0.0639	0.0537	0.0904
UCDX4_H2_500_30_1.1	Horse	500	0.009	0.035	0.172	0.033	0.043	0.108	0.088	0.158
UCDX4_H2_500_30_1.2		500	0.009	0.034	0.158	0.032	0.035	0.102	0.083	0.149
UCDX4_1505_600_30_1.1	Cow	600	-0.0022	0.0045	0.0767	0.0102	0.0163	0.0539	0.0381	0.0811
UCDX4_1505_600_30_1.2		600	0	0.009	0.103	0.016	0.022	0.069	0.051	0.107
UCDX4_1500_600_30_1.1	Cow	600	0.009	0.021	0.185	0.023	0.048	0.106	0.78	0.165

UCDX4_1500_600_30_1.2	Cow	600	0.009	0.021	0.185	0.023	0.056	0.112	0.77	0.165
UCDX4_1501_600_30_1.1	Cow	600	0.009	0.021	0.145	0.029	0.037	0.09	0.072	0.142
UCDX4_1501_600_30_1.2		600	0.0034	0.0139	0.0995	0.0182	0.0263	0.0617	0.0482	0.095
UCDX4_H1_600_30_1.1	Horse	600	0.013	0.028	0.122	0.032	0.04	0.085	0.065	0.119
UCDX4_H1_600_30_1.2		600	0.012	0.025	0.108	0.028	0.033	0.077	0.058	0.108
UCDX4_H2_600_30_1.1	Horse	600	0.006	0.032	0.193	0.034	0.04	0.112	0.083	0.176
UCDX4_H2_600_30_1.2		600	0.008	0.036	0.193	0.036	0.043	0.116	0.088	0.18
UCDX4_H4_600_30_1.1	Horse	600	0.01	0.025	0.143	0.027	0.031	0.085	0.061	0.125
UCDX4_H4_600_30_1.2		600	0.011	0.03	0.152	0.03	0.036	0.089	0.68	0.133
UCDX4_1505_700_30_1.1	Cow	700	0	0.005	0.098	0.013	0.021	0.061	0.044	0.097
UCDX4_1505_700_30_1.2		700	0.0018	0.0057	0.0705	0.0112	0.0177	0.051	0.0335	0.0726
UCDX4_1500_700_30_1.1	Cow	700	0.011	0.026	0.318	0.031	0.061	0.166	0.108	0.242
UCDX4_1500_700_30_1.2		700	0.012	0.025	0.0292	0.03	0.056	0.154	0.108	0.242
UCDX4_1501_700_30_1.1	Cow	700	0.005	0.013	0.164	0.02	0.035	0.095	0.071	0.148
UCDX4_1501_700_30_1.2		700	0.004	0.012	0.153	0.019	0.034	0.091	0.068	0.139
UCDX4_H1_700_30_1.1	Horse	700	0.007	0.046	0.423	0.042	0.058	0.242	0.136	0.329
UCDX4_H1T1_700_30_1.1	Horse	700	0.009	0.049	0.0468	0.047	0.062	0.236	0.139	0.344
UCDX4_H1T1_700_30_1.2		700	0.01	0.048	0.0384	0.04	0.061	0.22	0.128	0.307
UCDX4_H2_700_30_1.1	Horse	700	0.013	0.048	0.34	0.042	0.048	0.177	0.121	0.269
UCDX4_H2_700_30_1.2		700	0.014	0.051	0.298	0.042	0.047	0.158	0.112	0.237
UCDX4_1505_700_30x30_1.1	Cow	700	0.004	0.01	0.153	0.02	0.037	0.099	0.063	0.145
UCDX4_1505_700_30x30_1.2		700	0.004	0.01	0.126	0.018	0.032	0.082	0.054	0.121
UCDX4_1501_700_30x30_1.1	Cow	700	0.006	0.016	0.233	0.023	0.052	0.142	0.084	0.142
UCDX4_1501_700_30x30_1.2		700	0.006	0.017	0.214	0.022	0.05	0.134	0.08	0.134
UCDX4_H0_700_30x30_1.1	Horse	700	0.011	0.044	0.4	0.038	0.062	0.213	0.124	0.319
UCDX4_H0_700_30x30_1.2		700	0.012	0.047	0.41	0.041	0.066	0.223	0.138	0.334
UCDX4_H1_700_30x30_1.1	Horse	700	0.011	0.045	0.426	0.048	0.065	0.23	0.136	0.344
UCDX4_H1_700_30x30_1.2		700	0.013	0.042	0.333	0.044	0.058	0.196	0.12	0.277
UCDX4_H2_700_30x30_1.1	Horse	700	0.006	0.048	0.441	0.042	0.059	0.209	0.148	0.357
UCDX4_H2_700_30x30_1.2		700	0.008	0.049	0.448	0.043	0.06	0.237	0.149	0.359
UCDX4_H3_700_30x30_1.1	Horse	700	0.01	0.05	0.532	0.044	0.079	0.0263	0.151	0.409

UCDX4_H3_700_30x30_1.2	Horse	700	0.01	0.048	0.441	0.048	0.072	0.0246	0.141	0.346
UCDX4_H1_700_50_1.1	Horse	700	0.01	0.049	0.443	0.044	0.059	0.233	0.14	0.336
UCDX4_H1_700_50_1.2		700	0.007	0.04	0.293	0.036	0.048	0.18	0.111	0.248
UCDX4_1505_800_30_1.1	Cow	800	0.004	0.01	0.157	0.02	0.044	0.103	0.07	0.147
UCDX4_1505_800_30_1.2		800	0.004	0.01	0.157	0.02	0.033	0.103	0.07	0.147
UCDX4_1501_800_30_1.1	Cow	800	0.009	0.019	0.23	0.029	0.052	0.135	0.093	0.21
UCDX4_1501_800_30_1.2		800	0.009	0.017	0.208	0.027	0.048	0.124	0.083	0.194
UCDX4_H1_800_30_1.1	Horse	800	0.012	0.042	0.448	0.041	0.068	0.24	0.146	0.369
UCDX4_H1_800_30_1.2		800	0.012	0.039	0.398	0.039	0.063	0.222	0.14	0.328
UCDX4_1505_900_30_1.1	Cow	900	0.003	0.006	0.258	0.018	0.048	0.163	0.1	0.242
UCDX4_1505_900_30_1.2		900	0.005	0.008	0.277	0.022	0.051	0.161	0.106	0.26
UCDX4_1500_900_30_1.1	Cow	900	0.011	0.013	0.466	0.021	0.119	0.259	0.138	0.395
UCDX4_1500_900_30_1.2		900	0.01	0.013	0.406	0.018	0.106	0.239	0.137	0.354
UCDX4_H1_900_30_1.1	Horse	900	0.007	0.026	0.446	0.033	0.066	0.152	0.152	0.378
UCDX4_H1_900_30_1.2		900	0.011	0.029	0.467	0.036	0.068	0.156	0.156	0.385
UCDX4_H5_900_30_1.1	Horse	900	0.007	0.031	0.562	0.041	0.064	0.0283	0.177	0.439
UCDX4_H5_900_30_1.2		900	0.006	0.028	0.515	0.039	0.061	0.0267	0.125	0.408
UCDX4_1505_1000_30_1.1	Cow	1000	0.0002	0.001	0.119	0.02	0.024	0.095	0.066	0.135
UCDX4_1505_1000_30_1.2		1000	-0.0013	0	0.101	0.018	0.019	0.082	0.56	0.12
UCDX4_1500_1000_30_1.1	Cow	1000	0	0.013	0.105	0.017	0.061	0.116	0.063	0.159
UCDX4_1500_1000_30_1.2		1000	0.016	0.016	0.279	0.024	0.096	0.188	0.101	0.271
UCDX4_1501_1000_30_1.1	Cow	1000	0.005	0.018	0.137	0.026	0.033	0.086	0.068	0.136
UCDX4_1501_1000_30_1.2		1000	0.004	0.019	0.133	0.026	0.032	0.084	0.067	0.126
UCDX4_H0_1000_30_1.1	Horse	1000	0.012	0.013	0.313	0.024	0.049	0.188	0.127	0.288
UCDX4_H0_1000_30_1.2		1000	0.014	0.015	0.372	0.029	0.057	0.223	0.148	0.33
UCDX4_H1_1000_30_1.1	Horse	1000	0.004	0.007	0.362	0.023	0.052	0.215	0.144	0.336
UCDX4_H1_1000_30_1.2		1000	0.006	0.008	0.316	0.021	0.048	0.205	0.133	0.295
UCDX4_1505_1100_30_1.1	Cow	1100	-0.00249	-0.018	0.0612	0.0212	0.0093	0.0587	0.0463	0.0801
UCDX4_1505_1100_30_1.2		1100	-0.00249	-0.0019	0.0624	0.0176	0.0095	0.06	0.0476	0.081
UCDX4_1500_1100_30_1.1	Cow	1100	0.006	0.006	0.244	0.031	0.043	0.171	0.129	0.235
UCDX4_1500_1100_30_1.2		1100	0.007	0.008	0.288	0.034	0.045	0.196	0.162	0.285

UCDX4_H1_1100_30_1.1	Horse	1100	0.014	0.016	0.303	0.038	0.048	0.195	0.148	0.277
UCDX4_H1_1100_30_1.2		1100	0.013	0.015	0.274	0.024	0.06	0.191	0.148	0.258
UCDX4_1505_1200_30_1.1	Cow	1200	-0.0039	-0.0033	0.0498	0.0249	0.0079	0.0373	0.0441	0.0654
UCDX4_1505_1200_30_1.2		1200	-0.002	-0.0017	0.0586	0.0288	0.0101	0.0559	0.0503	0.0734
UCDX4_1500_1200_30_1.1	Cow	1200	0.013	0.011	0.0249	0.045	0.05	0.183	0.162	0.247
UCDX4_1500_1200_30_1.2		1200	0	0.011	0.209	0.047	0.04	0.162	0.142	0.211
UCDX4_1501_1200_30_1.1	Cow	1200	0.0063	0.061	0.046	0.0268	0.0152	0.0455	0.0424	0.0604
UCDX4_1501_1200_30_1.2		1200	0.0078	0.077	0.053	0.0306	0.0193	0.0499	0.0455	0.0641
UCDX4_H1_1200_30_1.1	Horse	1200	0.0108	0.0109	0.0384	0.0222	0.0228	0.0383	0.0359	0.0502
UCDX4_H1_1200_30_1.2		1200	0.011	0.011	0.0549	0.0286	0.0233	0.0502	0.0454	0.067
UCDX4_H2_1200_30_1.1	Horse	1200	0.015	0.016	0.157	0.048	0.057	0.116	0.108	0.173
UCDX4_H2_1200_30_1.2		1200	0.016	0.017	0.158	0.048	0.045	0.121	0.112	0.176
