# Supplementary material for: Characterization of structural changes in modern and archaeological burnt bone: Implications for differential preservation bias
Source: PLoS One. 2021 Jul 28;16(7):e0254529. doi: 10.1371/journal.pone.0254529 (PMC8318310; doi:10.1371/journal.pone.0254529)
Supplement: S2 Table — (PDF) [file pone.0254529.s003.pdf]

S2 Table: Calculated experimental modern sample values of FTIR C/P, IRSF, and bioapatite crystallite size as measured from XRD.

			FTIR-ATR		XRD	
			C/P	SF	Crystallite size	
Experimental Sample	Species	Temperature (˚C)	(1415 cm -1 /1035 cm-1)	(565 cm-1+605 cm -1)/ 590 cm-1	Angstrom	+/-
UCDX4_1505_unburnt_1.1	Cow	25	0.281	2.59	88	6
UCDX4_1505_unburnt_1.2		25	0.282	2.68		
UCDX4_1500_unburnt_1.1	Cow	25	0.328	2.62		
UCDX4_1500_unburnt_1.2		25	0.321	2.59		
UCDX4_1501_unburnt_1.1	Cow	25	0.131	3.01		
UCDX4_1501_unburnt_1.2		25	0.143	2.98		
UCDX4_2120_unburnt_1.1	Cow	25	0.378	2.5		
UCDX4_2120_unburnt_1.2		25	0.388	2.45		
UCDX4_4101_unburnt_1.1	Horse	25	0.354	2.54		
UCDX4_4101_unburnt_1.2		25	0.375	2.57		
UCDX4_1505_100_30_1.1	Cow	100	0.297	2.57	80	5
UCDX4_1505_100_30_1.2		100	0.0367	2.58		
UCDX4_H1_100_30_1.1	Horse	100	0.445	2.43	79	8
UCDX4_H1_100_30_1.2		100	0.49	2.41		
UCDX4_1505_200_30_1.1	Cow	200	0.313	2.65	76	18
UCDX4_1505_200_30_1.2		200	0.162	2.8		
UCDX4_H1_200_30_1.1	Hose	200	0.371	2.53	90	5
UCDX4_H1_200_30_1.2		200	0.388	2.53		
UCDX4_H2_200_30_1.1	Horse	200	0.364	2.63	114	8
UCDX4_H2_200_30_1.2		200	0.377	2.64		
UCDX4_1505_300_30_1.1	Cow	300	0.254	2.81	76	18
UCDX4_1505_300_30_1.2		300	0.13	3.11		
UCDX4_1500_300_30_1.1	Cow	300	0.207	2.85	88	5
UCDX4_1500_300_30_1.2		300	0.201	2.97		
UCDX4_H1_300_30_1.1	Horse	300	0.274	2.76	87	5
UCDX4_H1_300_30_1.2		300	0.278	2.71		
UCDX4_H2_300_30_1.1	Horse	300	0.278		98	6
UCDX4_H2_300_30_1.2		300	0.272			
UCDX4_H2T2_300_30_1.1	Horse	300	0.28	2.93	98	7
UCDX4_H2T2_300_30_1.2		300	0.267	2.89		
UCDX4_1505_300_30x30_1.1	Cow	300	0.207	2.94	83	6
UCDX4_1505_300_30x30_1.2		300	0.207	2.81		
UCDX4_H1_300_30x30_1.1	Horse	300	0.21	2.95	84	6
UCDX4_H1_300_30x30_1.2		300	0.235	1.8		
UCDX4_H1T2_300_30x30_1.1	Horse	300	0.257	2.87	104	9
UCDX4_H1T2_300_30x30_1.2		300	0.25	2.49		
UCDX4_H1_300_50_1.1	Horse	300	0.267	2.68	90	6
UCDX4_H1_300_50_1.2		300	0.275	2.72		
UCDX4_H2_300_50_1.1	Horse	300	0.237	2.92		
UCDX4_H2_300_50_1.2		300	0.226	3		
UCDX4_1505_400_30_1.1	Cow	400	0.158	2.97	83	6
UCDX4_1505_400_30_1.2		400	0.192	3		
UCDX4_1500_400_30_1.1	Cow	400	0.183	2.92	88	7
UCDX4_1500_400_30_1.2		400	0.176	3.02		
UCDX4_1501_400_30_1.1	Cow	400	0.21	2.82	87	3
UCDX4_1501_400_30_1.2		400	0.222	2.74		
UCDX4_H1_400_30_1.1	Horse	400	0.211	2.97	90	6
UCDX4_H1_400_30_1.2		400	0.22	2.91		
UCDX4_H2_400_30_1.1	Horse	400	0.226	3.1	[illegible]	5

UCDX4_H2_400_30_1.2	Horse	400	0.228	1.23	92	[illegible]
UCDX4_1505_500_30_1.1	Cow	500	0.158	3.14	90	6
UCDX4_1505_500_30_1.2		500	0.157	3.06		
UCDX4_1500_500_30_1.1	Cow	500	0.171	3.08	94	7
UCDX4_1500_500_30_1.2		500	0.174	3.07		
UCDX4_H1_500_30_1.1	Horse	500	0.257	2.93	105	7
UCDX4_H1_500_30_1.2		500	0.259	2.87		
UCDX4_H2_500_30_1.1	Horse	500	0.203	3.02	85	4
UCDX4_H2_500_30_1.2		500	0.215	3.02		
UCDX4_1505_600_30_1.1	Cow	600	0.058	3.54	99	6
UCDX4_1505_600_30_1.2		600	0.157	3.45		
UCDX4_1500_600_30_1.1	Cow	600	0.113	3.47	109	4
UCDX4_1500_600_30_1.2		600	0.113	3.59		
UCDX4_1501_600_30_1.1	Cow	600	0.144	3.2	107	7
UCDX4_1501_600_30_1.2		600	0.139	3.25		
UCDX4_H1_600_30_1.1	Horse	600	0.229	3.13	109	7
UCDX4_H1_600_30_1.2		600	0.231	3.18		
UCDX4_H2_600_30_1.1	Horse	600	0.165	3.46	99	13
UCDX4_H2_600_30_1.2		600	0.186	3.36		
UCDX4_H4_600_30_1.1	Horse	600	0.174	3.44	92	6
UCDX4_H4_600_30_1.2		600	0.197	3.26		
UCDX4_1505_700_30_1.1	Cow	700	0.051	3.59	256	15
UCDX4_1505_700_30_1.2		700	0.08	3.68		
UCDX4_1500_700_30_1.1	Cow	700	0.081	3.77	177	40
UCDX4_1500_700_30_1.2		700	0.085	3.66		
UCDX4_1501_700_30_1.1	Cow	700	0.079	3.42	376	18
UCDX4_1501_700_30_1.2		700	0.078	3.38		
UCDX4_H1_700_30_1.1	Horse	700	0.108	4.19	389	8
UCDX4_H1T1_700_30_1.1	Horse	700	0.104	4.17	462	12
UCDX4_H1T1_700_30_1.2		700	0.125	4.11		
UCDX4_H2_700_30_1.1	Horse	700	0.141	3.68	289	17
UCDX4_H2_700_30_1.2		700	0.171	3.52		
UCDX4_1505_700_30x30_1.1	Cow	700	0.065	3.87	272	21
UCDX4_1505_700_30x30_1.2		700	0.079	3.75		
UCDX4_1501_700_30x30_1.1	Cow	700	0.068	4.1		
UCDX4_1501_700_30x30_1.2		700	0.079	4.01		
UCDX4_H0_700_30x30_1.1	Horse	700	0.11	4.29	653	15
UCDX4_H0_700_30x30_1.2		700	0.114	4.03		
UCDX4_H1_700_30x30_1.1	Horse	700	0.105	4.22	564	13
UCDX4_H1_700_30x30_1.2		700	0.126	3.94		
UCDX4_H2_700_30x30_1.1	Horse	700	0.108	3.82	583	19
UCDX4_H2_700_30x30_1.2		700	0.109	4		
UCDX4_H3_700_30x30_1.1	Horse	700	0.093	2.88	583	17
UCDX4_H3_700_30x30_1.2		700	0.108	2.62		
UCDX4_H1_700_50_1.1	Horse	700	0.11	4.06	350	70
UCDX4_H1_700_50_1.2		700	0.136	3.85		
UCDX4_1505_800_30_1.1	Cow	800	0.063	3.57	561	37
UCDX4_1505_800_30_1.2		800	0.063	3.57		
UCDX4_1501_800_30_1.1	Cow	800	0.082	3.7	551	47
UCDX4_1501_800_30_1.2		800	0.081	3.83		
UCDX4_H1_800_30_1.1	Horse	800	0.093	4.17	641	21
UCDX4_H1_800_30_1.2		800	0.097	3.92		
UCDX4_1505_900_30_1.1	Cow	900	0.023	4.05	868	57
UCDX4_1505_900_30_1.2		900	0.028	3.97		

UCDX4_1500_900_30_1.1	Cow	900	0.032	4.74	777	29
UCDX4_1500_900_30_1.2		900	0.032	4.3		
UCDX4_H1_900_30_1.1	Horse	900	0.058	4.05	662	32
UCDX4_H1_900_30_1.2		900	0.062	4.01		
UCDX4_H5_900_30_1.1	Horse	900	0.055	2.64	733	33
UCDX4_H5_900_30_1.2		900	0.054	3.47		
UCDX4_1505_1000_30_1.1	Cow	1000	0.008	3.48	827	37
UCDX4_1505_1000_30_1.2		1000	0	3.6		
UCDX4_1500_1000_30_1.1	Cow	1000	0.084	4.36	794	22
UCDX4_1500_1000_30_1.2		1000	0.057	4.54		
UCDX4_1501_1000_30_1.1	Cow	1000	0.131	3.26	96	3
UCDX4_1501_1000_30_1.2		1000	0.142	3.13		
UCDX4_H0_1000_30_1.1	Horse	1000	0.041	3.74	901	53
UCDX4_H0_1000_30_1.2		1000	0.04	3.73		
UCDX4_H1_1000_30_1.1	Horse	1000	0.019	3.82	809	50
UCDX4_H1_1000_30_1.2		1000	0.025	3.75		
UCDX4_1505_1100_30_1.1	Cow	1100	-0.029	2.99	>1000	
UCDX4_1505_1100_30_1.2		1100	-0.03	2.96		
UCDX4_1500_1100_30_1.1	Cow	1100	0.024	3.14	582	17
UCDX4_1500_1100_30_1.2		1100	0.027	2.96		
UCDX4_H1_1100_30_1.1	Horse	1100	0.052	3.18	557	23
UCDX4_H1_1100_30_1.2		1100	0.054	3.03		
UCDX4_1505_1200_30_1.1	Cow	1200	-0.066	2.32	852	76
UCDX4_1505_1200_30_1.2		1200	-0.029	2.57		
UCDX4_1500_1200_30_1.1	Cow	1200	0.0441	2.65	613	45
UCDX4_1500_1200_30_1.2		1200	0.0526	2.62		
UCDX4_1501_1200_30_1.1	Cow	1200	0.132	2.49	888	17
UCDX4_1501_1200_30_1.2		1200	0.145	2.5		
UCDX4_H1_1200_30_1.1	Horse	1200	0.283	2.46	637	61
UCDX4_H1_1200_30_1.2		1200	0.2	2.58		
UCDX4_H2_1200_30_1.1	Horse	1200	0.101	2.67	517	50
UCDX4_H2_1200_30_1.2		1200	0.107	2.65		
