# Supplementary material for: Characterization of structural changes in modern and archaeological burnt bone: Implications for differential preservation bias
Source: PLoS One. 2021 Jul 28;16(7):e0254529. doi: 10.1371/journal.pone.0254529 (PMC8318310; doi:10.1371/journal.pone.0254529)
Supplement: S3 Table — (PDF) [file pone.0254529.s004.pdf]

S3 Table: FTIR-ATR T-17 Unit 3 archaeological sample relevant peak height values.

Sample	Burning Stage	1650 cm^{-1}	1415 cm^{-1}	1035 cm^{-1}	874 cm^{-1}	625 cm^{-1}	605 cm^{-1}	595 cm^{-1}	565 cm^{-1}
T17-779 T1.1	Stage 0	0.0281	0.0473	0.1141	0.0442	0.0448	0.0844	0.075	0.1091
T17-779 T1.2	Stage 0	0.0245	0.0419	0.1004	0.0387	0.0415	0.0762	0.0655	0.097
T17-754 T1.1	Stage 0	0.0224	0.0369	0.0844	0.0357	0.0351	0.0672	0.06	0.0892
T17-754 T1.2	Stage 0	0.023	0.0336	0.0834	0.0352	0.0327	0.0657	0.0575	0.087
T17-747a T1.1	Stage 0	0.022	0.033	0.0688	0.0348	0.0325	0.059	0.0527	0.0743
T17-747a T1.2	Stage 0	0.0249	0.0405	0.0917	0.041	0.037	0.719	0.064	0.0919
T17-757a T1.1	Stage 0	0.0098	0.0179	0.0342	0.0189	0.0165	0.0287	0.0267	0.0377
T17-757a T1.2	Stage 0	0.0127	0.0253	0.0634	0.0279	0.0241	0.0526	0.0461	0.0681
T17-496a T1.1	Stage 0	0.021	0.039	0.105	0.042	0.039	0.086	0.076	0.115
T17-496a T1.2	Stage 0	0.0229	0.0375	0.0849	0.0394	0.0372	0.0698	0.0629	0.0902
T17-542 T1.1	Stage 0	0.024	0.046	0.124	0.054	0.044	0.09	0.08	0.12
T17-542 T1.2	Stage 0	0.022	0.042	0.115	0.047	0.043	0.093	0.085	0.124
T17-496b T1.1	Stage 1	0.008	0.031	0.111	0.042	0.036	0.075	0.068	0.101
T17-496b T1.2	Stage 1	0.0116	0.0299	0.0938	0.0346	0.0327	0.0664	0.0589	0.0902
T17-761 T1.1	Stage 1	0.0119	0.0245	0.0682	0.0276	0.025	0.0549	0.0491	0.0744
T17-761 T1.2	Stage 1	0.116	0.025	0.0656	0.0257	0.0258	0.0552	0.0472	0.0696
T17-611 T1.1	Stage 2	0.211	0.0349	0.0878	0.0372	0.0352	0.0719	0.0628	0.1054
T17-611 T1.2	Stage 2	0.0199	0.0368	0.1012	0.0379	0.0364	0.08	0.0664	0.1069
T17-487 T1.1	Stage 2	0.0119	0.0265	0.0782	0.0299	0.0272	0.0593	0.0532	0.085
T17-487 T1.2	Stage 2	0.0097	0.0195	0.0518	0.0215	0.0209	0.0446	0.0384	0.0594
T17-816 T1.1	Stage 2	0.017	0.039	0.111	0.044	0.041	0.081	0.073	0.109
T17-816 T1.2	Stage 2	0.016	0.042	0.117	0.047	0.042	0.084	0.075	0.114
T17-651 T1.1	Stage 3	0.0107	0.0226	0.0805	0.027	0.027	0.0625	0.0507	0.0844
T17-651 T1.2	Stage 3	0.0102	0.0229	0.0523	0.0269	0.0275	0.0646	0.0519	0.0872
T17-511 T1.1	Stage 3	0.011	0.03	0.104	0.029	0.033	0.063	0.0836	0.0515
T17-511 T1.2	Stage 3	0.0131	0.0268	0.0813	0.0274	0.0268	0.63	0.0522	0.0836
T17-720a T1.1	Stage 4	0.018	0.035	0.114	0.039	0.034	0.086	0.112	0.068
T17-720a T1.2	Stage 4	0.018	0.037	0.12	0.038	0.035	0.089	0.119	0.07
T17-757b T1.1	Stage 5	0.009	0.022	0.13	0.025	0.037	0.088	0.057	0.126
T17-757b T1.2	Stage 5	0.01	0.027	0.152	0.029	0.04	0.1	0.065	0.142
T17-675 T1.1	Stage 5	0.008	0.051	0.265	0.047	0.055	0.15	0.087	0.212
T17-675 T1.2	Stage 5	0.006	0.037	0.188	0.035	0.044	0.119	0.064	0.156
T17-580 T1.1	Stage 6	0.009	0.026	0.25	0.045	0.062	0.186	0.07	0.25
T17-580 T1.2	Stage 6	0.012	0.028	0.249	0.045	0.066	0.173	0.1	0.246
T17-682 T1.1	Stage 6	0.007	0.048	0.251	0.049	0.062	0.15	0.091	0.219
T17-682 T1.2	Stage 6	0.007	0.048	0.258	0.054	0.058	0.147	0.088	0.216
T17-637 T1.1	Stage 6	0.011	0.023	0.152	0.039	0.053	0.107	0.064	0.156
T17-637 T1.2	Stage 6	0.009	0.02	0.143	0.037	0.051	0.099	0.059	0.147
T17-720b T1.1	Stage 6	0.023	0.051	0.432	0.049	0.118	0.244	0.137	0.381
T17-720b T1.2	Stage 6	0.023	0.056	0.432	0.053	0.124	0.252	0.147	0.392
