# Supplementary material for: Characterization of structural changes in modern and archaeological burnt bone: Implications for differential preservation bias
Source: PLoS One. 2021 Jul 28;16(7):e0254529. doi: 10.1371/journal.pone.0254529 (PMC8318310; doi:10.1371/journal.pone.0254529)
Supplement: S4 Table — (PDF) [file pone.0254529.s005.pdf]

S4 Table: Calculated archaeological T-17 Unit 3 C/P and IRSF values, and bioapatite crystal size averages as measured from XRD.

		FTIR-ATR		XRD	
		C/P	IRSF	Crystallite size	
Archaeological Sample	Burning Stage	(1415 cm -1 /1035 cm-1)	(565 cm-1+605 cm -1)/ 590 cm-1	Angstrom	+/-
T17-779	Stage 0	0.415	2.61	99	5
T17-754	Stage 0	0.42	2.63	95	5
T17-747	Stage 0	0.46	2.54	96	4
T17-757a	Stage 0	0.461	2.55	83	4
T17-496a	Stage 0	0.406	2.59	107	5
T17-542	Stage 0	0.368	2.58	99	4
T17-496b	Stage 1	0.299	2.62	97	6
T17-761	Stage 1	0.37	2.63	111	5
T17-611	Stage 2	0.38	2.75	112	6
T17-487	Stage 2	0.357	2.71	119	5
T17-816	Stage 2	0.355	2.62	89	3
T17-651	Stage 3	0.279	2.91	112	5
T17-511	Stage 3	0.329	2.84		/
T17-720a	Stage 4	0.307	2.94	118	5
T17-757b	Stage 5	0.173	3.73	167	6
T17-675	Stage 5	0.19	4.22	345	20
T17-580	Stage 6	0.108	5.2	600	23
T17-682	Stage 6	0.188	4.08	366	18
T17-637	Stage 6	0.145	4.13	813	39
T17-720b	Stage 6	0.123	4.47	881	26
