# Supplementary material for: Characterization of structural changes in modern and archaeological burnt bone: Implications for differential preservation bias
Source: PLoS One. 2021 Jul 28;16(7):e0254529. doi: 10.1371/journal.pone.0254529 (PMC8318310; doi:10.1371/journal.pone.0254529)
Supplement: Attachment — Submitted filename: PONE-D-20-19845_reviewer1_27july.pdf [file pone.0254529.s006.pdf]

PLOS ONE

Characterization of structural changes in modern and archaeological burnt bone: Implications for differential preservation bias

--Manuscript Draft--

**Manuscript Number:**	PONE-D-20-19845
**Article Type:**	Research Article
**Full Title:**	Characterization of structural changes in modern and archaeological burnt bone: Implications for differential preservation bias
**Short Title:**	Crystallite size growth in modern and archaeological burnt bone
**Corresponding Author:**	Giulia Gallo<br>University of California Davis<br>Davis, CA UNITED STATES
**Keywords:**	Archaeology; Zooarchaeology; Bone; Burnt Bone; FTIR; XRD
**Abstract:**	The degree of alteration to the crystallinity, porosity, and organic content of burnt bone is dependent on the intensity of burning. The influence of the burial environment on bone preservation is partly a consequence of the extent of these changes. Bones which are vulnerable to aqueous dissolution are of particular interest to this study, as groundwater interaction is demonstrated to highly impact the preservation of unburnt bone but has not yet been addressed for bone burnt to different temperatures. Here we describe the timing of changing structural and compositional qualities of experimentally burnt bone (100-1200˚C in oxygen atmospheres) alongside an open-air archaeological assemblage of burnt bone from a fluctuating hydraulic environment in northern Mongolia dating back to 30 ka. Trends in crystallite growth and loss of organics are described with Fourier transform infrared spectroscopy with attenuated total reflectance attachment (FTIR-ATR) as well as X-Ray Diffraction (XRD) and demonstrate the vulnerabilities of carbonized bone to groundwater movement. Organic protection by collagen or large bioapatite crystal sizes are found to likely have a strong influence on bone preservation, describing a potential bias in archaeological assemblages of burnt bone.
**Order of Authors:**	Giulia Gallo
	Matthew Fyhrie
	Cleantha Paine
	Sergey V. Ushakov
	Masami Izuho
	Gunchinsuren Byambaa
	Nicolas Zwyns
	Alexandra Navrotsky
**Additional Information:**	
**Question**	**Response**
**Financial Disclosure**<br><br>Enter a financial disclosure statement that describes the sources of funding for the work included in this submission. Review the submission guidelines for detailed requirements. View published research articles from *PLOS ONE* for specific examples.	NZ, Grant #156074, National Science Foundation (https://www.nsf.gov/awardsearch/showAward?AWD_ID=1560784)<br>MI PaleoAsia Project Grant No. 1802, FY2016–2020) from the Ministry of Education, Culture, Sports, Science and Technology, Japan (“Cultural history of PaleoAsia: Integrative research on the formative processes of modern human cultures in Asia,” directed by Yoshihiro Nishiaki) (http://paleoasia.jp/en/)<br>GG UC Davis Cluster Grant “Archaeology and Soil Science Synergy" (https://dhi.ucdavis.edu/events/2019-2020-dhi-transcollege-research-clusters-call-proposals)<br>The funders had no role in study design, data collection and analysis, decision to

Powered by Editorial Manager® and ProduXion Manager® from Aries Systems Corporation

This statement is required for submission and **will appear in the published article** if the submission is accepted. Please make sure it is accurate.<br><br>**Unfunded studies**<br>Enter: *The author(s) received no specific funding for this work.*<br><br>**Funded studies**<br>Enter a statement with the following details:<br>• Initials of the authors who received each award<br>• Grant numbers awarded to each author<br>• The full name of each funder<br>• URL of each funder website<br>• Did the sponsors or funders play any role in the study design, data collection and analysis, decision to publish, or preparation of the manuscript?<br>• **NO** - Include this sentence at the end of your statement: *The funders had no role in study design, data collection and analysis, decision to publish, or preparation of the manuscript.*<br>• **YES** - Specify the role(s) played.<br><br>* typeset	publish, or preparation of the manuscript.
**Competing Interests**<br><br>Use the instructions below to enter a competing interest statement for this submission. On behalf of all authors, disclose any competing interests that could be perceived to bias this work—acknowledging all financial support and any other relevant financial or non-financial competing interests.<br><br>This statement **will appear in the published article** if the submission is accepted. Please make sure it is accurate. View published research articles from *PLOS ONE* for specific examples.	The authors have declared that no competing interests exist.

**NO authors have competing interests**<br><br>Enter: *The authors have declared that no competing interests exist.*<br><br>**Authors with competing interests**<br><br>Enter competing interest details beginning with this statement:<br><br>*I have read the journal's policy and the authors of this manuscript have the following competing interests: [insert competing interests here]*<br><br>* typeset	
**Ethics Statement**<br><br>Enter an ethics statement for this submission. This statement is required if the study involved:<br><br>• Human participants<br>• Human specimens or tissue<br>• Vertebrate animals or cephalopods<br>• Vertebrate embryos or tissues<br>• Field research<br><br>Write "N/A" if the submission does not require an ethics statement.<br><br>General guidance is provided below. Consult the submission guidelines for detailed instructions. **Make sure that all information entered here is included in the Methods section of the manuscript.**	This study was carried out with cow bones procured from a local butcher in Sacramento, California, as well as bones removed from horses which were humanely euthanized for purposes other than this study and donated for scientific purposes at the Center for Equine Health at the UC Davis School of Veterinary Medicine in Davis, California.<br><br>The cow bones utilized in this study were from animals slaughtered for meat, and were stunned to render the animal unconscious and unaware of pain, according to the Humane Slaughter Association.<br><br>The humane euthanasia program at the Center for Equine Health at the UC Davis School of Veterinary Medicine is approved by the American Veterinary Medical Association (AVMA) and is done with great care to ameliorate animal suffering. The horses which were humanely euthanized for health purposes were brought to the Center of Equine Health following either a poor prognosis and quality of life following either extreme cervical osteoarthritis or a femur fracture. These horses were euthanized with an intravenous injection of pentobarbital, with prior light sedation and a local anesthetic used to place the intravenous catheter. Specific studies by the comparative neurology group at the UC Davis School of Veterinary Science have determined that the horse rapidly loses all perception of consciousness within ten seconds of administration, and the animals feel no pain or anxiety.<br><br>Subsequent to euthanasia, the Center for Equine Health allowed for tissue collection for necessary scientific purposes. The forelimbs were taken by the JD Wheat Orthopedic Laboratory, where the metacarpals were removed and given to this study presented in this manuscript. Upon inquiry, the JD Wheat Laboratory provided a statement that there was not a committee to approve the use of the legs, as the horses were not euthanized for the purpose of the study.

Powered by Editorial Manager® and ProduXion Manager® from Aries Systems Corporation

**Format for specific study types**<br><br>**Human Subject Research (involving human participants and/or tissue)**<br>• Give the name of the institutional review board or ethics committee that approved the study<br>• Include the approval number and/or a statement indicating approval of this research<br>• Indicate the form of consent obtained (written/oral) or the reason that consent was not obtained (e.g. the data were analyzed anonymously)<br><br>**Animal Research (involving vertebrate animals, embryos or tissues)**<br>• Provide the name of the Institutional Animal Care and Use Committee (IACUC) or other relevant ethics board that reviewed the study protocol, and indicate whether they approved this research or granted a formal waiver of ethical approval<br>• Include an approval number if one was obtained<br>• If the study involved *non-human primates*, add *additional details* about animal welfare and steps taken to ameliorate suffering<br>• If anesthesia, euthanasia, or any kind of animal sacrifice is part of the study, include briefly which substances and/or methods were applied<br><br>**Field Research**<br><br>Include the following details if this study involves the collection of plant, animal, or other materials from a natural setting:<br>• Field permit number<br>• Name of the institution or relevant body that granted permission	
**Data Availability**<br><br>Authors are required to make all data underlying the findings described fully available, without restriction, and from the time of publication. PLOS allows rare exceptions to address legal and ethical concerns. See the PLOS Data Policy and FAQ for detailed information.	Yes - all data are fully available without restriction

Powered by Editorial Manager® and ProduXion Manager® from Aries Systems Corporation

A Data Availability Statement describing where the data can be found is required at submission. Your answers to this question constitute the Data Availability Statement and **will be published in the article**, if accepted.<br><br>**Important:** Stating 'data available on request from the author' is not sufficient. If your data are only available upon request, select 'No' for the first question and explain your exceptional situation in the text box.<br><br>Do the authors confirm that all data underlying the findings described in their manuscript are fully available without restriction?	
**Describe where the data may be found in full sentences. If you are copying our sample text, replace any instances of XXX with the appropriate details.**<br><br>• If the data are **held or will be held in a public repository**, include URLs, accession numbers or DOIs. If this information will only be available after acceptance, indicate this by ticking the box below. For example: *All XXX files are available from the XXX database (accession number(s) XXX, XXX.)*.<br>• If the data are all contained **within the manuscript and/or Supporting Information files**, enter the following: *All relevant data are within the manuscript and its Supporting Information files.*<br>• If neither of these applies but you are able to provide **details of access elsewhere**, with or without limitations, please do so. For example:<br><br>*Data cannot be shared publicly because of [XXX]. Data are available from the XXX Institutional Data Access / Ethics Committee (contact via XXX) for researchers who meet the criteria for access to confidential data.*<br><br>*The data underlying the results presented in the study are available from (include the name of the third party*	All relevant data are within the manuscript and its Supporting Information files.

and contact information or URL).<br>• This text is appropriate if the data are owned by a third party and authors do not have permission to share the data.<br><br>* typeset	
Additional data availability information:	

Powered by Editorial Manager® and ProduXion Manager® from Aries Systems Corporation

Manuscript

Click here to access/download;Manuscript;Characterization_Submission_PLo

Title: Characterization of structural changes in modern and archaeological burnt bone: Implications for differential preservation bias

Authors: Giulia Gallo[1,2*¶,] Matthew Fyhrie[1¶,] Cleantha Paine[3,4¶], Sergey V. Ushakov[5¶], Masami Izuho[6&], Gunchinsuren Byambaa[7&], Nicolas Zwyns[1,2,8¶], Alexandra Navrotsky[5&]

Affiliations:

1- Department of Anthropology, University of California Davis, 95616, Davis CA, USA

2- Center for Experimental Archaeology at Davis, University of California Davis, 95616, Davis CA, USA

3- Department of Archaeology, University of Cambridge, Cambridge, UK

4- Archaeology Institute University of the Highlands and Islands, Kirkwall, UK

5- School of Molecular Sciences and Center for Materials of the Universe, Arizona State University, 85287, Tempe AZ, USA

6- Tokyo Metropolitan University, Tokyo, 192-0397, Japan

7- Institute for History and Archaeology, Mongolia Academy of Science, Khukov Street-77, Ulaanbaatar, Mongolia

8- Department of Human Evolution, Max Planck Institute for Evolutionary Anthropology, 04103, Leipzig, Germany.

*Corresponding author

Email: gtgallo@ucdavis.edu

[¶] These authors contributed equally to this work

[&]These authors contributed equally to this work

Abstract:

The degree of alteration to the crystallinity, porosity, and organic content of burnt bone is dependent on the intensity of burning. The influence of the burial environment on bone preservation is partly a consequence of the extent of these changes. Bones which are vulnerable to aqueous dissolution are of particular interest to this study, as groundwater interaction is demonstrated to highly impact the preservation of unburnt bone but has not yet been addressed for bone burnt to different temperatures. Here we describe the timing of changing structural and compositional qualities of experimentally burnt bone (100-1200˚C in oxygen atmospheres) alongside an open-air archaeological assemblage of burnt bone from a fluctuating hydraulic environment in northern Mongolia dating back to 30 ka. Trends in crystallite growth and loss of organics are described with Fourier transform infrared spectroscopy with attenuated total reflectance attachment (FTIR-ATR) as well as X-Ray Diffraction (XRD) and demonstrate the vulnerabilities of carbonized bone to groundwater movement. Organic protection by collagen or large bioapatite crystal sizes are found to likely have a strong influence on bone preservation, describing a potential bias in archaeological assemblages of burnt bone.

Introduction:

The extrinsic and intrinsic variables which affect bone preservation have been studied with regard to the impact of differences between compact and cancellous bone [1-4], juvenile and adult bone [5, 6], intra- and interspecies variation in bone size and density [7-9] and environmental conditions [10-15]. Evaluations of the taphonomic biases impacting the preservation of burnt bone have resulted in varied interpretations [16]. For example, highly burnt

calcined bone has been suggested to be both exceedingly mechanically fragile as well as capable of surviving in non-ideal conditions [17]. To date, the properties of burnt bone which may explain or inform these assumptions have not been directly addressed [17]. It is necessary to explore such relationships, as burnt bone is a valuable resource contributing to the study of ancient fire residue [17-20].

Bones burned by humans can be indicative of many social and economic behaviors and can contribute to archaeological studies identifying evidence for cremations [21, 22], bone fuel [16, 23-27], hygienic practices [16, 25], cooking and marrow warming [26], and locations of anthropogenic fire [20]. The thermal alteration of bone, therefore, has been the focus of many studies, with early investigations focusing on macroscopic changes in bone color and texture [17, 28]. This has led to the development of common-use zooarchaeological standards in codifying burnt archaeological bone assemblages, such as the Stiner et al. [17] scale of burning (Table 1). While it is recognized that bones burnt to different temperatures have different structural properties on the nano-, micro- and macro-scales, this variation within burnt fauna has not been addressed regarding biases in preservation nor investigated within an archaeological assemblage of burnt bone [17, 29]

Table 1: Burning intensity stages based on visual characterizations following Stiner et al. [17]

Burning Stage	Description
0	Not burnt
1	Slightly burnt, < 50% carbonized
2	Majority burnt, > 50% carbonized
3	Fully carbonized
4	Slightly highly burnt, < 50% calcined
5	Majority highly burnt, > 50% calcined
6	Fully calcined

The aim of this study is to review the modifications to bone related to burning and to specifically characterize the timing of heat induced growth of bone crystals and loss of organics that may influence the susceptibility of burnt bone to aqueous dissolution. These structural changes and the potential biases in preservation are described here within a modern assemblage of experimentally burnt bone as well as within an archaeological assemblage of burnt fauna which has a depositional history of fluctuating hydraulic regimes in a geographic area without abundant documentation of ancient fire. This integrated approach incorporating both a thermodynamic perspective of bone and inferences about taphonomic history is necessary to enhance our understanding of the links between the impact of the depositional environment and burnt bone survival and visibility for zooarchaeological studies of burnt fauna.

Background:

Bone

Bone is a composite material comprised of inorganic compounds (primarily hydroxyapatite), organics (primarily collagen) and water [30, 31]. These biomaterials are hierarchically arranged to provide the necessary mechanisms and structures that maintain the biological role of skeletal tissue: mechanical strength to transmit force and protect organs, and the regulation of homeostasis through ionic regulation [32, 33].

Bone mineral, or, bioapatite, is nonstoichiometric calcium phosphate apatite, $Ca_{10-x}(PO_4)_{6-x}(HPO_4,CO_3)_x(OH,1/2CO_3)_{2-x}$ with $0 \leq x \leq 2$ [32, 34]. The specific chemical compositions of bioapatite reflect diet, biological age, bone remodeling, and can vary within individuals and within skeletal elements themselves [33]. Compared to hydroxyapatite, bioapatite composition and microstructure are characterized by a higher degree of

nonstoichiometry, vacancies in the crystal lattice, nano-sized platelet shapes, and by the presence of a hydrated ionic layer [32, 35]. Nonstoichiometry in bioapatite is related to a low degree of crystallinity, in that the minerals are poorly ordered with various degrees of strain introduced by different sized crystallites and carbonate substitutions [32, 34, 36]. Type-A (CO_3 for OH) and the more prevalent Type-B (CO_3 for PO_4) are the most common substitutions in the crystal lattice and comprise approximately 5-8% of bone by weight [35].

In vivo bioapatite has extremely small, thin, plate-like morphology (1-7 nm thick, 15-200 nm length, and 10-80 nm width) and is cross-linked to collagen fibrils [34, 36, 37]. Water is found in bone as loose mobile water in the extracellular matrix and in void spaces to facilitate movement between collagen fibrils and minerals, as well as both incorporated within and around the organic and mineral components [32, 38]. Bioapatite specifically features water in a hydrated ionic layer around the crystalline core and in vacancies in the crystal lattice [32, 38]. This hydrated layer regulates homeostasis, as the surface layer of ions can be easily exchanged with the large, specific, and very reactive surface of the bone mineral, estimated at 240 m^2g [31, 36, 37, 39]. This is necessary to regulate ionic concentrations [22, 27, 28, 30]. The high surface to mass ratio and reactive hydrated layer of the small crystallites results in an exchange capacity with large sorption coefficients on the order of 10^6-10^8 [39-41].

Porosity

Bioapatite, collagen, and water molecules are organized on the microscale into Haversian systems: cylindrical structures of concentric lamellae surrounding an interior channel for a central blood canal [30, 33]. In addition to the Haversian canal, two other features constitute the typical porosity inherent in bone matrix: the lacunal-canaculi network, also known as resorption

bays (Howship's lacunae), which are large pores for osteoblast/osteoclast remodeling, and the smaller spaces created between collagen and apatite (collagen apatite porosity, also known as CAP) [33, 42]. Living bone is very porous, with around 12 % of bone volume comprised of pores [12]. The amount, size, and density of pores in compact bone is variable across and within elements, species, and age [33, 42]. Studies specifically addressing the mineral density of bone across species with computer tomography (CT) imaging have found that bone density is less variable between taxa than within taxa once differences in health or age of the animal are taken into account [8, 9]. With aging, mature bone experiences a decrease in mineral density, but immature bone exhibits overall the highest macro- and micro- porosity [6].

Diagenesis and Solubility

Diagenesis, the changes to bony tissue in burial environments, includes the integrated reactions of hydrolysis, microbial attack, and mineral recrystallization [15]. The rate and sequence of these processes are determined by many factors, including the nature of the depositional environment, and the age, element, and species of bone in life. Early taphonomic history and the initial arrangement and size distribution of the pores are particularly important for diagenetic processes, as higher degrees of porosity decrease protection and allow greater access to digenetic agents [6, 10-12, 43-45]. The extent of porosity inherent in a bone therefore has a large impact on the degree and rate of either bone decay or bone survival [12].

Other influential factors include the degree of flesh cover, groundwater interaction, and soil pH [6, 10-12, 15, 43-45]. Microbial attack is an active and immediate process especially for fleshed elements, accounting for a large amount of organic destruction [42, 45]. This is found to be especially true in warmer environments [43, 46]. Bacterial action also is more pronounced in

fleshed burials, and lowers the bone pH, which fosters beneficial conditions for later mineral recrystallization [43].

The specific interaction between hydrologic conditions and bone is hypothesized to be the most significant predicate of bioapatite dissolution [12, 15, 37]. Hydrolysis is a reaction with water that results in chemical disintegration, typically an early stage of diagenesis [12, 47]. The mechanisms of hydrolysis depend on the relationship between the mineral and organic components of bone and the available access to pores, as well as the water fluctuations in the depositional environment. Three hydrological regimes have been outlined to address the impact water can have on bone: diffusion, recharge, and hydraulic flow [12, 45]. For diffusion, a waterlogged environment with negligible water movement, the dissolution process is slowed, aiding bone preservation [12, 45]. For recharge regimes, in which the relative amounts of water changes, and for flow regimes, which are categorized as environments with water movement, there is a much higher potential for bone dissolution as water acts as an agent to dissolve and leach bone mineral and organics [12, 15, 45].

Bone dissolution is dependent on the loss of organic protection and the nature of the structure and surface characteristics of bone apatite [37]. Type I collagen itself is insoluble due to hydrophobic interactions, the highly ordered arrangement with other collagen fibers, and cross-linked arrangements with bone mineral [15]. Access to collagen and rate of collagen removal is mediated by the amount and size of pores, which are made available by bacterial action and recrystallization of the bone mineral [12, 39, 43, 44, 47, 48]. After death, this microbial action and the degradation of the protein chains into smaller peptides, or, gelatinization, effectively eliminates the organic components of bone and therefore the protection of bone mineral [7, 12, 46, 47].

Bone mineral microstructure is thermodynamically unstable. Without *in vivo* regulation, larger bioapatite crystals begin to spontaneously increase in size and become more ordered at the expense of smaller crystals [6, 37, 47, 49]. This process, Ostwald ripening, decreases the available surface area of the bone mineral, therefore slowing the amount of chemical reactions and substitutions that can take place [6, 47]. Impurities and other substitutions post-mortem can rapidly be incorporated into bone mineral before the crystallinity increases, however, due to the initial high sorption and reactivity of bioapatite [50, 51]. The timing and degree of recrystallization is related to the composition of the crystal structures, the rate of organic loss, and the depositional environment [10-12].

As the organic material protects the mineral phase and recrystallization is not instantaneous, the removal of collagen leaves bioapatite vulnerable to dissolution. There is no universally accepted, consistent, single solubility behavior of bioapatite, as bone is a complex biomaterial [51]. The causes of this variability have been ascribed to the particle size of bioapatite, the imperfections of the crystal lattice, and the presence of substitutions and calcium phosphate phases [51]. In the absence of universal thermodynamic model or bioapatite solubility, each crystal domain of bone mineral assigned its own Metastable Equilibrium Solubility (MES), which is a distribution phenomenon dependent on many variables and influenced by the size of the minerals and the extent of their surface reactivity [51]. Several aspects of bone quality such as carbonate content, ion vacancies, low crystallinity, and nanometric crystal dimensions have an effect on the MES phenomenon and predict likely vulnerabilities [31, 51].

Carbonate incorporation, typically through substitutions for hydroxyl or phosphate groups in the structure, increases the solubility of bioapatite [31, 51]. The substitutions and nonstoichiometry introduced through ion vacancies create imperfections in the crystal lattice,

which lower the crystalline order and introduce strain [31, 51]. This increases the dissolution tendency, therefore making the bioapatite with higher proportions of carbonate more susceptible to dissolution [32, 42, 51-53]. Apatite maturity, despite fluctuating values under the MES phenomenon, decreases solubility [31]. Changes in the highly reactive surface area either through shape or size in the plate-like bioapatite crystals can change the severity in susceptibility to diagenetic change.

Crystalline apatite and bioapatite are most soluble in acidic environments [12, 15, 54]. Bone exhibits low solubility and high survivorship in alkaline environments ($pH > 7.5$), except when such environments also have high carbon dioxide concentrations, which removes calcium ions and leaves bioapatite susceptible to demineralization [15].

Burnt Bone

Burning bone results in tremendous rapid changes to bioapatite crystal dimensions and structure, as well as the removal of organic components and incorporated water. These alterations are correlated to the degree of temperature intensity and burning atmosphere and result in products with different mechanical and structural properties depending on the extent of burning. These micro- and nano- scale transformations have a notable impact on visible macroscopic changes to heat altered bone, including: color changes, cracking, shrinkage, weight loss, and fragmentation [17, 28, 55, 56-58]. Processes such as manganese staining and sun bleaching may also influence bone color and to avoid the misidentification of burnt bone, changes in bone mineral crystal size, morphology and microstructure have been used to verify thermal alteration [21, 29, 49, 57, 58].

Four stages of burning have been defined that are correlated to the incineration and eventual removal of organics and transformation of bone mineral: dehydration, decomposition, inversion, and fusion [21, 57, 58, 60, 61]. These stages are accomplished at different temperature thresholds and were defined in oxidizing burning conditions [21, 57, 58, 60, 61]. The rate and degree of temperature induced changes depend on variables such as flesh coverage, heating and cooling rates and oxygen availability [57, 58]. Dehydration, or, the loss of water, occurs between 100 and 600˚C [21, 29, 57, 60, 61]. This wide temperature range likely accounts for the quicker loss of the loosely bound water between 25 and 250˚C and the eventual loss of the additional water more structurally bound to the mineral above 100˚C [21, 32, 60, 61].

After initial dehydration, the second stage of bone combustion is organic decomposition, from 300 to 800˚C [21, 60, 61]. With collagen degradation starting at 112 - 260˚C, above 300˚C a large proportion of the organics is reduced to a char [29]. Between 300 and 500˚C most mass, 50 – 55 %, is lost, and above 500˚C any remaining char is removed by 700˚C [29]. The macroscopic transformation most noticeable with the decomposition stage are the striking changes in coloring, with bone becoming visibly blackened with the charring of organics (300˚C) and after the complete removal of organics (700˚C) transitioning to a grey and chalk white hue [17, 21, 57, 60, 61]. Bone that is blackened is referred to as combusted or carbonized, while grey and white bone with all organics removed can be referenced as calcined [17, 57,58].

Simultaneous to the loss of organics is the alteration of the bioapatite mineral, or the inversion stage, between 500 and 1100˚C [21, 60, 61]. With the removal of the organic component at 300˚C, the larger, plate-like crystals can spontaneously grow at the expense of smaller crystals [21, 29, 60, 61]. Experiments with bone burnt while powdered and subsequently cleaned with acetone report mean crystallite size increasing to 10 - 30 nm, and crystallite

thickness moving from 2 to 9 nm [29, 60, 62]. Above 500˚C, additional growth has been observed, with reported crystallite sizes plateauing at 110 nm, with crystal thickness reaching 10 nm [29]. The hexagonal crystals later become equiaxed at 900˚C, growing more spheroidal, with overall dimensions reaching 300 - 550 nm [62].

The last stage of heat alteration to bioapatite, fusion, accounts for the microstructural changes noted with the inversion phase above 700˚C [21, 60, 61]. Bone porosity initially increases from *in vivo* status with the loss and charring of organics (~300˚C), corresponding to a loss in bone density [42, 62]. This carbonized bone is reported to be most porous right before temperatures of calcination (600˚C) [42]. Beginning at 700˚C there is a densification as the bioapatite crystal grains grow, and by 900˚C there is a total structural coalescence resulting in an interlocking structure and a marked decrease in porosity [21, 29, 62].

These changes are all products of burning in oxidizing conditions [58]. If a bone is brought to temperatures greater than 300˚C without access to oxygen, a different pattern of thermal alteration has been demonstrated in controlled experiments [58]. When heating occurs in reducing atmosphere, the organic char is not removed and instead becomes more ordered [58]. The crystallinity of the bioapatite does increase, however, although at a slower rate than indicated in oxidizing conditions [58]. New compounds, such as cyanamide, are also likely formed around 600˚-700˚C [58]. Bones burnt in reducing atmospheres above 600˚C do not lose the organic char component, and therefore remain black in coloring [58].

While extensive work has contributed to characterizing the heat induced transformations to the mineral and organic components of bone, it is still unclear how the timing of these changes can influence differentially burnt bone in an archaeological context. The evaluation of diagenetic

processes and taphonomic biases within assemblages of burnt fauna burnt to different temperatures is therefore necessary.

The site of Tolbor-17

The Ikh-Tolborin-Gol is a low altitude pass on the western flank of the Khangai Mountains of Northern Mongolia. It is part of the Selenga drainage system, the main river feeding Lake Baikal (Fig. 1) [64]. This river valley preserves a wealth of Upper Paleolithic (UP) locales including Tolbor-4, Tolbor-15, Tolbor-16, and Tolbor-17 (T-17) [64-67]. Most of the sites document periodic human occupations starting with the Initial Upper Paleolithic, ca. 45 ka, until the Holocene. The latter has recently been dated with polymineral post-IR IRSL, Quartz OSL, and radiocarbon to 42.5-45.6 ka, establishing the timing for a movement of population between the Siberian Altai and Northwestern China, contemporaneous with the earliest *Homo sapiens* fossils in the region [64]. The following occupations in the valley are associated with the Upper Paleolithic (UP) in the broad sense and were made by *Homo sapiens*. Although it is often assumed that fire is part of the modern human behavioral repertoire allowing expansion into cold climates, evidence of the use of fire in the UP Tolbor locales is rare and has been only briefly reported [68, 69].

Fig 1: Map of Mongolia with position of Tolbor-17 (modified after Geo-atlas).

Tolbor-17 provides a rare opportunity to investigate burning within faunal remains that are usually poorly preserved in the region. Like most of the other locales, T-17 is an open-air environment with a fluctuating low energy run-off constituting a recharge water regime. Initially

excavated as a series of two test pits with dimensions 2m x 1m, the excavators at T-17 piece plotted all finds > 2 cm, and the remaining sediment from each bucket volume of excavated material was dry sieved with 4mm and 2 mm mesh screens, with all material subsequently sorted. The T-17 lithological Unit 3 is characterized by the presence of UP lithic artifacts and organic faunal preservation, despite sedimentary evidence for episodic sheet erosion, prolonged groundwater interaction, chemical weathering, and long surface exposure. Based on its geological setting, the material studied here belongs to the second half of the Marine Isotope Stage (MIS) 3, ca. 40 -30 ka cal. BP. Unburnt and burnt fauna have been successfully recovered from Unit 3; however, this assemblage has been identified as extremely fragmentary and traditional zooarchaeological analyses considering butchery and taxon identification are still in the preliminary stages.

The inclusion of the faunal material from T-17 in this study is both for the purposes of providing a prehistoric archaeological assemblage for spectroscopic reference, as well as to specifically address the potential biases within this burnt portion of this faunal assemblage.

Materials and Methods:

Samples of archeological bones were characterized using Fourier transform infrared spectroscopy (FTIR) with an attenuated total reflectance (ATR) attachment, powder X-ray diffraction (XRD), and environmental scanning electron microscopy (eSEM). Burnt modern animal bone samples were annealed in a thermal analyzer to different temperatures and characterized using the same techniques to provide a controlled reference.

Archaeological bone sample collection and preparation

Mapped (> 2 cm) and screened (< 2 cm-2 mm) faunal remains from the T17 UP assemblage were cleaned, weighed in milligrams, and sorted following the Stiner et al. [17] seven stage visual scale of burning intensity (Table 2). No burning was noted in bone > 2 cm except for a single fragment, but fauna < 2 cm- 2mm recovered in the screened material is found to span all stages of burning intensity within Unit 3 of the exposed excavation surface (Table 2). Of the burnt fauna, a large percentage is nearly or fully calcined, a notable observation due to the described mechanical fragility of calcined bone and unprotected open-air environment. 20 bones from the Unit 3 assemblage, representing each burning category and from the same meter square, were sampled for subsequent spectroscopic analyses to confirm heat alteration and investigate crystallite size. Bones were cleaned with ionic water sonication, and bone powder was produced using a diamond file and an agate mortar and pestle. All archaeological bone powder samples were sieved with 234 μm mesh.

Table 2: T17 Unit 3 fauna summary with burning stages following Stiner et al.[17].

	Screened (< 2 cm)	Piece plotted (>2 cm)	Total
Burning Stage	Weight (mg)	Weight (mg)	Weight (mg)
0	141.36	213.31	354.47
1	1.04	0	1.04
2	5.17	1.01	6.18
3	0.69	0	0.69
4	1.95	0	1.95
5	1.37	0	1.37
6	4.5	0	4.5

Modern bone sample collection and preparation

A controlled experimental reference collection was created with modern bone to investigate the impact of thermal alteration on crystallite size and loss of organics. Cortical bone

from three cow femurs and two horse metacarpals from five different individuals were selected for this study. Cow femurs were procured the day after butchery from a local butcher and were never frozen. Flesh was scraped manually to prepare for drilling. Horse metacarpals were obtained postmortem from completed forelimb tissue collections of horses humanely euthanized for purposes other than this study at the UC Davis School of Veterinary Medicine. Metacarpals were simmered in water with the addition of borax to assist with defleshing, although bones remained greasy.

A diamond drill coring bit was used to produce solid plugs of cortical bone 3mm x 3mm, with weights ranging from 53.2 to 58.1 mg. Coring was constrained to the cortical bone tissue from the mid-diaphysis of both cow and horse bones. Solid bone plugs were specifically utilized in lieu of bone powder to avoid the effects of powder heating, as powder has an increased surface area and would be more reactive to thermal alteration. Subsequent to drilling, plugs were filed with a diamond file to fit the dimensions of crucibles used for thermal analysis

Thermal analysis of modern bone samples

The controlled annealing of modern bone samples was performed with Setaram Labsys Evo thermal analyzer. Bone core samples were placed in a 100 µl Al_2O_3 crucible and air flow 40 ml/min was established. The samples were brought to desired temperatures in 100 -1200°C range with heating rate 20˚C /min and held isothermally for 30 minutes. The weight change and heat flow traces were recorded continuously and corrected for the baseline. Additional samples were produced at 300 and 700˚C with ramp 50°C /min and one hour dwell time for comparison. Post heating, bone plug samples were powdered with an agate mortar and pestle and sieved with 234 µm mesh.

Infrared spectroscopy data collection and analysis

Infrared spectroscopy can yield information regarding the relative degree of structural order, size, and strain of bioapatite crystals through the measurement of spectral peaks associated primarily with the different vibrational frequencies of bone carbonate and phosphate. This is relevant for the study of the thermal alteration of bone, as it can verify changes associated with burning that cannot be mistaken for macroscopic staining or bleaching. These changes include the increase of crystallinity that accompanies burning as bone minerals become more ordered, as larger crystals grow at the expense of smaller crystals, and as the organic components are eventually lost [70, 71].

A Nicolet 6700 Fourier transform infrared spectrometer with an ATR attachment and a deuterated triglycine sulfate (DTGS) detector and single bounce diamond crystal was used. The ATR method uses an attachment with a diamond or zinc crystal to produce spectra through the phenomenon of internal reflectance [72, 73]. The application of ATR minimizes sample preparation, which in turn minimizes contamination [49, 70, 71]. Spectra were collected with 256 scans in the 4000 - 400 cm^{-1} frequency region and with an 8 mm spectral range. Each archaeological and modern bone powder sample was retested for quality control.

The identification of FTIR spectral peaks associated with bone has been extensively documented (75). Relevant peaks to this study and their inferred functional groups include the 1650 cm^{-1} and 1550 cm^{-1} peaks for the measurement of amide I and II, the 874 cm^{-1} and 1415 cm^{-1} peaks indicating presence of the v2 and v3 of carbonate, and the 900-1200 cm^{-1} and 50-600 cm^{-1} spectral regions related to the v3 and v4 phosphate components (Table 3). Additionally, the

appearance of a 625cm^{-1} shoulder peak is attributed here to PO_4^{3-} v4 bending, known as the phosphate high temperature (PHT).

FTIR-ATR spectra were processed with OMNIC software to verify heat alteration and measure the relative degree of crystallinity. Thermal alteration can be monitored in bioapatite through: (1) the ratio of carbonate to phosphate present in the sample, the C/P ratio, (2) the depletion of the presence of amide I and II functional groups, representing the organic components of bone, and (3) the presence of heat specific peak splitting, such as the loss of the peak at 874 cm^{-1} correlated to CO_3^{2-} v2 at temperatures over 1000˚C, and the PHT shoulder peak at temperatures over 700˚C [57, 58, 61]. Measures of the crystallinity of a sample can be inferred from the infrared splitting factor (IRSF), which extrapolates the changing size and order of bioapatite crystals through increase of splitting seen in the PO_4^{3-} v4 peaks [57, 61, 74]. Eight peak measurements were monitored for 168 scans representing 84 individual samples for this study, 62 modern and 22 archaeological. Each sample was tested twice, and measurements presented here represent the average values of both scans.

Table 3: FTIR-ATR wavenumbers associated with likely functional groups relevant to this study and the thermal alteration of bone.

Wavenumber	Inferred peak assignment	Peak transformation relevant to this study
1630-1660 cm $^{-1}$	organic tissue and water, amide I + II	decrease and absence
1400-1550 cm $^{-1}$	CO_3^{2-} v3	1415 cm^{-1} peak a component of C/P ratio
1028-1100 cm^{-1}	PO_4^{3-} v3	1035 cm $^{-1}$ peak a component of the C/P ratio
874 cm^{-1}	CO_3^{2-} v2	peak loss
565 cm^{-1}, 605 cm^{-1}	PO_4^{3-} v4	growth of 565 cm^{-1} and 605 cm^{-1} and decrease of the 595 cm^{-1} trough utilized for the infrared splitting factor (IRSF); phosphate

high temperature (PHT) shoulder growth at 625 cm^{-1}

The IRSF measurements were procured for all samples following Weiner and Bar-Yosef [74].

$$\text{Infrared Splittng Factor:} \frac{(565\ \text{cm}^{-1}\ peak\ ht + 605\ \text{cm}^{-1}\ peak\ ht)}{595\ \text{cm}^{-1}\ \ peak\ ht}$$

An additional measure of the carbonate to phosphate content, the C/P ratio, was also determined for all samples. The C/P ratio decreases with burning and utilizes the 1035 cm^{-1} phosphate peak unaffected by IRSF changes [49, 71].

$$\frac{\text{C}}{\text{P}}\ \text{ratio:} \frac{1415\ \text{cm}^{-1}\ peak\ ht}{1035\ \text{cm}^{-1}\ peak\ ht}$$

Other peaks observed for this analysis were noted as they are related to the loss of organics and specific heat-induced changes [57, 58, 61] (Table 3).

X-ray diffraction analysis

X-ray Diffraction (XRD) can be used to measure the relative sizes of bioapatite crystals [29, 42, 57, 62]. Powder XRD patterns here were obtained using Bruker D2 Phaser and Bruker D8 advance diffractometers using CuKα radiation. Bone powder samples were taken from solid

bone plugs and spread with ethanol on a zero background silicon sample holder, and run from 10 to 90 °2θ with 0.02° step. Dwell time was chosen to obtain at least thousand counts on the most intense peaks. The average crystallite size of analyzed samples was obtained from diffraction peaks broadening using whole pattern fitting (Rietveld refinement) procedure as implemented in Jade MDI software [76]. Diffraction profile was modeled using hydroxyapatite $Ca_5(PO_4)_3OH$ structure (space group P63/m) and pseudo-Voigt profile shape function. The instrumental broadening was accounted for by calibration with NIST LaB_6 profile shape standard. The uncertainties in crystallite sizes are reported as obtained from least squares refinement.

Scanning Electron Microscopy

Secondary electron (SE) images were acquired for two samples of archaeological bone and three samples of modern bone annealed at different temperatures (300, 700, and 1200˚C) in a Quattro environmental scanning electron microscope (eSEM), manufactured by ThermoFischer Scientific. The SE images were obtained at an accelerating voltage of either 10kV or 20kV and with an electron beam width, or size spot, of 3.0. Spot 3 is commonly used to attain sufficient signal without compromising resolution. To prevent the buildup of charge on sample surfaces, the SE images were acquired in low vacuum mode with partial pressure of water set at 400 Pa. A low vacuum detector (LVD), which is optimized for this pressure range, was used to measure the SE image signal.

Results:

FTIR-ATR of modern bone

The FTIR-ATR analyses were run on each modern sample for the verification of heat alteration and the determination of the size and relative order of the bioapatite crystals at each temperature threshold. All modern samples were found to exhibit spectra indicative of the thermal alteration of bone in oxygen atmospheres supported by previous research (Fig 2, 3), including the decrease of C/P ratio, decrease of organic components by 300˚C with complete absence seen by 400˚C, the absence the 874 cm^{-1} peak above 1000˚C, and the presence of the PHT peak splitting above 700˚C (S1 Table, S2 Table). No differences were indicated in the reheated or increased rate samples taken to 300 and 700˚C from the single-heated or controlled rate counterparts.

Fig. 2: FTIR-ATR spectra of experimentally modern burnt samples grouped by with functional groups highlighted in the range of 1700-800 cm^{-1}.

Fig. 3: FTIR-ATR spectra of experimentally modern burnt samples with functional groups highlighted in the range of 700-500 cm^{-1}.

The IRSF of all modern samples also followed reported trends in bioapatite crystallinity, with order, size and strain increasing alongside intensifying temperatures (Fig. 2, 3, 4; S1 Table, S2 Table) [57, 60]. This increase in crystallinity is seen until 1000˚C, after which there is a marked decrease in IRSF coinciding with the equiaxing of bioapatite crystals (Fig. 4). Despite the general acceptance of the IRSF and the application of this ratio to study bone quality and thermal alteration in bone, the values of IRSF can have large variations despite consensus on general trends [57, 60]. This is seen most dramatically in the range of IRSF values reported for

all samples at 900˚C in this study (Fig. 4; S2 Table). No changes in IRSF were noted in samples reheated or heated with increased rates.

Fig. 4: Infrared Splitting Factor (IRSF) of the experimental modern and archaeological collection measured from FTIR-ATR spectra following Weiner and Bar Yosef [74].

FTIR-ATR of archaeological bone

The FTIR-ATR spectra produced from the T-17 archaeological collection, organized by the Stiner et al. [17] stage of burning intensity, supports the macroscopic indication of heat alteration and no intrusive staining or bleaching (Fig. 5, 6). Good agreement is seen with the relative decreases of C/P ratio and the loss of organic components by Stage 3 (fully carbonized) between the archaeological and modern samples (Fig. 5, 6; S3 Table, S4 Table). The appearance of the PHT with bones identified as Stage 5 supports the presence of temperatures above 700˚C at T-17, although the continued presence of CO_3^{2-} v2 inferred by the 874 cm^{-1} peak indicates temperatures likely did not reach above 1000˚C (Fig. 5, 6; S3 Table).

Fig. 5: FTIR-ATR spectra of archaeological fauna from T-17 grouped by stage of burning intensity following Stiner et al. [17] with functional groups highlighted in the range of 1700-800 cm^{-1}.

Fig. 6: FTIR-ATR spectra of archaeological fauna from T-17 grouped by stage of burning intensity following Stiner et al. [17] with functional groups highlighted in the range of 700-500 cm^{-1}.

The IRSF of the T-17 samples also follows the trends of the experimental modern collection, with gradual increases seen through Stage 3 and higher values reported with the presence of calcination with Stages 4, 5, and 6 (Fig. 4, S3 Table, S4 Table). Elevated values are not seen within the Stage 0 unburnt samples of T-17 bone, demonstrating crystallinity values were not heightened due to diagenesis [17]. As expected, the IRSF values can distinguish between calcined and non-calcined samples, but cannot distinguish between low temperature burning samples.

XRD of modern bone

The results of the XRD analyses on the modern collection demonstrate the increasing crystallite size correlated to temperature, specifically above temperatures of calcination (700˚C) (Fig. 7; S2 Table). An average size threshold is clearly noted, with all samples unburnt through 600˚C averaging 9 nm, while all samples burnt at 700˚C jump to an average of 41 nm (Fig. 7). An additional increase in crystallite size by approximately 30 nm is noted at 900˚C, coinciding with the fusion stage of thermal alteration of bone, with samples reaching an average of 72 nm (Fig. 7).

Fig. 7: XRD results of crystallite size (nm) alongside selected eSEM images of experimental modern (A-C) and archaeological (D-E) samples highlighting the changes to crystal shape with heat alteration.

XRD of archaeological bone

The average crystallite size measurements for the archaeological T-17 samples confirm the presence of calcination within samples at Stages 4, 5, and 6 with considerable crystal growth (Fig. 7; S4 Table). The XRD results also demonstrate that within Stage 6 temperature thresholds can be distinguished, with two of the Stage 6 samples reaching crystallite sizes that group with the ≥ 900˚C experimental group (Fig. 7). These crystal changes, including the transformation of plate-like crystals to larger equiaxed morphologies, are depicted clearly with eSEM images taken of both collections (Fig. 7).

The average crystallite growth noted in this study provides a pathway for distinguishing between temperatures within the macroscopic identification of Stage 6 burning based on color. The results presented here provide a metric to speak to two categories of burning within Stage 6: >700˚C and >900˚C.

Discussion:

The FTIR-ATR and XRD results in the modern and archaeological collection address timing of organic loss, changes in crystallinity, and crystallite size growth with increases in temperature intensity in oxygen atmospheres. The modern component of this study clearly demonstrates the initial loss of organics with carbonization and a massive crystal growth threshold that accompanies calcination by 700˚C. These modifications have large implications for the susceptibility of bone burnt to different temperatures to dissolution, especially when considered alongside the other factors inherent in bone diagenesis: organic protection and porosity.

Unburnt bone has small, highly reactive crystals but is overall less friable than burnt bone and is more likely to not be highly fragmented. Larger sizes, along with an intact insoluble

organic component, protects the bone mineral from hydrolysis and microbial attack. Carbonized bone also has small, highly reactive crystals, but is more likely to be fragmented, as fragmentation is a function of burning intensity [17]. Bone burnt fully to temperatures of carbonization, 300˚C and Stiner et al. [17] Stage 3, also has the organic component eliminated resulting in the removal of protection for the bone mineral. This also results in a more open porosity which is not altered until very high temperatures, ~900˚C [61]. Carbonized and charred bone, therefore, is especially vulnerable to aqueous dissolution, as it is likely to be fragmented and small, has lost organic protection, has a large degree of porosity, and has very reactive small crystals. Bone calcined by 700˚C, starting at Stiner et al. [17] Stage 4 and completely calcined by Stiner et al. [17] Stage 6, is mechanically fragile, but the massive increase in crystal sizes reduces the surface to mass ratio (active surface area) of bioapatite. This results in minerals that are much less soluble. Bone which has been thermally altered above 900˚C, which can only be identified macroscopically as Stiner et al. [17] Stage 6, benefits further from a closing of the porosity, providing additional protection.

The measurements presented here regarding the loss of organics, crystallinity, and crystallite sizes are in consensus with trends reported by previous studies, with variance likely introduced by the burning of bulk versus powder samples and the impacts of the MES phenomenon, different depositional environments under consideration, and the variance in experimental protocols and burning regimes. Additionally, treatment of modern bone is often not reported, despite the tremendous effect freezing was found to exhibit on sample porosity [6].

Due to the immediate presence of low energy water movement and the effects of prolonged exposure in the open-air environment, it is likely that the T-17 UP burnt faunal assemblage from Unit 3 is biased and has an underrepresentation of carbonized bone. This

inference is in agreement with other studies of diagenesis, which have noted the large effects of water interaction on bone preservation [10, 11, 37, 44]. Despite the projected likelihood of bias in temperature distribution, the presence of fire at T-17 can be described as reaching likely temperatures of at least 900˚C, as evidenced by two samples having crystallite sizes grouping with the ≥ 900˚C threshold, but constrained by the continued presence of CO_3^{2-} v2 in two samples of Stage 6 FTIR-ATR spectra, which is lost at temperatures at and above 1000˚C. Future work at T-17 will investigate the microstratigraphy and association of these faunal remains to address the potential of this evidence for an in situ open air anthropogenic fire event.

Conclusion:

The aim of this study is to describe the timing of the heat induced changes to the organic and mineral components of bone, and the impact these alterations would have on burial environments with water interaction. The results are contextualized alongside an archaeological assemblage evaluated with standard zooarchaeological methods of burning identification. The importance of small bone fragments in the study of ancient fire is additionally highlighted, matching observations noted in previous studies [17, 77]. Excluding the screened faunal material, the T-17 fauna sample would have obscured the evidence for ancient fire, as nearly all burned bones except for one fragment were recovered in the < 2cm screened fraction.

Faunal remains are a valuable tool to describing fire characteristics and identifying combustion features, but bone burnt to different temperature thresholds will not be impacted uniformly by water dissolution due to the heat induced differences in crystallite size, the amount of organic components, and the degree of porosity. Studies engaging with fauna to describe ancient fires must recognize that bones burnt to lower temperatures are more vulnerable to

hydrolysis and may be greatly underrepresented, while bones which are heated to the point of calcination, though mechanically fragile, have greater protection against dissolution. This bias against bone burnt to lower temperatures has large implications on the identification of ancient fire from a faunal perspective: burnt osseous material resulting from fires that utilize fuel with low burning temperatures, such as grass, and in environments with greater water interaction may be less visible in the archaeological record [18].

S1 Table: Experimental modern bone FTIR-ATR relevant peak height values.
S2 Table: Calculated experimental modern sample C/P and IRSF from FTIR-ATR spectra, as well as raw Angstrom values as measured from XRD.
S3 Table: FTIR-ATR T-17 Unit 3 archaeological sample relevant peak height values. All samples recovered from same meter square.
S4 Table: Calculated archaeological T-17 Unit 3 C/P and IRSF values from FTIR-ATR spectra, as well as raw Angstrom data from XRD. All samples recovered from same meter square.

Acknowledgements:

The authors would like to thank Dr. Teresa Steele for her continued feedback and illuminating discussions, and Chris Gallo for his many helpful explanations. We additionally thank Drs. Eerkens, Darwent, and Parikh for their generosity and lab resources, as well as the patience, assistance, and expertise of the Peter A. Rock Thermochemistry Laboratory, NEAT research group, and the Parikh Environmental Soil Chemistry Group at the University California Davis. The authors also recognize the support given from the UC Davis Veterinary Medical Teaching Hospital, the UC Davis JD Wheat Veterinary Orthopedic Laboratory, the UC Davis AMCaT Laboratory, the UC Davis DHI Transdisciplinary Research Cluster “The Cluster for Archaeology and Soil Synergy”, the Prehistory department of Université Liège, and the Center for Experimental Archaeology at UC Davis (CEAD). The excavation at T-17 is supported by the NSF (PI Zwyns, Grant #156074) and the PaleoAsia Project (Izuho, Nishiaki).

References:

1. Binford LR, Bertram JB. Bone frequencies and attritional processes. In (LR Binford, Ed.) For Theory Building in Archaeology. 1977.
2. Lyman RL. Bone density and differential survivorship of fossil classes. Journal of Anthropological archaeology. 1984;3(4):259–99.
3. Grayson DK. Bone transport, bone destruction, and reverse utility curves. Journal of Archaeological Science. 1989 Nov 1;16(6):643–52.
4. Outram AK. A new approach to identifying bone marrow and grease exploitation: why the “indeterminate” fragments should not be ignored. Journal of archaeological science. 2001;28(4):401–10.
5. Munson PJ, Garniewicz RC. Age-mediated survivorship of ungulate mandibles and teeth in canid-ravaged faunal assemblages. Journal of Archaeological Science. 2003;30(4):405–16.
6. Robinson S, Nicholson RA, Pollard AM, O’Connor TP. An Evaluation of Nitrogen Porosimetry as a Technique for Predicting Taphonomic Durability in Animal Bone. Journal of Archaeological Science. 2003 Apr;30(4):391–403.
7. Von Endt DW, Ortner DJ. Experimental effects of bone size and temperature on bone diagenesis. Journal of Archaeological Science. 1984 May;11(3):247–53.
8. Lam YM, Chen X, Pearson OM. Intertaxonomic variability in patterns of bone density and the differential representation of bovid, cervid, and equid elements in the archaeological record. American Antiquity. 1999;64(2):343–62.
9. Lyman RL. Vertebrate taphonomy. Cambridge University Press; 1994.
10. Nielsen-Marsh CM, Hedges REM. Patterns of Diagenesis in Bone I: The Effects of Site Environments. Journal of Archaeological Science. 2000 Dec;27(12):1139–50.
11. Nielsen-Marsh CM, Smith CI, Jans MME, Nord A, Kars H, Collins MJ. Bone diagenesis in the European Holocene II: taphonomic and environmental considerations. Journal of Archaeological Science. 2007 Sep;34(9):1523–31.
12. Nielsen-Marsh C, Gernaey A, Turner-Walker G, Hedges R, Pike A, Collins M. The chemical degradation of bone. Human Osteology in archaeology and forensic science. 2000: 439-454.
13. Denys C. Taphonomy and experimentation. Archaeometry. 2002 Aug;44(3):469–84.
14. Maurer A-F, Person A, Tütken T, Amblard-Pison S, Ségalen L. Bone diagenesis in arid environments: An intra-skeletal approach. Palaeogeography, Palaeoclimatology, Palaeoecology. 2014 Dec;416:17–29.
15. Turner-Walker G. The Chemical and Microbial Degradation of Bones and Teeth. In: Pinhasi R, Mays S, editors. Advances in Human Palaeopathology [Internet]. Chichester, UK: John Wiley & Sons, Ltd; 2007:3–29.
16. Costamagno S, Théry-Parisot I, Brugal J-P, Guibert R. Taphonomic consequences of the use of bones as fuel. Experimental data and archaeological applications. In: Biosphere to lithosphere: new studies in vertebrate taphonomy. Oxbow Books Oxford; 2005. p. 51–62.
17. Stiner MC, Kuhn SL, Weiner S, Bar-Yosef O. Differential Burning, Recrystallization, and Fragmentation of Archaeological Bone. Journal of Archaeological Science. 1995 Mar;22(2):223–37.
18. Berna F, Goldberg P, Horwitz LK, Brink J, Holt S, Bamford M, et al. Microstratigraphic evidence of in situ fire in the Acheulean strata of Wonderwerk Cave, Northern Cape

province, South Africa. Proceedings of the National Academy of Sciences. 2012 May 15;109(20):E1215–20.
19. Goldberg P, Dibble H, Berna F, Sandgathe D, McPherron SJP, Turq A. New evidence on Neandertal use of fire: Examples from Roc de Marsal and Pech de l'Azé IV. Quaternary International. 2012 Jan;247:325–40.
20. Barkai R, Rosell J, Blasco R, Gopher A. Fire for a reason: Barbecue at middle Pleistocene Qesem cave, Israel. Current Anthropology. 2017 Aug 1;58(S16):S314-28.
21. Thompson T. Recent advances in the study of burned bone and their implications for forensic anthropology. Forensic Science International. 2004;146:S203–5.
22. Ubelaker DH. The forensic evaluation of burned skeletal remains: A synthesis. Forensic Science International. 2009 Jan;183(1–3):1–5.
23. Morin E. Taphonomic implications of the use of bone as fuel. Palethnologie. 2010;2:209-17.
24. Théry-Parisot I. Fuel management (bone and wood) during the Lower Aurignacian in the Pataud rock shelter (Lower Palaeolithic, Les Eyzies de Tayac, Dordogne, France). Contribution of experimentation. Journal of Archaeological Science. 2002;29(12):1415–21.
25. Schiegl S, Goldberg P, Pfretzschner H-U, Conard NJ. Paleolithic burnt bone horizons from the Swabian Jura: Distinguishing between in situ fireplaces and dumping areas. Geoarchaeology. 2003;18(5):541–65.
26. Speth J, Clark J. Hunting and overhunting in the Levantine Late Middle Palaeolithic. Before Farming. 2006 Jan;2006(3):1–42.
27. Costamagno S, Théry-Parisot I, Castel JC, Brugal JP. Combustible ou non? Analyse multifactorielle et modèles explicatifs sur des ossements brûlés paléolithiques. *Geston des combustibles au Paléolithique et au Mésolithique: nouveaux outils, nouvelles interpretations*. 2009:61
28. Buikstra JE, Swegle M. Bone modification due to burning: experimental evidence. Bone modification. 1989;247–58.
29. Etok SE, Valsami-Jones E, Wess TJ, Hiller JC, Maxwell CA, Rogers KD, et al. Structural and chemical changes of thermally treated bone apatite. J Mater Sci. 2007 Sep 21;42(23):9807–16.
30. Martin RB, Burr DB, Sharkey NA, Fyhrie DP. Skeletal tissue mechanics. Second edition. New York: Springer; 2015. 762 p.
31. Rollin-Martinet S, Navrotsky A, Champion E, Grossin D, Drouet C. Thermodynamic basis for evolution of apatite in calcified tissues. American Mineralogist. 2013 Nov 1;98(11–12):2037–45.
32. Drouet C, Aufray M, Rollin-Martinet S, Vandecandelaère N, Grossin D, Rossignol F, et al. Nanocrystalline apatites: The fundamental role of water. American Mineralogist. 2018 Apr 1;103(4):550–64.
33. Stout SD, Cole ME, Agnew AM. Histomorphology. In: Ortner's Identification of Pathological Conditions in Human Skeletal Remains [Internet]. Elsevier; 2019 [cited 2019 Oct 20]. p. 91–167.
34. Rey C, Combes C, Drouet C, Glimcher MJ. Bone mineral: update on chemical composition and structure. Osteoporos Int. 2009 Jun;20(6):1013–21.

35. Greiner M, Rodríguez-Navarro A, Heinig MF, Mayer K, Kocsis B, Göhring A, et al. Bone incineration: An experimental study on mineral structure, colour and crystalline state. Journal of Archaeological Science: Reports. 2019;25:507–18.
36. Bala Y, Farlay D, Boivin G. Bone mineralization: from tissue to crystal in normal and pathological contexts. Osteoporos Int. 2013 Aug;24(8):2153–66..
37. Berna F, Matthews A, Weiner S. Solubilities of bone mineral from archaeological sites: the recrystallization window. Journal of archaeological Science. 2004 Jul 1;31(7):867-82.
38. Nyman JS, Ni Q, Nicolella DP, Wang X. Measurements of mobile and bound water by nuclear magnetic resonance correlate with mechanical properties of bone. Bone. 2008;42(1):193–9.
39. Trueman CN, Privat K, Field J. Why do crystallinity values fail to predict the extent of diagenetic alteration of bone mineral? Palaeogeography, Palaeoclimatology, Palaeoecology. 2008 Sep;266(3–4):160–7.
40. Koeppenkastrop D, Eric H. Sorption of rare-earth elements from seawater onto synthetic mineral particles: An experimental approach. Chemical geology. 1992;95(3–4):251–63.
41. Reynard B, Lécuyer C, Grandjean P. Crystal-chemical controls on rare-earth element concentrations in fossil biogenic apatites and implications for paleoenvironmental reconstructions. Chemical Geology. 1999;155(3–4):233–41.
42. Figueiredo M, Fernando A, Martins G, Freitas J, Judas F, Figueiredo H. Effect of the calcination temperature on the composition and microstructure of hydroxyapatite derived from human and animal bone. Ceramics International. 2010 Dec;36(8):2383–93.
43. Tripp JA, Squire ME, Hedges REM, Stevens RE. Use of micro-computed tomography imaging and porosity measurements as indicators of collagen preservation in archaeological bone. Palaeogeography, Palaeoclimatology, Palaeoecology. 2018 Dec;511:462–71.
44. Smith CI, Nielsen-Marsh CM, Jans MME, Collins MJ. Bone diagenesis in the European Holocene I: patterns and mechanisms. Journal of Archaeological Science. 2007;34(9):1485–93.
45. Hedges REM, Millard AR. Bones and Groundwater: Towards the Modelling of Diagenetic Processes. Journal of Archaeological Science. 1995 Mar;22(2):155–64.
46. Hedges REM. Bone diagenesis: an overview of processes. Archaeometry. 2002 Aug;44(3):319–28.
47. Pfretzschner H-U. Fossilization of Haversian bone in aquatic environments. Comptes Rendus Palevol. 2004 Oct;3(6–7):605–16.
48. Tütken T, Vennemann TW, Pfretzschner H-U. Early diagenesis of bone and tooth apatite in fluvial and marine settings: Constraints from combined oxygen isotope, nitrogen and REE analysis. Palaeogeography, Palaeoclimatology, Palaeoecology. 2008 Sep;266(3–4):254–68.
49. Beasley MM, Bartelink EJ, Taylor L, Miller RM. Comparison of transmission FTIR, ATR, and DRIFT spectra: implications for assessment of bone bioapatite diagenesis. Journal of Archaeological Science. 2014 Jun;46:16–22.
50. Trueman CN, Behrensmeyer AK, Potts R, Tuross N. High-resolution records of location and stratigraphic provenance from the rare earth element composition of fossil bones. Geochimica et Cosmochimica Acta. 2006 Sep;70(17):4343–55.

51. Baig AA. Influences of carbonate content and crystallinity on the solubility behavior of synthetic and biological apatites. Department of Pharmaceutics and Pharmaceutical Chemistry, University of Utah; 1997.
52. Baig AA, Fox JL, Wang Z, Higuchi WI, Miller SC, Barry AM, et al. Metastable Equilibrium Solubility Behavior of Bone Mineral. Calcified Tissue International. 1999 Apr 1;64(4):329–39.
53. LeGeros RZ, Bonel G, Legros R. Types of "H 2 O" in human enamel and in precipitated apatites, Calcified Tissue Research. 1978 Dec 1; 26(1):111-8.
54. White EM, Hannus LA. Chemical Weathering of Bone in Archaeological Soils. American Antiquity. 1983;48(2):316–22.
55. Shipman P, Foster G, Schoeninger M. Burnt bones and teeth: an experimental study of color, morphology, crystal structure and shrinkage. Journal of Archaeological Science. 1984 Jul;11(4):307–25.
56. Thompson T, Ulgium, P. Burned Human Remains. In Handbook of Forensic Anthropology and Archaeology. Routledge. 2016: 295-303.
57. Ellingham STD, Thompson TJU, Islam M, Taylor G. Estimating temperature exposure of burnt bone — A methodological review. Science & Justice. 2015 May;55(3):181–8.
58. Reidsma FH, van Hoesel A, van Os BJH, Megens L, Braadbaart F. Charred bone: Physical and chemical changes during laboratory simulated heating under reducing conditions and its relevance for the study of fire use in archaeology. Journal of Archaeological Science: Reports. 2016 Dec;10:282–92.
59. Shahack-Gross R, Bar-Yosef O, Weiner S. Black-coloured bones in Hayonim Cave, Israel: differentiating between burning and oxide staining. Journal of archaeological Science. 1997 May 1;24(5):439-46.
60. Thompson TJU. The Analysis of Heat-Induced Crystallinity Change in Bone. In: The Analysis of Burned Human Remains. Elsevier; 2015. p. 323–37.
61. Thompson TJU, Islam M, Bonniere M. A new statistical approach for determining the crystallinity of heat-altered bone mineral from FTIR spectra. Journal of Archaeological Science. 2013 Jan;40(1):416–22.
62. Pramanik S, Hanif A, Pingguan-Murphy B, Abu Osman N. Morphological Change of Heat Treated Bovine Bone: A Comparative Study. Materials. 2013. Dec 21;6(1):65–75.
63. Ubelaker DH, Rife JL. The practice of cremation in the Roman-era cemetery at Kenchreai, Greece. Bioarchaeology of the Near East. 2007;1:35-57.
64. Zwyns N, Paine CH, Tsedendorj B, Talamo S, Fitzsimmons KE, Gantumur A, et al. The Northern Route for Human dispersal in Central and Northeast Asia: New evidence from the site of Tolbor-16, Mongolia. Sci Rep. 2019 Dec;9(1):11759.
65. Zwyns N, Gladyshev SA, Gunchinsuren B, Bolorbat T, Flas D, Dogandžić T, et al. The open-air site of Tolbor 16 (Northern Mongolia): Preliminary results and perspectives. Quaternary International. 2014 Oct;347:53–65.
66. Gladyshev SA, Olsen JW, Tabarev AV, Jull AJT. The Upper Paleolithic of Mongolia: Recent finds and new perspectives. Quaternary International. 2012 Dec;281:36–46.
67. Tabarev A., Gunchinsuren .B, Gillam J., Gladyshev S., Dogandžić T., Zwyns N. Kompleks Pamiatnikov Kamennogo Veka v Doline r.Ikh Tulberiin-Gol, Severnaya Mongolia (razvedochnye raboty s ispolzovaniem GIS-Technnologii v. 2011 g) [The Stone Age site complex in the Ikh-Tulberiin-Gol River Valley, North Mongolia (survey

and research using GIS-technology in 2011)]. Studia Archaeologica Instituti Archaeologici Academiae Scientiarum Mongolicae. 2012;32:26–43
68. Gowlett JAJ. The early settlement of northern Europe: Fire history in the context of climate change and the social brain. Comptes Rendus Palevol. 2006 Jan;5(1–2):299–310.
69. Rybin EP, Khatsenovich AM, Gunchinsuren B, Olsen JW, Zwyns N. The impact of the LGM on the development of the Upper Paleolithic in Mongolia. Quaternary International. 2016 Dec;425:69–87.
70. Hollund HI, Ariese F, Fernandes R, Jans MME, Kars H. Testing an alternative high-throughput tool for investigating bone diagenesis: FTIR in attenuated total reflection (ATR) mode: An alternative tool for investigating bone diagenesis. Archaeometry. 2013 Jun;55(3):507–32.
71. Thompson TJU, Gauthier M, Islam M. The application of a new method of Fourier Transform Infrared Spectroscopy to the analysis of burned bone. Journal of Archaeological Science. 2009 Mar;36(3):910–4.
72. Nakamoto K. Infrared and Raman Spectra of Inorganic and Coordination Compounds (Part A: Theory and Applications in Inorganic Chemistry)(Volume 1A)(Part B: Applications in Coordination, Organometallic, and Bioinorganic Chemistry)(Volume 1B). NY, John Wiley & Sons, Incorporated; 1997.
73. Bruno TJ. Sampling Accessories for Infrared Spectrometry. Applied Spectroscopy Reviews. 1999 Jul 20;34(1–2):91–120.
74. Weiner S, Bar-Yosef O. States of preservation of bones from prehistoric sites in the Near East: A survey. Journal of Archaeological Science. 1990 Mar;17(2):187–96.
75. Paschalis EP, DiCarlo E, Betts F, Sherman P, Mendelsohn R, Boskey AL. FTIR microspectroscopic analysis of human osteonal bone. Calcified tissue international. 1996 Dec 1;59(6):480-7.
76. Rietveld H. A profile refinement method for nuclear and magnetic structures. Journal of applied Crystallography. 1969 Jun 2;2(2):65-71.
77. Villa P, Castel JC, Beauval C, Bourdillat V, Goldberg P. Human and carnivore sites in the European Middle and Upper Paleolithic: similarities and differences in bone modification and fragmentation. Revue de paléobiologie. 2004;23(2):705-30.

Fig 1

Click here to access/download;Figure;Fig1.tif

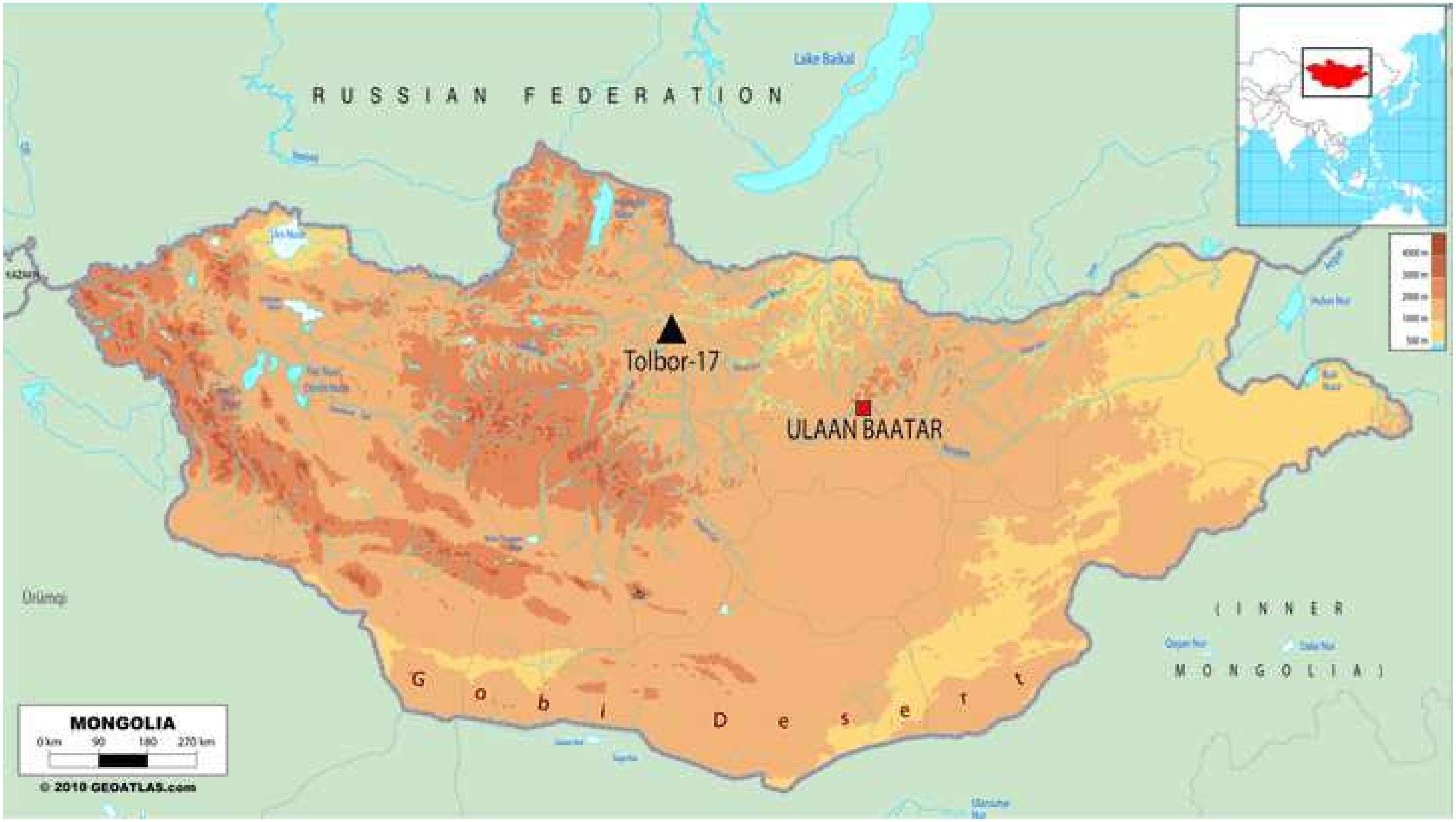

Fig 2

Click here to access/download;Figure;Fig2.tif

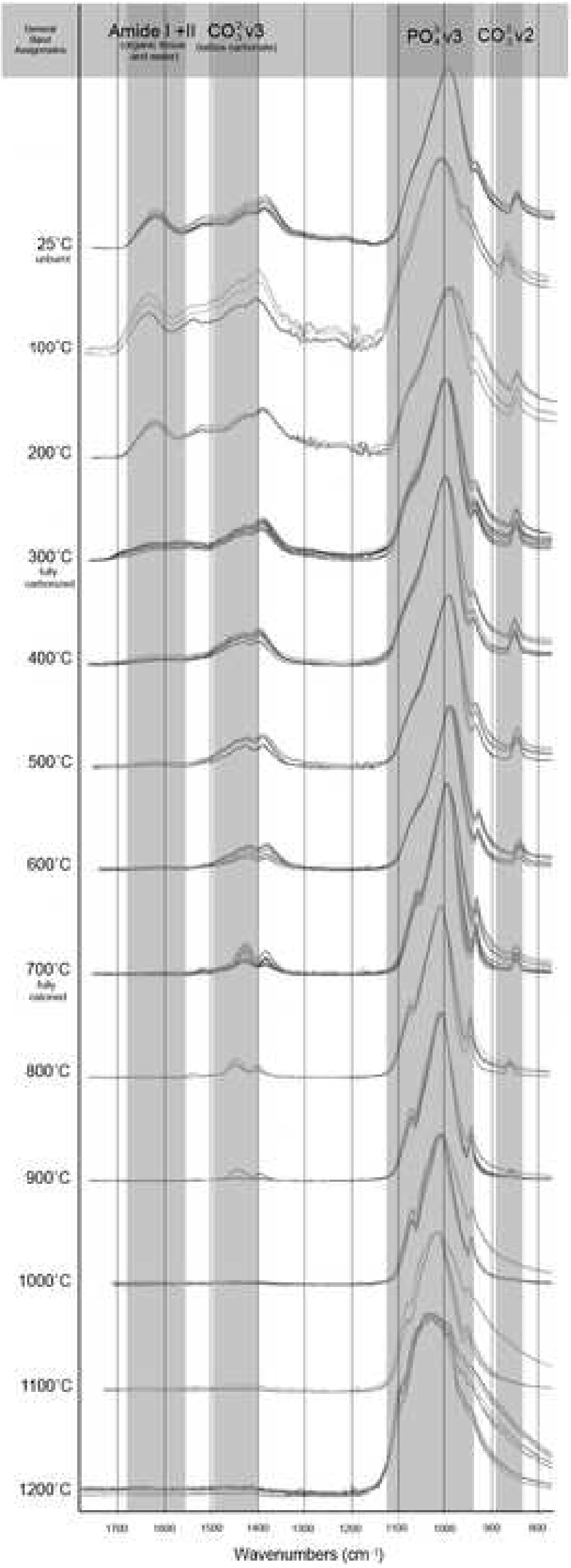

Click here to access/download;Figure;Fig3.tif

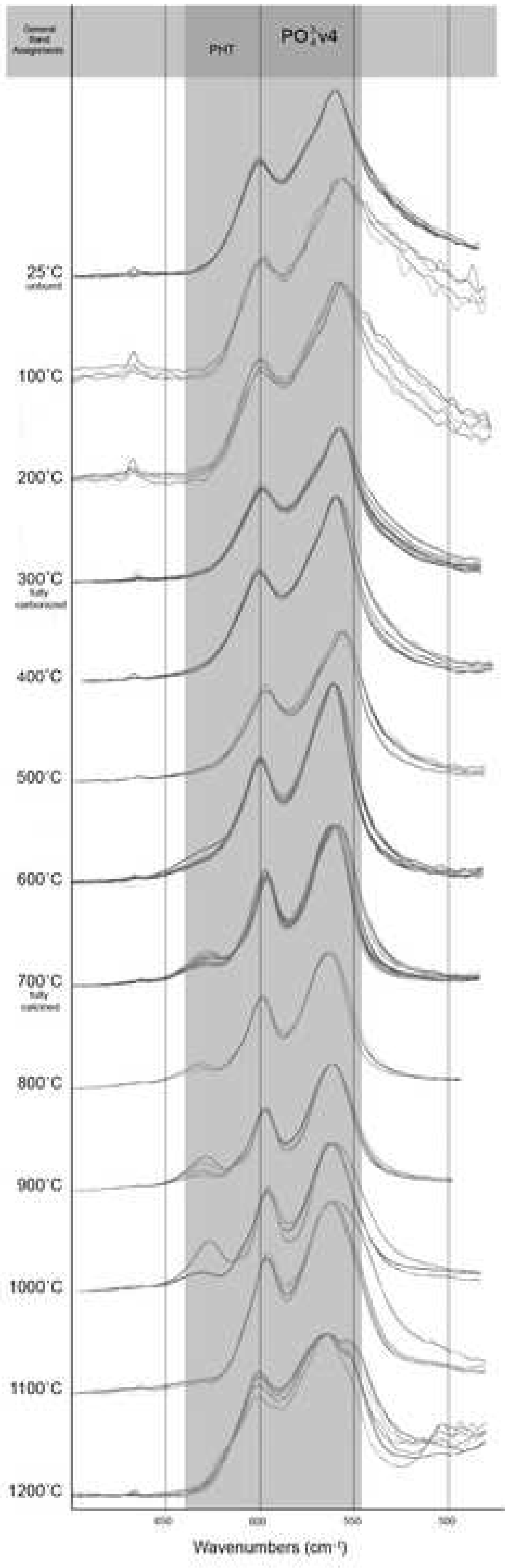

Fig 4

Click here to access/download;Figure;Fig4.tif

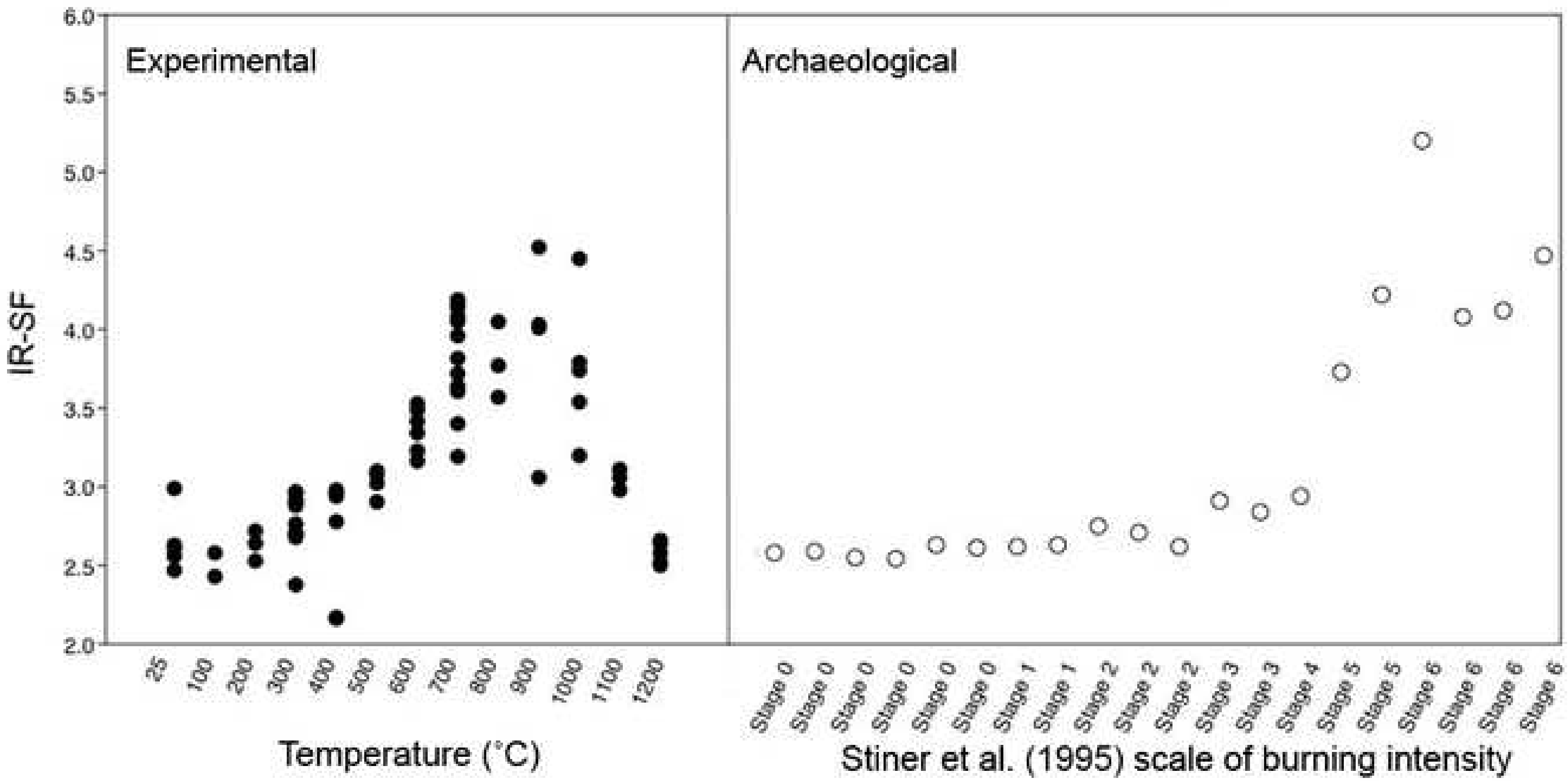

Fig 5

Click here to access/download;Figure;Fig5.tif

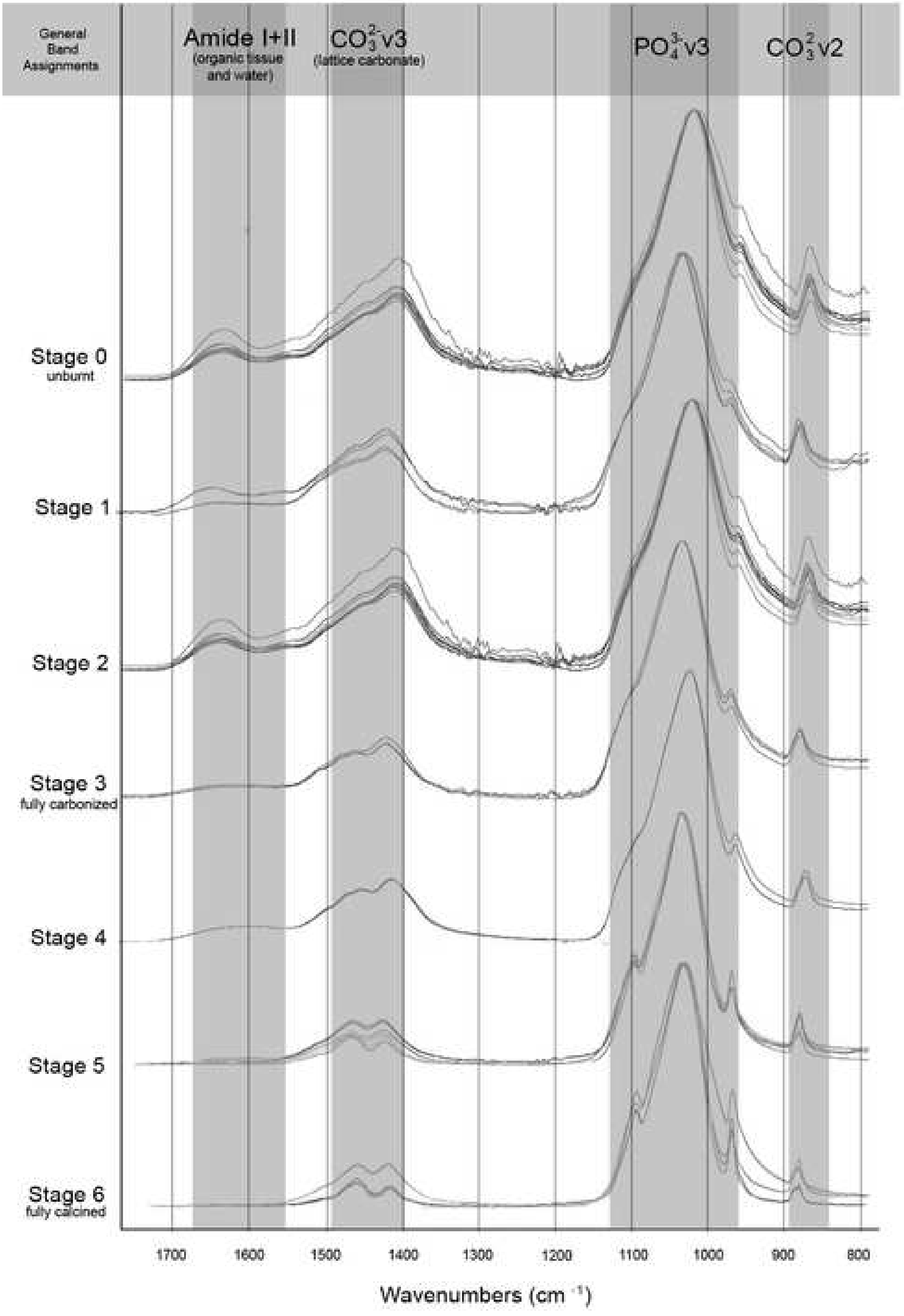

Fig 6

Click here to access/download;Figure;Fig6.tif

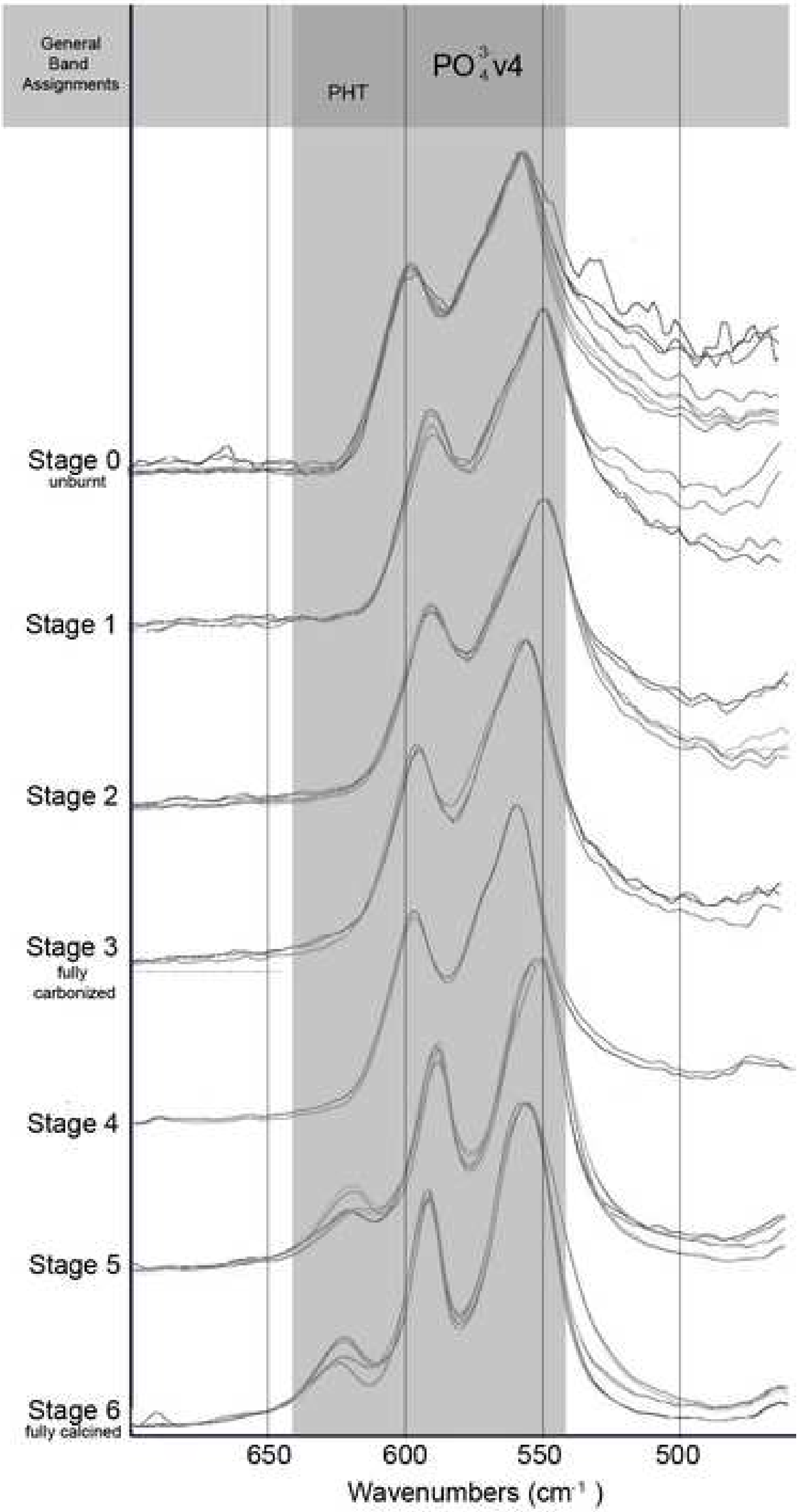

Fig 7

Click here to access/download;Figure;Fig7.tif

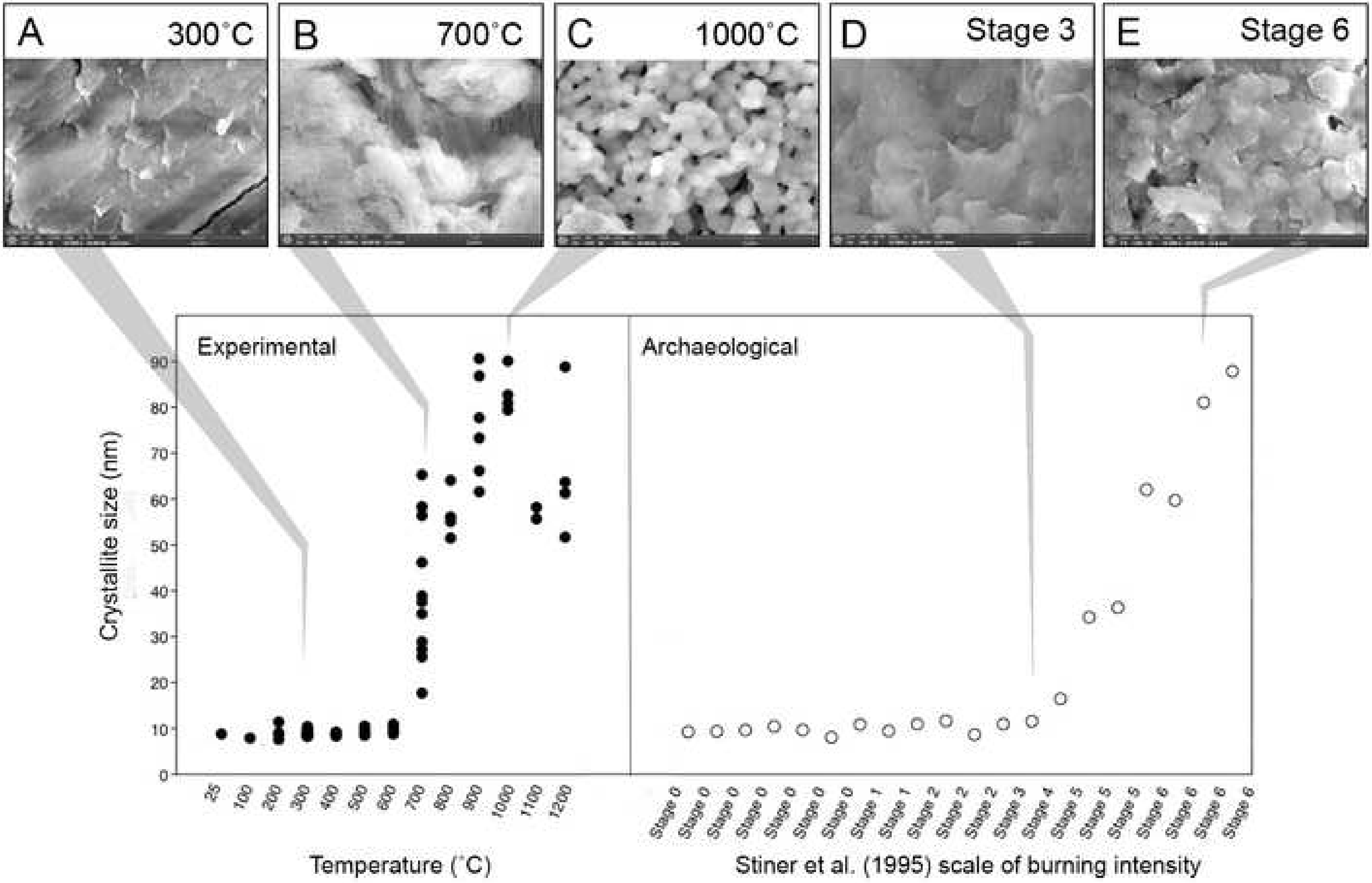

S1 Table

Click here to access/download
Supporting Information
S1 Table.pdf

S2 Table

Click here to access/download
Supporting Information
S2 Table.pdf

S3 Table

Click here to access/download
Supporting Information
S3 Table.pdf

S4 Table

Click here to access/download
Supporting Information
S4 Table.pdf
