# Supplementary material for: Characterization of structural changes in modern and archaeological burnt bone: Implications for differential preservation bias
Source: PLoS One. 2021 Jul 28;16(7):e0254529. doi: 10.1371/journal.pone.0254529 (PMC8318310; doi:10.1371/journal.pone.0254529)
Supplement: Attachment — Submitted filename: PONE-D-20-19845_R1_reviewer1.pdf [file pone.0254529.s008.pdf]

PLOS ONE

Characterization of structural changes in modern and archaeological burnt bone: implications for differential preservation bias

--Manuscript Draft--

**Manuscript Number:**	PONE-D-20-19845R1
**Article Type:**	Research Article
**Full Title:**	Characterization of structural changes in modern and archaeological burnt bone: implications for differential preservation bias
**Short Title:**	Crystallite size growth in modern and archaeological burnt bone
**Corresponding Author:**	Giulia Gallo<br>University of California Davis<br>Davis, CA UNITED STATES
**Keywords:**	archaeology; Zooarchaeology; bone; Burnt Bone; FTIR; XRD
**Abstract:**	Structural and thermodynamic factors which may influence burnt bone survivorship in archaeological contexts have not been fully described. A highly controlled experimental reference collection of fresh, modern bone burned in temperature increments 100-1200˚C is presented here to document the changes to bone tissue relevant to preservation using Fourier transform infrared spectroscopy and X-ray diffraction. Specific parameters investigated here include the rate of organic loss, amount of bone mineral recrystallization, and average growth in bone mineral crystallite size. An archaeological assemblage ca. 30,000 years ago is additionally considered to confirm visibility of changes seen in the modern reference sample and relate changes to commonly used zooarchaeological scales of burning intensity. The timing of our results indicates that the loss of organic components in both modern and archaeological bone burnt to temperatures up to 700˚C are not accompanied by growth changes in the average crystallite size of bone mineral bioapatite, leaving the small and reactive bioapatite crystals of charred and carbonized bone exposed to diagenetic agents in depositional contexts. For bones burnt to temperatures of 700˚C and above, two major increases in average crystallite size are noted which effectively decrease the available surface area of bone mineral crystals, decreasing reactivity and offering greater thermodynamic stability despite the mechanical fragility of calcined bone.
**Order of Authors:**	Giulia Gallo
	Matthew Fyhrie
	Cleantha Paine
	Sergey V. Ushakov
	Masami Izuho
	Byambaa Gunchinsuren
	Nicolas Zwyns
	Alexandra Navrotsky
**Response to Reviewers:**	The following is copied from our Response to Reviewers document uploaded as an attachment.<br><br>Response to Reviewers:<br><br>Reviewer 1<br>…However, the originality of the paper needs to be more explicitly described, crystallinity changes are referred to burning but very little if any is referred to diagenetic influence in burnt archaeological specimens (which is the objective of the paper but finally not clearly discussed). A reorganization of the text and more clear statements and aspects to be discussed to demonstrate the goals of the paper is needed.<br><br>Thank you for your comment and for all your helpful suggestions. The manuscript is

greatly improved with your feedback. To highlight the originality of the paper we have rewritten major portions of the introduction and background sections, attempting to summarize concisely the exact goals of the study and inclusion of the modern and archaeological samples. We believe this will reframe and clarify the role of the Tolbor-17 material for this study, as it is our intention that the Tobor-17 archaeological burnt bone serve as a comparative reference sample of changes in bioapatite crystallinity sizes and organic content for this study. Future zooarchaeological studies planned for Tolbor-17 will consider the hypothesis of differential preservation closely with supporting faunal and geoarchaeological data. We have therefore modified our conclusions to reflect this restructuring, using our results from the study as hypothesis generating observations for further closer study, with the intention to follow up more substantially in the forthcoming full zooarchaeological study of Tolbor-17.

Burning alone does not cause fragmentation, a subsequent movement or effort may produce fragmentation, but indication of which movement or effort acted after burning is not described. Further, fragmentation before and after burning can be caused by a large number of taphonomic agents (e.g. butchery, trampling, weathering, corrosion…).

Thank you for your observation. This perspective and related information will be the major focus of the next study planned for this project: a full zooarchaeological study of the Tolbor-17 faunal material. This planned study will include traditional zooarchaeological analyses including identifiable specimens, fragmentation degree, surface modifications, and preservation. We seek to describe many anthropogenic behaviors present in the Tolbor-17 fauna in the future study, and will pay specific attention to burning to test the hypothesis of differential preservation suggested by our current manuscript. This zooarchaeological investigation has had initial data collection completed in December 2019, with further analyses planned with international travel restrictions are lifted.

Definitively, the paper needs a better and more exhaustive description of the fossil assemblage: number of specimens, size of fossils, surface modifications, how many fossils are in each burning stage or which have also been affected by any other taphonomic agent both before and after burning should be described or included in a table.

We believe that through the reframing and clarification of the purpose of the archaeological material (as a reference sample confirming the visibility in changes of bioapatite crystallinity sizes and organic preservation in an archaeological assemblage and relating described changes to zooarchaeological observational scales of burning intensity) will clarify the intention of the archaeological sample inclusion. A sample of text addressing this specifically is now included in the introduction and is highlighted in the manuscript with tracked changes on page 3. We also have reframed our discussion of the archaeological material in the context of hypothesis generation, and the descriptive fossil assemblage desired here will be the focus of the next paper engaging with the planned zooarchaeological study of the full Tolbor-17 fauna from Unit 3. Language addressing this has been rewritten and is highlighted in page 23 of the manuscript with tracked changes.

Reviewer 2
I like very much the introductory part, it is a very good compilation/resume of the state of the art of bone transformation research. I will suggest to try to resume all the information in a graph where the X axe is the burning temperature/time (qualitative and/or quantitative) and all the mentioned parameter-changes (Y axe) are depicted.

Thank you very much for your time and helpful feedback for our manuscript. It was a concern of ours that the diverse researchers who may be interested in this topic (ranging in disciplines from zooarchaeology to material scientists and thermochemists) may need a full background to provide a comprehensive understanding of the many complex systems being considered, so it was our original intention to have a very extensive background section. From the feedback of our other reviewers, however, it was suggested that this be shortened and made more concise. We believe the revisions have keep the scope and scale of the original intention for the background section while eliminating some more extraneous details and clarifying the language.

Powered by Editorial Manager® and ProduXion Manager® from Aries Systems Corporation

Additionally, we have tried to create several versions of a schematic following your suggestion. All versions were found to be unsatisfactory, both due to the changing scenarios around burning with or without oxygen, and the variance found in reported data regarding porosity which we do not directly investigate in our own study. Our solution was to clarify the language regarding the timing of the described mechanisms, primarily found in our revised discussion section is highlighted on page 22 of the manuscript with tracked changes.

First paragraph of page 13: Describe better the sedimentological/postdepositional features indicating the mentioned features/processes. Any images of the deposits and/or bone remains?

We thank you for your suggestion. We have described the sedimentological context to the extent of our current knowledge, and have revised our text regarding our intention for the archaeological material to be a reference sample into bioapatite crystallinity and organic components of archaeological burned bone. In our forthcoming study on the full zooarchaeological material from Tolbor-17, of which the hypotheses generated by this paper will be tested with further studies, we plan on including a greater amount of detail on the post-depositional taphonomic processes and the greater geological context of Unit 3.

Include/discuss more FTIR-ATR related references for archaeological studies in the discussion, please reivew this recent work and references therein: Iriarte et al., 2020

We are grateful for your encouragement to include the very relevant work of Iriarte et al., 2020. We have now included this work in our discussion of the potential of burnt bone to make advances in our understanding of past human behavior.

Reviewer 3
The abstract is not informative about the results but mostly talk about methods. I suggest to rewrite it.

Thank you for your suggestion, and for your constructive feedback. The abstract has been fully rewritten to address your suggestion.

Introduction: you explain the aims of your study, but I think that a major implication of your work is not considered in this paper. I suggest to discuss in the introduction section (and maybe in the discussion or conclusion) a further implication: are your results relevant in the field of radiocarbon dating on bones or collagen and DNA extraction? I think that you may discuss the importance of changes in crystallinity of bone apatite in the context of radiocarbon dating of archaeological bones.

We thank you for observation, and we have included the discussion of C14 dating of the inorganic component of bone in our revised manuscript. We are grateful for the expansion of the implications and relevance of our study, and this has provided an additional opportunity for us to contextualize and highlight the massive bioapatite mineral structural reorganization of calcined bone. Importantly, it is the same mechanisms which are responsible for the usefulness of calcined bioapatite for C14 dating (thermodynamically stable crystals that can resist contamination) that we describe in this study first at temperatures of calcination (~700˚C), and also a second threshold at higher temperatures (~900˚C). This discussion and many relevant citations is included in our revised section on burnt bone diagenesis which is highlighted on page 10 our manuscript with tracked changes.

We believe our revised text and clarification on the timing of organic loss, and therefore waning usefulness for collagen and DNA extraction, will benefit those audiences as our results provide reference spectroscopic datasets illustrating the presence and quick decay of organic components in burnt bone.

The Background section is really too long. It seems a summary of the background chapters of a PhD dissertation. I suggest to strongly reduce this section and possibly to add some parts to supplementary material or add a new table…

Powered by Editorial Manager® and ProduXion Manager® from Aries Systems Corporation

Thank you. We had major concerns about the background necessary for all varied audiences who may be interested in this paper, and it was our original intention to have a detailed background to meet those needs. Considering your suggestion, we have completely rewritten and restructured our background sections. We believe this revised version still has the breadth to inform readers from different disciplines, but is no longer dense and focused on tangential information.

I noted that you completely missed the many papers published by Gregorio dal Sasso of the University of Padua (Italy). His PhD project was dedicated to the issue of archaeological bones diagenesis after burying and he investigated in details the behaviour of buried bones in terms of changes in crystallinity and interaction with water in the archaeological deposit. I strongly suggest especially to consider the paper elaborating an universal curve for apatite crystallinity (https://www.nature.com/articles/s41598-018-30642-z).

Thank you for your suggestion to reference the research of Gregorio dal Sasso, specifically dal Sasso et al. (2018). We are familiar with the work of dal Sasso and believe the universal curve of apatite crystallinity described in dal Sasso et al. (2018) is a significant recent advancement in the use of spectroscopy to evaluate the preservation of archaeological bone. We plan on using this methodology more extensively in our future work planned on the entire Tolbor-17 faunal assemblage, a comprehensive zooarchaeological study which is forthcoming.

I would also suggest to add (maybe in supplementary material) the section of Tolbon indicating the stratigraphic position of sampled bones. Please condor also that if bones come from different parts of the archaeological despots, they may have suffered different (I mean local) diagenetic process (e.g. related to water percolation or variations of pH values of the host archaeological layer); can this influence your results? the sole map of the study region is completely useless if you don't supply data on the stratigraphy of the site. Finally, it is not clear why you selected this specific archaeological sequence to carry out your experiments.

Thank you for your suggestion. As all burned faunal material sampled comes from a constrained area of one of the Tolbor-17 test pits and was considered to be firmly within the stratigraphic Unit 3, we do not believe this will be a complicating factor for our current study. We do plan on including more detailed sedimentological and geographic information in our more comprehensive zooarchaeological study of the full Tolbor-17 faunal assemblage, however, which will test our hypothesis of preservation bias highlighted by our conclusions further. We have clarified the stratigraphic position of the samples in our text to address this, however, and revised text can be found highlighted on page 13 of our manuscript with tracked changes.

We hope that we have also been able to clarify the inclusion of the Tolbor-17 samples in our study as initial investigations into the novel faunal preservation of Upper Paleolithic burned bone from the Tolbor valley, as well as representative samples of archaeological material sufficient to include as a reference sample to compare to our modern experimental samples. Text to acknowledge this in greater detail can be found highlighted on page 13 in our manuscript with tracked changes.

The description of methods is also very long and many informations are very basic. I suggest to shorten this part of the manuscript or move some parts to supplementary material.

We have taken your comment into serious consideration, and have tried to use more concise language to describe our methodologies. Ultimately much of the basic information have remained due to our interest in providing comprehensive detail to those reading our paper from diverse sub-disciplines who perhaps would like to recreate our study.

Unfortunately, you do not present and discuss results of SEM investigation. Scanning microscope, I guess, may inform about the modification of apatite crystals of your samples; for instance changes in crystal shape or orientation, or can explain the evolution of diagenesis recording the process of recrystallization (evidence of dissolution and recrystallisation are very evident under the SEM). As you cited SEM in

Powered by Editorial Manager® and ProduXion Manager® from Aries Systems Corporation

	the Methods section, I think you should have interesting data to show. The only SEM images are in Fig. 7, but they are not discussed. SEM data would increase also the quality of discussion.<br><br>Thank you. For this study it was our original intention that the SE microscopy images be used for visualization purposes only, and have only selected five bones typical of their burning category for SE microscopy imaging (three from our modern experimental collection, and two from our archaeological assemblage). These bone samples were selected for imaging prior to being powdered for spectroscopic analyses, and it is no longer possible to image a more comprehensive sample of the other included burned modern and archaeological bone due to the powdering which took place. To address your suggestion, we have moved the detailed information regarding the SE microscopy instrument methodology to Supplemental Information and have clarified that the inclusion of SE images in this manuscript are for generalized visualization purposes only. In addition, greater detail of morphological observations are now included in the figure caption to address the processes which are present in our five images. We are excited for the suggestion to include a greater SE microscopy component in our future studies considering the thermal alteration of burnt bone, in which we will image a more thorough and comprehensive sample of material will be considered to conduct analyses and descriptions as you suggested.
**Additional Information:**	
**Question**	**Response**
**Financial Disclosure**<br><br>Enter a financial disclosure statement that describes the sources of funding for the work included in this submission. Review the submission guidelines for detailed requirements. View published research articles from *PLOS ONE* for specific examples.<br><br>This statement is required for submission and **will appear in the published article** if the submission is accepted. Please make sure it is accurate.	NZ, Grant #156074, National Science Foundation (https://www.nsf.gov/awardsearch/showAward?AWD_ID=1560784)<br>MI PaleoAsia Project Grant No. 1802, FY2016–2020) from the Ministry of Education, Culture, Sports, Science and Technology, Japan ("Cultural history of PaleoAsia: Integrative research on the formative processes of modern human cultures in Asia," directed by Yoshihiro Nishiaki) (http://paleoasia.jp/en/)<br>GG UC Davis Cluster Grant "Archaeology and Soil Science Synergy" (https://dhi.ucdavis.edu/events/2019-2020-dhi-transcollege-research-clusters-call-proposals)<br>The funders had no role in study design, data collection and analysis, decision to publish, or preparation of the manuscript.

**Unfunded studies**<br>Enter: *The author(s) received no specific funding for this work.*<br><br>**Funded studies**<br>Enter a statement with the following details:<br>• Initials of the authors who received each award<br>• Grant numbers awarded to each author<br>• The full name of each funder<br>• URL of each funder website<br>• Did the sponsors or funders play any role in the study design, data collection and analysis, decision to publish, or preparation of the manuscript?<br>• **NO** - Include this sentence at the end of your statement: *The funders had no role in study design, data collection and analysis, decision to publish, or preparation of the manuscript.*<br>• **YES** - Specify the role(s) played.<br><br>* typeset	
**Competing Interests**<br><br>Use the instructions below to enter a competing interest statement for this submission. On behalf of all authors, disclose any competing interests that could be perceived to bias this work—acknowledging all financial support and any other relevant financial or non-financial competing interests.<br><br>This statement **will appear in the published article** if the submission is accepted. Please make sure it is accurate. View published research articles from *PLOS ONE* for specific examples.	The authors have declared that no competing interests exist.

Powered by Editorial Manager® and ProduXion Manager® from Aries Systems Corporation

**NO authors have competing interests**<br><br>Enter: *The authors have declared that no competing interests exist.*<br><br>**Authors with competing interests**<br><br>Enter competing interest details beginning with this statement:<br><br>*I have read the journal's policy and the authors of this manuscript have the following competing interests: [insert competing interests here]*<br><br>* typeset	
**Ethics Statement**<br><br>Enter an ethics statement for this submission. This statement is required if the study involved:<br><br>• Human participants<br>• Human specimens or tissue<br>• Vertebrate animals or cephalopods<br>• Vertebrate embryos or tissues<br>• Field research<br><br>Write "N/A" if the submission does not require an ethics statement.<br><br>General guidance is provided below. Consult the submission guidelines for detailed instructions. **Make sure that all information entered here is included in the Methods section of the manuscript.**	This study was carried out with cow bones procured from a local butcher in Sacramento, California, as well as bones removed from horses which were humanely euthanized for purposes other than this study and donated for scientific purposes at the Center for Equine Health at the UC Davis School of Veterinary Medicine in Davis, California.<br><br>The cow bones utilized in this study were from animals slaughtered for meat, and were stunned to render the animal unconscious and unaware of pain, according to the Humane Slaughter Association.<br><br>The humane euthanasia program at the Center for Equine Health at the UC Davis School of Veterinary Medicine is approved by the American Veterinary Medical Association (AVMA) and is done with great care to ameliorate animal suffering. The horses which were humanely euthanized for health purposes were brought to the Center of Equine Health following either a poor prognosis and quality of life following either extreme cervical osteoarthritis or a femur fracture. These horses were euthanized with an intravenous injection of pentobarbital, with prior light sedation and a local anesthetic used to place the intravenous catheter. Specific studies by the comparative neurology group at the UC Davis School of Veterinary Science have determined that the horse rapidly loses all perception of consciousness within ten seconds of administration, and the animals feel no pain or anxiety.<br><br>Subsequent to euthanasia, the Center for Equine Health allowed for tissue collection for necessary scientific purposes. The forelimbs were taken by the JD Wheat Orthopedic Laboratory, where the metacarpals were removed and given to this study presented in this manuscript. Upon inquiry, the JD Wheat Laboratory provided a statement that there was not a committee to approve the use of the legs, as the horses were not euthanized for the purpose of the study.

Powered by Editorial Manager® and ProduXion Manager® from Aries Systems Corporation

**Format for specific study types**<br><br>**Human Subject Research (involving human participants and/or tissue)**<br>• Give the name of the institutional review board or ethics committee that approved the study<br>• Include the approval number and/or a statement indicating approval of this research<br>• Indicate the form of consent obtained (written/oral) or the reason that consent was not obtained (e.g. the data were analyzed anonymously)<br><br>**Animal Research (involving vertebrate animals, embryos or tissues)**<br>• Provide the name of the Institutional Animal Care and Use Committee (IACUC) or other relevant ethics board that reviewed the study protocol, and indicate whether they approved this research or granted a formal waiver of ethical approval<br>• Include an approval number if one was obtained<br>• If the study involved *non-human primates*, add *additional details* about animal welfare and steps taken to ameliorate suffering<br>• If anesthesia, euthanasia, or any kind of animal sacrifice is part of the study, include briefly which substances and/or methods were applied<br><br>**Field Research**<br><br>Include the following details if this study involves the collection of plant, animal, or other materials from a natural setting:<br>• Field permit number<br>• Name of the institution or relevant body that granted permission	
**Data Availability**<br><br>Authors are required to make all data underlying the findings described fully available, without restriction, and from the time of publication. PLOS allows rare exceptions to address legal and ethical concerns. See the PLOS Data Policy and FAQ for detailed information.	Yes - all data are fully available without restriction

Powered by Editorial Manager® and ProduXion Manager® from Aries Systems Corporation

A Data Availability Statement describing where the data can be found is required at submission. Your answers to this question constitute the Data Availability Statement and **will be published in the article**, if accepted.<br><br>**Important:** Stating 'data available on request from the author' is not sufficient. If your data are only available upon request, select 'No' for the first question and explain your exceptional situation in the text box.<br><br>Do the authors confirm that all data underlying the findings described in their manuscript are fully available without restriction?	
**Describe where the data may be found in full sentences. If you are copying our sample text, replace any instances of XXX with the appropriate details.**<br><br>• If the data are **held or will be held in a public repository**, include URLs, accession numbers or DOIs. If this information will only be available after acceptance, indicate this by ticking the box below. For example: *All XXX files are available from the XXX database (accession number(s) XXX, XXX.*).<br>• If the data are all contained **within the manuscript and/or Supporting Information files**, enter the following: *All relevant data are within the manuscript and its Supporting Information files.*<br>• If neither of these applies but you are able to provide **details of access elsewhere**, with or without limitations, please do so. For example:<br><br>*Data cannot be shared publicly because of [XXX]. Data are available from the XXX Institutional Data Access / Ethics Committee (contact via XXX) for researchers who meet the criteria for access to confidential data.*<br><br>*The data underlying the results presented in the study are available from (include the name of the third party*	All relevant data are within the manuscript and its Supporting Information files.

Powered by Editorial Manager® and ProduXion Manager® from Aries Systems Corporation

and contact information or URL).<br>• This text is appropriate if the data are owned by a third party and authors do not have permission to share the data.<br><br>* typeset	
Additional data availability information:	

Powered by Editorial Manager® and ProduXion Manager® from Aries Systems Corporation

Manuscript Click here to access/download;Manuscript;Manuscript.docx

Characterization of structural changes in modern and archaeological burnt bone: implications for differential preservation bias

Authors: Giulia Gallo[1,2*,] Matthew Fyhrie[1,] Cleantha Paine[3], Sergey V. Ushakov[4], Masami Izuho[5], Byambaa Gunchinsuren[6], Nicolas Zwyns[1,2,7], Alexandra Navrotsky[4]

Affiliations:

1- Department of Anthropology, University of California Davis, 95616, Davis CA, USA

2- Center for Experimental Archaeology at Davis, University of California Davis, 95616, Davis CA, USA

3- Archaeology Institute University of the Highlands and Islands, Kirkwall, UK

4- School of Molecular Sciences and Center for Materials of the Universe, Arizona State University, 85287, Tempe AZ, USA

5- Tokyo Metropolitan University, Tokyo, 192-0397, Japan

6- Institute for History and Archaeology, Mongolia Academy of Science, Khukov Street-77, Ulaanbaatar, Mongolia

7- Department of Human Evolution, Max Planck Institute for Evolutionary Anthropology, 04103, Leipzig, Germany.

*Corresponding author

E-mail: gtgallo@ucdavis.edu

Abstract

Structural and thermodynamic factors which may influence burnt bone survivorship in archaeological contexts have not been fully described. A highly controlled experimental reference collection of fresh, modern bone burned in temperature increments 100-1200˚C is presented here to document the changes to bone tissue relevant to preservation using Fourier transform infrared spectroscopy and X-ray diffraction. Specific parameters investigated here include the rate of organic loss, amount of bone mineral recrystallization, and average growth in bone mineral crystallite size. An archaeological assemblage ca. 30,000 years ago is additionally considered to confirm visibility of changes seen in the modern reference sample and relate changes to commonly used zooarchaeological scales of burning intensity. The timing of our results indicates that the loss of organic components in both modern and archaeological bone burnt to temperatures up to 700˚C are not accompanied by growth changes in the average crystallite size of bone mineral bioapatite, leaving the small and reactive bioapatite crystals of charred and carbonized bone exposed to diagenetic agents in depositional contexts. For bones burnt to temperatures of 700˚C and above, two major increases in average crystallite size are noted which effectively decrease the available surface area of bone mineral crystals, decreasing reactivity and offering greater thermodynamic stability despite the mechanical fragility of calcined bone.

Introduction

Anthropogenic burnt bones can be indicative of many social and economic behaviors and can contribute to studies identifying evidence for ritual activity [1], cremations [2-4], bone fuel [5-9], hygienic practices [5,9], cooking and marrow warming [10], and locations of combustion features [11]. Burnt bones undergo substantive structural and compositional changes at different burning intensities, however, and the implications of these changes for differential bone survivorship in an archaeological fauna assemblage is of critical importance for studies utilizing burnt material.

Bone diagenesis can result in bone mineral disintegration or dissolution [12]. The rate, sequence, and extent of diagenetic processes are determined by many factors, including the nature of the depositional environment and the age, element, and species of the bone tissue. Postmortem bone preservation is well described in regards to the differences between compact and cancellous bone [13-16], juvenile and adult bone [17,18], intra- and interspecies variation in bone size and density [19-21], and different environmental conditions [12, 22-26]. However, zooarchaeological evaluations of burnt bone preservation have resulted in varied interpretations and have not addressed the different structural properties of bone burnt to different temperatures [5, 27].

The aim of this study is to describe the range of structural modifications to bone mineral produced by burning at different temperature intensities, and to relate such changes to standardized scales utilized in zooarchaeological methods, specifically here the Stiner et al. [27] scale of burning intensity. This is done with the intention of describing differences between categories of burnt bone, including any vulnerabilities to diagenetic processes that could result in assemblage biases within burnt bone in archaeological contexts. Here we present the results of a controlled experimental reference library of fresh, modern bone burnt in increments of 100-1200˚C and analyzed with Fourier transform infrared spectroscopy (FTIR) with Attenuated Total

Reflectance (ATR) attachment and X-ray diffraction (XRD). We additionally compare spectroscopic measurements to a sample of burnt fauna dated to ca. 30 ka to verify visibility of alterations in an archaeological assemblage.

Bone

Bone is comprised of organic proteins primarily of collagen, inorganic mineral, and water, creating a composite material organized in compact bone in cylindrical structures of concentric lamellae surrounding an interior channel for a central blood canal [28,29]. This hierarchical arrangement provides and maintains the biological roles of skeletal tissue: mechanical strength to transmit force and protect organs, and the regulation of homeostasis through ionic regulation [28-31].

Living bone is very porous, with around 12% of bone volume comprised of open spaces [2]. The concentric systems, known as Haversian systems, constitute a large percentage of the bone matrix porosity, with the remainder composed of resorption bays and voids created between the organic and inorganic components [29-32]. The amount, size, and density of pores in bone is variable across elements, species, and ages, although trabecular bone does exhibit a higher porosity than compact bone due to its more open structure [21, 29, 32].

Inorganic bone constituent, bioapatite, is isostructural to mineral hydroxyapatite $Ca_5(PO_4)_3OH$. The specific chemical compositions of bioapatite reflect diet, biological age through history of bone remodeling, and variation can exist both within species and within the skeletal elements themselves [29]. Bioapatite contains 5-8 wt% carbonate which can substitute either phosphate or hydroxyl group in hydroxyapatite structure [33]. Bioapatite has a high degree of nonstoichiometry, and its composition can be described as $Ca_{10-x}(PO_4)_{6-x}(HPO_4, CO_3)x(OH,1/2CO_3)_{2-x}$ with $0<x<2$ [31, 34, 35].

In vivo bioapatite has extremely small, thin, plate-like morphologies (1-7 nm thick, 15-200 nm in length, and 10-80 nm in width) which are cross-linked to organic collagen fibrils [34-36]. Water is found in bone as loose mobile water in the extracellular matrix, in void spaces to facilitate movement, and integrated within and around the organic and mineral components [31, 37]. Bioapatite crystallites have typical surface areas above 200 m^2g and are heavily hydrated [30, 31, 35-38]. These surface layers of ions play a key role in the regulation of homeostasis, as they can be easily exchanged and provide a necessary capacity to regulate ionic concentrations in living tissue [3, 28, 38-41].

Bone diagenesis

Diagenesis, the postmortem changes to bone tissue in burial environments, includes the integrated processes of microbial attack, water activity, and mineral recrystallization and can result in the complete disintegration of bone material [12]. The arrangement and size distribution of pores at the time of burial are large predictors of bone decay or bone survival, as pores mediate the access and extent of destructive agents such as bacteria and water [18, 22-24, 42-44]. Microbial attack itself is an active and immediate process accounting for a large amount of initial organic destruction, especially in warmer environments [42]. Microbial access to collagen degrades the protein chains, effectively removing the organic component of bone [19, 24, 32, 42, 44, 45]. The removal of the collagen component results in a more brittle biomaterial on the macroscale, and leaves bioapatite crystals unprotected on the micro- and nanoscale [46].

Exposed bioapatite is vulnerable to the incorporation of impurities and to disintegration, as postmortem crystals initially retain the specific morphology, reactivity, and thermodynamic instability of living bioapatite [47, 48]. The reaction between bone mineral and water is the most significant predicate of bioapatite disintegration at this stage of diagenesis, and bones buried in environments with active water movement are highly vulnerable to leeching and dissolution, noted to be heightened when bioapatite is exposed and easily accessible after organic removal [24, 48]. There is no universal thermodynamic model of bioapatite solubility due to the complexity of bone as a biomaterial, and rather each crystal domain is assigned its own Metastable Equilibrium Solubility (MES): a distribution phenomenon dependent on aspects of bone quality such as carbonate substitutions, ion vacancies, low crystallinity, and small crystal sizes [48, 49]. Uptake of contamination from the burial environment, such as rare earth elements and secondary calcite, has been noted to reduce as bone mineral spontaneously recrystallizes without in-vivo regulation and larger crystals grow at the expense of smaller crystals [18, 50, 51]. This process results in bone mineral with a slightly higher crystallinity, effectively decreasing the available reactive surface area of bioapatite and therefore the overall solubility compared to fresh bone [18, 36, 50, 51].

Burnt bone

Burning bone results in the decomposition of the organics and loss of water, as well as in massive changes to bioapatite crystal dimensions and structure. The extent and degree of these alterations are correlated to temperature and burning atmosphere, producing bones with different mechanical and thermodynamic properties dependent on the extent of burning. These micro- and nano- scale transformations have a notable impact on visible macroscopic changes to heat altered bone, including color changes, cracking, shrinkage, weight loss, and fragmentation [27, 41, 52-55]. Burnt bone coloration is generally correlated to burning intensity, and the ease of color identification has assisted in the proliferation and use of zooarchaeological scales of coding heat alteration, such as the Stiner et al. [27] classification of burning intensity (Table 1).

Table 1: Burning intensity scale based on macroscopic visual qualities following Stiner et al. [27]

Burning Scale	Description
0	Not burnt
1	Slightly burnt, < 50% carbonized
2	Majority burnt, > 50% carbonized
3	Fully carbonized
4	Slightly highly burnt, < 50% calcined
5	Majority highly burnt, > 50% calcined
6	Fully calcined

Scales of burning based on macroscopic visual cues are tremendously beneficial for processing archaeological assemblages of burnt fauna, but do not reflect the sequence of changes in the composition and structural properties of burnt bone. Observations on the nano- and micro-scale have therefore led to the definition of four stages of burning which are correlated to the transformation of bone mineral and removal of organics on the nano- and microscale: dehydration, decomposition, inversion, and fusion [2, 54-57]. These stages are accomplished at different temperature thresholds and were defined in oxidizing burning conditions [2, 54-57]. The rate and degree of temperature induced changes depend on variables such as flesh coverage, heating and cooling rates and oxygen availability [54, 55]. Dehydration, or, the loss of water, occurs between 100 and 600˚C [2, 54, 56-58]. This wide temperature range likely accounts for the quicker loss of the loosely bound water between 25 and 250˚C and the eventual loss of the additional water more structurally bound to the mineral in temperatures above 100˚C [2, 31, 56, 57].

After initial dehydration, the second stage of bone combustion is organic decomposition, from 300 to 800˚C [2, 56, 57]. With collagen degradation starting at 112 - 260˚C, above 300˚C a large proportion of the organics is reduced to a char [58]. Between 300 and 500˚C most mass, 50 – 55 %, is lost, and above 500˚C any remaining char is removed by 700˚C [58]. The macroscopic transformation most noticeable with the decomposition stage is the striking changes in coloring, with bone becoming visibly blackened with the charring of organics (300˚C), corresponding to Stages 1-3 of the Stiner et al. [27] scale, and after the complete removal of organics (700˚C) transitioning to a grey and chalky white hue for Stiner et al. [27] Stages 4-6 [2, 27, 54, 56, 57]. Bone that is blackened is referred to as combusted or carbonized dependent on burning atmosphere, while grey and white bone with all organics removed can be referenced as calcined [27, 54, 55].

Simultaneous to the loss of organics is the alteration of the bioapatite mineral, or the inversion stage, between 500 and 1100˚C [2, 56, 57]. With the removal of the organic component at 300˚C, the larger, plate-like crystals can spontaneously grow at the expense of smaller crystals [2, 56-58]. Experiments with bone burnt while powdered and subsequently cleaned with acetone report mean crystallite size increasing to 10 - 30 nm, and crystallite thickness moving from 2 to 9 nm [58, 59]. Above 500˚C, additional growth has been observed, with reported crystallite sizes plateauing at 110 nm and with crystal thickness reaching 10 nm [58]. The crystals, transforming from platelet like to hexagonal, later become equiaxed at 900˚C, growing more spheroidal with overall dimensions reaching 300 - 550 nm [59].

The last stage of heat alteration to bioapatite, fusion, accounts for the microstructural changes noted with the inversion phase above 700˚C [2, 56, 57]. Bone porosity initially increases from the originally porous *in vivo* status with the loss and charring of organics (~300˚C), which also corresponds to a loss in bone density [32, 59]. Carbonized and charred bone is reported to be most porous right before temperatures of calcination (600˚C) [32]. Beginning at 700˚C there is a densification as the bioapatite crystal grains grow, and by 900˚C there is a total structural coalescence from the additional crystal growth, resulting in an interlocking structure and a marked decrease in porosity [2, 58, 59].

These changes are all products of burning in oxidizing conditions [55]. If a bone is brought to temperatures greater than 300˚C without access to oxygen, a different pattern of thermal alteration has been demonstrated in controlled experiments [55]. When heating occurs in reducing atmospheres, the organic char is not removed and instead becomes more ordered [55]. The crystallinity of the bioapatite does increase, however, although at a slower rate than indicated in oxidizing conditions [55]. New compounds, such as cyanamide, are also likely formed around 600˚-700˚C [55]. Bones burnt in reducing atmospheres above 600˚C do not lose the organic char component, and therefore remain black in coloring [55].

Burnt bone diagenesis

The rapid morphological and compositional changes to burnt bone tissue are similar to changes seen over prolonged periods of time in the diagenesis of unburnt bone. This includes the removal of organic components and incorporated water, as well as the recrystallization of the bioapatite crystals. The immediate and greater extent of these changes in bone burnt to both low and high temperatures, however, results in a markedly different biomaterial at time of burial than unburnt bone.

Burnt bone is more fragile than unburnt bone, with fragmentation a function of burning intensity [27]. The dehydration and eventual complete removal of collagen from bone tissue significantly changes the toughness and strength properties of bone, altering the density, the structural integrity, and the stress and strain relationship [27, 60-62]. This ultimately results in a greater likelihood of mechanical fracture correlated to the amount of collagen lost, leaving calcined bone the most mechanically vulnerable [27, 60]. Due to this extreme friability, recovered burnt bone fragments do not reflect initial size at deposition and processes such as burial and trampling can severely and easily fragment burnt bone [27, 63].

The fragility, likely presence of small fragment sizes, and elimination of organic components of bone burnt to lower temperatures provides greater surface area and easy access for diagenetic agents in the context of burial environments. Bone mineral does, however, undergo tremendous crystallite growth and reorganization with burning at higher temperatures, enabling calcined bone to be protected from contamination [54, 58, 64, 65]. Because of this, calcined bone is recognized to be the most reliable source of inorganic C14 for radiocarbon dating, as the elevated crystallinity that accompanies heat alteration at high temperatures protects the Type A and B carbonate substitutions and secondary carbonate incorporated from the burning atmosphere from further alteration, which subsequently can be used to date the burning event [64, 66].

Questions about the changing vulnerabilities of differentially burnt bone prompted our investigations into the characterization of structural changes of modern and archaeological bone burnt at different temperatures. Of specific interest to this study is the timing of the organic loss, and therefore loss of bioapatite protection, in reference to the increases in crystallinity and crystal sizes of bone mineral. Archaeological bone, both unburnt and burnt, is considered in this study as an actualistic reference to relate implications to commonly used zooarchaeological scales of burning intensity, and to monitor the extent of alterations related to the spontaneous postmortem recrystallization of bone mineral which occurs over time in burial environments.

Materials and methods

Modern bone sample collection and preparation

A controlled experimental reference collection was created with modern bone to investigate the timing and impact of thermal alteration on organic loss, recrystallization indices, and crystallite size growth. Cortical bone from three cow femurs and two horse metacarpals from five different individuals were selected for this study. Cow femurs were procured the day after butchery from a local butcher and were never frozen. Flesh was scraped manually to prepare for drilling. Horse metacarpals were obtained postmortem from completed forelimb tissue collections of horses humanely euthanized for purposes other than this study at the UC Davis School of Veterinary Medicine. Metacarpals were simmered in water with the addition of borax to assist with defleshing, although bones remained greasy.

A diamond drill coring bit was used to produce solid plugs of cortical bone 3mm x 3mm, with weights ranging from 53.2 to 58.1 mg. Coring was constrained to the cortical bone tissue from the mid-diaphysis of both cow and horse bones. Solid bone plugs were specifically utilized in lieu of bone powder to avoid the effects of powder heating, as powder has an increased surface area and would be more reactive to thermal alteration. To fit the dimensions of crucibles used for thermal analyses, plugs were filed with diamond files. Post-experimental heating, three samples were selected for imaging with Scanning Electron (SE) microscopy for visualization purposes. All bone samples were then powdered with an agate mortar and pestle and sieved with 234 μm mesh.

Modern bone thermal analysis

The controlled annealing of modern bone samples was performed with Setaram Labsys Evo thermal analyzer. Bone core samples were placed in a 100 μl Al_2O_3 crucible and air flow 40 ml/min was established. The samples were brought to desired temperatures from 100 to 1200°C in 100˚C increments, with heating rate 20˚C /min and held isothermally for 30 minutes. The weight change and heat flow traces were recorded continuously and corrected for the baseline. Additional samples were produced at 300 and 700˚C with ramp 50°C /min and one hour dwell time for comparison.

Archaeological case study sample collection and preparation

Archaeological unburnt and burnt bone samples were collected from the site of Tolbor-17, an open-air locale on a low altitude pass on the western flank of the Khangai Mountains of Northern Mongolia. The Ikh-Tolborin-Gol is part of the Selenga drainage system, the main river feeding Lake Baikal (Fig 1) [67]. This river valley preserves a wealth of Upper Paleolithic (UP) locales including Tolbor-4, Tolbor-15, Tolbor-16, and Tolbor-17 (T-17) [67-70]. Most of the sites document periodic human occupations starting with the Initial Upper Paleolithic, ca. 45 ka, until the Holocene. The latter has recently been dated with polymineral post-IR IRSL, Quartz OSL, and radiocarbon to 42.5-45.6 ka, establishing the timing for a movement of population between the Siberian Altai and Northwestern China, contemporaneous with the earliest *Homo sapiens* fossils in the region [67]. The following occupations in the valley are most likely associated with *Homo sapiens*, and are characterized as Upper Paleolithic (UP) in the broad sense. Although it is often assumed that fire is part of the modern human behavioral repertoire allowing expansion into cold climates, evidence of the use of fire in the UP Tolbor locales is rare and has been only briefly reported [71, 72].

Fig 1. Map of Mongolia with geographic position of Tolbor-17. Map modified after Geo-atlas.

Tolbor-17 provides a rare opportunity to investigate faunal remains, as organic material is usually poorly preserved in the region and burnt fauna has not yet been described in detail. Like most of the other locales, T-17 is an open-air environment with a fluctuating low energy run-off, constituting a fluctuating recharge water regime [24, 44]. Initially excavated as a series of two test pits with dimensions 2 m x 1 m, the excavators at T-17 piece plotted all finds > 2 cm, and the remaining sediment from each bucket volume of excavated material was dry sieved with 4 mm and 2 mm mesh screens, with all material subsequently sorted. The T-17 lithological Unit 3 is characterized by the presence of UP lithic artifacts and organic faunal preservation, despite sedimentary evidence for episodic sheet erosion, prolonged groundwater interaction, chemical weathering, and long surface exposure. Based on its geological setting, the material studied here belongs to the second half of the Marine Isotope Stage (MIS) 3, ca. 40 -30 ka cal. BP and is described as UP. Unburnt and burnt fauna have been successfully recovered from Unit 3; however, this assemblage is extremely fragmentary and traditional zooarchaeological analyses based on taxonomic identification and prey selection and processing are still in the preliminary stages.

Mapped (> 2 cm) and screened (< 2 cm-2 mm) faunal remains from the T-17 UP assemblage were cleaned, sorted following the Stiner et al. [27] seven stage visual scale of burning intensity, and weighed (Table 2). No burning was noted in bone > 2 cm except for a single fragment, but fauna < 2 cm- 2mm recovered in the screened material is found to span all stages of burning intensity within Unit 3 of the exposed excavation surface (Table 2). Of the burnt fauna, a large percentage is nearly or fully calcined, a notable observation due to the recognized mechanical fragility of calcined bone and the unprotected open-air environment of T-17. All excavated fauna was assigned burning stages following Stiner et al. [27] and 20 bones from the same test pit of the Unit 3 assemblage were sub-sampled. A minimum of one category representing bones from this sample were selected for subsequent spectroscopic analyses to confirm heat alteration and investigate organic composition, crystallinity, and crystallite size. Bones were cleaned with ionic water sonication, and two samples representing before and after calcination were selected for imaging with SE microscopy prior to all samples being powdered with a diamond file and an agate mortar and pestle. All archaeological bone powder samples were then sieved with 234 μm mesh. No permits were required for the described study, which complied with all relevant regulations.

Table 2: T17 Unit 3 fauna burning summary.

	Screened (< 2 cm)	Piece plotted (>2 cm)	Total
Burning Stage	Weight (g)	Weight (g)	Weight (g)
0	141.36	213.31	354.47
1	1.04	0	1.04
2	5.17	1.01	6.18
3	0.69	0	0.69
4	1.95	0	1.95

5	1.37	0	1.37
6	4.5	0	4.5

Burning stages following Stiner et al.[27].

Infrared spectroscopy data collection and analysis

FTIR spectroscopy is a semi-quantitative method which characterizes bond vibrations, absorbed at specific wavelengths of transmitted incident light from infrared radiation, to identify compositional and structural properties of materials [54, 73]. When applied to bone, FTIR spectroscopy can yield valuable information regarding the presence and quality of preserved organic components, as well as the relative degree of structural order, size, and strain of bioapatite crystals [74-78]. This is particularly useful for the detection of organic preservation in samples screened prior to radiocarbon and stable isotope studies, and for diagenetic studies evaluating the integrity of bone mineral [77-78].

FTIR spectroscopy has additionally had success identifying thermally altered bone, as changes to bone composition and bioapatite crystallinity can be monitored though several heat induced peak transformations which cannot be mistaken for macroscopic staining or bleaching [4, 33, 53-55, 79, 80]. The identification of FTIR spectral peaks associated with the thermal alteration of bone has been extensively documented, with major alterations monitored through: (1) the ratio of carbonate to phosphate present in the sample, the C/P ratio, (2) the depletion of the presence of amide I and II functional groups, representing the organic components of bone, and (3) the presence of heat specific peak splitting, such as the loss of the peak at 874 cm^{-1} correlated to CO_3^{2-} v2 at temperatures over 1000˚C, and the PHT shoulder peak at temperatures over 700˚C [54, 55, 57, 81-83]. Measures of the crystallinity of a sample can be inferred from the infrared splitting factor (IRSF), which extrapolates the changing size and order of bioapatite crystals through increase of splitting seen in the PO_4^{3-} v4 peaks [54, 57, 81].

Specific peaks relevant to this study and their inferred functional groups include the 1650 cm^{-1} and 1550 cm^{-1} peaks for the measurement of amide I and II, the 874 cm^{-1} and 1415 cm^{-1} peaks indicating presence of the v2 and v3 of carbonate, and the 900-1200 cm^{-1} and 50-600 cm^{-1} spectral regions related to the v3 and v4 phosphate components (Table 3). Additionally, the appearance of a 625cm^{-1} shoulder peak is attributed here to PO_4^{3-} v4 bending, known as the phosphate high temperature (PHT) [57].

Table 3: FTIR-ATR wavenumbers associated with likely functional groups relevant to this study and the thermal alteration of bone.

Wavenumber	Inferred peak assignment	Peak transformation relevant to this study
1630-1660 cm^{-1}	organic tissue and water, amide I + II	decrease and absence
1400-1550 cm^{-1}	CO_3^{2-} v3	1415 cm^{-1} peak a component of C/P ratio
1028-1100 cm^{-1}	PO_4^{3-} v3	1035 cm^{-1} peak a component of the C/P ratio
874 cm^{-1}	CO_3^{2-} v2	peak loss

565 cm^{-1}, 605 cm^{-1}	PO_4^{3-} v4	growth of 565 cm^{-1} and 605 cm^{-1} and decrease of the 595 cm^{-1} trough utilized for the infrared splitting factor (IRSF); phosphate high temperature (PHT) shoulder growth at 625 cm^{-1}

A Nicolet 6700 Fourier transform infrared spectrometer with an ATR attachment and a deuterated triglycine sulfate (DTGS) detector and single bounce diamond crystal was used. The ATR method uses an attachment with a diamond or zinc crystal to produce spectra through the phenomenon of internal reflectance [84-86]. The application of ATR minimizes sample preparation, which in turn minimizes contamination [73, 82, 84]. Spectra were collected with 256 scans in the 4000 - 400 cm $^{-1}$ frequency region and with an 8 mm spectral range. Each archaeological and modern bone powder sample was retested for quality control.

Eight peak measurements were monitored for 168 scans representing 84 individual samples for this study, 62 modern and 22 archaeological. Each sample was tested twice, and measurements presented here represent the average values of both scans. FTIR-ATR spectra were processed with OMNIC software.

The IRSF measurements were procured for all samples following Weiner and Bar-Yosef [81].

$$\text{Infrared Splittng Factor:} \frac{(565\ \text{cm}^{-1}\ peak\ ht + 605\ \text{cm}^{-1}\ peak\ ht)}{595\ \text{cm}^{-1}\ \ peak\ ht}$$

An additional measure of the carbonate to phosphate content, the C/P ratio, was also determined for all samples. The C/P ratio decreases with burning and utilizes the 1035 cm^{-1} phosphate peak unaffected by IRSF changes [73, 82].

$$\frac{\text{C}}{\text{P}}\ \text{ratio:} \frac{1415\ \text{cm}^{-1}\ peak\ ht}{1035\ \text{cm}^{-1}\ peak\ ht}$$

Other peaks observed for this analysis were noted as they are related to the loss of organics and specific heat-induced changes [54, 55, 57] (Table 3).

X-ray Diffraction

X-ray Diffraction (XRD) can be used to measure the relative sizes of bioapatite crystals [32, 54, 58, 59]. Powder XRD patterns here were obtained using Bruker D2 Phaser and Bruker D8 advance diffractometers using CuKα radiation. Bone powder samples taken from the solid bone plugs and archaeological fauna were spread with ethanol on a zero background silicon sample holder, and run from 10 to 90 °2θ with 0.02° step. Dwell time was chosen to obtain at least thousand counts on the most intense peaks. The average crystallite size of analyzed samples was obtained from diffraction peaks broadening using whole pattern fitting (Rietveld refinement) procedure as implemented in Jade MDI software [87]. Diffraction profile was modeled using hydroxyapatite $Ca_5(PO_4)_3OH$ structure (space group P63/m) and pseudo-Voigt profile shape function. The instrumental broadening was accounted for by calibration with NIST LaB_6 profile

shape standard. The uncertainties in crystallite sizes are reported as obtained from least squares refinement.

Results

FTIR modern samples

The FTIR-ATR spectra were obtained for each sample, 25-1200˚C. All modern samples above 200˚C were found to exhibit spectra indicative of the thermal alteration of bone in oxygen atmospheres supported by previous research (Figs 2 and 3), including the decrease of C/P ratio, decrease of organic components by 300˚C with complete absence seen by 400˚C, the absence the 874 cm^{-1} peak above 1000˚C, and the presence of the PHT peak splitting above 700˚C (S1 Table, S2 Table). No differences were indicated in the reheated or increased rate samples taken to 300 and 700˚C from the single-heated or controlled rate counterparts.

Fig 2. FTIR-ATR spectra of experimentally modern burnt samples grouped by with functional groups highlighted in the range of 1700-800 cm^{-1}.

Fig 3. FTIR-ATR spectra of experimentally modern burnt samples with functional groups highlighted in the range of 700-500 cm^{-1}.

The IRSF of all modern samples also followed reported trends in bioapatite crystallinity, with order, size and strain increasing alongside intensifying temperatures and clearly demonstrated with the presence of calcination (Figs 2, 3, 4; S1 and S2 Tables) [54, 56]. This increase in crystallinity is seen until 1000˚C, after which there is a marked decrease in IRSF coinciding with the equiaxing of bioapatite crystals (Fig 4). Despite the acceptance of the IRSF metric and general consensus with previously described trends, the values of modern IRSF here do exhibit large variations [54, 56]. This is seen most dramatically in the range of IRSF values reported for all samples at 900˚C in this study (Fig 4; S2 Table). No changes in crystallinity were detected in samples which were reheated or heated with increased rates.

Fig 4. Infrared Splitting Factor (IRSF) of the experimental modern and archaeological collection measured from FTIR-ATR spectra following Weiner and Bar Yosef [81].

FTIR archaeological samples

The FTIR-ATR spectra produced from the T-17 archaeological collection is consistent with the Stiner et al. [27] stages of temperature intensity assignments based on color alteration. Unburnt bone is supported as non-heat altered and burnt bone does not indicate signs of intrusive staining or bleaching (Figs 5 and 6). Good agreement is seen with the relative decreases of C/P ratio and the loss of organic components by Stage 3 (fully carbonized) between the archaeological and modern samples (Figs 5 and 6; S3 and S4 Tables). The appearance of the PHT with bones identified as Stage 5 supports the presence of temperatures above 700˚C at T-17, although the continued presence of CO_3^{2-} v2 inferred by the 874 cm^{-1} peak indicates temperatures likely did not reach above 1000˚C (Figs 5 and 6; S3 Table).

Fig 5. FTIR-ATR spectra of archaeological fauna from T-17 grouped by stage of burning intensity following Stiner et al. [27] with functional groups highlighted in the range of 1700-800 cm^{-1}.

Fig 6. FTIR-ATR spectra of archaeological fauna from T-17 grouped by stage of burning intensity following Stiner et al. [27] with functional groups highlighted in the range of 700-500 cm^{-1}.

The IRSF of the T-17 samples also follows the trends of the experimental modern collection, with gradual increases seen through Stage 3 (fully carbonized) and demonstrably higher values reported with the presence of calcination at Stages 4, 5, and 6 (Fig 4; S3 and S4 Tables). Elevated values are not seen within the Stage 0 unburnt samples of T-17 bone, demonstrating that the spontaneous recrystallization of bone mineral that accompanies diagenesis does not exceed here values of modern or archaeological samples which have been burnt at low or high temperatures. As expected, the IRSF values can distinguish between calcined and non-calcined samples but cannot distinguish between low temperature burning samples.

XRD modern samples

The results of the XRD analyses on the modern samples demonstrate the increasing crystallite size correlated to temperature, specifically above temperatures of calcination (700˚C) (Fig 7; S2 Table). An average size threshold is clearly noted, with all samples unburnt through 600˚C averaging 9 nm, while all samples burnt at 700˚C jump to an average of 41 nm (Fig 7). An additional increase in crystallite size by approximately 30 nm is noted at 900˚C, coinciding with the fusion stage of thermal alteration of bone, with samples reaching an average of 72 nm (Fig 7). The XRD results also demonstrate that within fully calcined samples, corresponding to Stiner et al. [27] burning scale Stage 6, two temperature thresholds can be distinguished: the maximum crystal size of calcined bone >700˚C not exceeding 70 nm, and the maximum crystal size of calcined bone >900-1200˚ C exceeding 90 nm (Fig 7). This study therefore suggests average crystallite size as a metric to distinguish temperatures obtained within calcined bone classified as Stiner et al. Stage 6, > 700˚C and > 900˚C.

Fig 7. XRD results of averaged crystallite size (nm) alongside selected SE images of experimental modern (A-C) and archaeological (D-E) bone to visualize the changes to crystal shape with heat alteration. Images A and D highlight the small, platelike shape of bioapatite burned to at least 300˚C. Images B and C, both modern, demonstrate morphological range of bones burnt above 700˚C, both of which are fully calcined and are considered Stage 6 on the Stiner et al. [27] scale of burning intensity. The initial growth of hexagonal crystals ~700˚C (B) and eventual equaixing and further growth of crystals above 900˚C (C) are clearly seen in images B and C, and when compared to the archaeological sample of a fully calcined Stage 6 bone (E), the bioapatite crystal morphology corresponds to the size and shape of experimental bones burned above 900˚C. Magnification and instrument details described in S1 Appendix.

XRD archaeological samples

The average crystallite size measurements for the archaeological T-17 bioapatite samples confirm that the natural postmortem recrystallization of unburnt archaeological bone does not exceed crystal sizes of modern or archaeological bones burnt to low temperatures. The crystallite sizes do increase considerably with the presence of calcination at Stiner et al. [27] Stages 4, 5, and 6 and can be distinguished from lower temperature burning and unburnt bone (Fig 7; S4 Table). Utilizing the metric of crystal size, we also suggest that for two of the archaeological samples of Stage 6 fully calcined bone there is evidence of burning > 900˚C - <1100˚C.

Discussion

The measurements presented here regarding the loss of organics, the degree of crystallinity, and the crystallite sizes are in consensus with trends reported by previous studies, with small variance likely introduced by the burning of solid bone versus powdered samples and different experimental sample preparation and burning regimes. Our results demonstrate that for bone burnt to lower temperatures before calcination, Stiner et al. [27] Stages 1-3, bioapatite retains the small reactive crystal sizes of unburnt bone but faces rapid organic loss. The depletion of the organic component is most complete just prior to temperatures of calcination (~700˚C). This disintegration of the collagen in charred or carbonized bone is recognized to produce a very open porosity, leaving bone mineral burnt highly exposed [58].

The increase in crystallinity and crystal size growth seen by 700˚C corresponding to calcination and Stiner et al., [27] Stages 4-6 immediately reduces the surface to mass ratio and active surface area of bone mineral. This lower surface area results in a product with a lower solubility potential than unburnt and charred or carbonized bone. For fully calcined Stage 6 bones which have also been exposed to temperatures above 900˚C in oxygen atmospheres, there is the additional benefit of reported compaction and closing of the porosity, further limiting the access of any destructive agents to the exposed crystallites [58]. The heat induced dimensional changes are also considerably larger than the small amounts of diagenetic recrystallization which assists with the prevention of contamination in fresh bone, as evidenced by the inclusion of unburnt archaeological samples

Diagenetic agents such as water dissolution are therefore inferred here to be a serious threat to bone burnt to temperatures under 700˚C, because partially and wholly carbonized and charred burnt bone has low levels of crystallinity and small crystals which preserve a reactive surface area similar to unburnt fresh bone at the time of deposition. The additional lack of organic protection, open porosity, and likely small fragment sizes due to the recognized friability of burnt bones calls attention to the further vulnerability of charred and carbonized fauna in Stiner et al. [27] Stages 1-3 due to bioapatite dissolution and disintegration. Correspondingly, the lack of organic presence in burnt bones, especially bones corresponding to Stiner et al. [27] Stages 3-6 is interpreted here to be a less attractive target for the diagenetic agent of microbial action than fresh and fleshed unburnt bone.

The results of this study propose a hypothesis of differential survivorship in bones burnt to different temperature thresholds in assemblages of archaeological fauna with fluctuating water movement. This study would further suggest that bones with low density and high porosity, such as trabecular and juvenile bone, are further susceptible to dissolution if charred or carbonized. This is due to the assumption that open porosities, likely small fragment sizes, and no organic protection exacerbates the access of diagenetic agents such as fluctuating water to their small and reactive crystals.

Bone which has been calcined, including trabecular and juvenile bone, potentially has a greater likelihood to resist dissolution due to the large crystal sizes and greater thermodynamic stability. These properties have been similarly recognized to contribute to the suitability of calcined bone for inorganic C14 dating analyses [64]. Calcined bone has been shown to be more mechanically fragile than both unburnt and charred or carbonized bone, however, and traumatic action including post-depositional movement and trampling can be highly destructive. Calcined

bone therefore may be expected to preserve well in a variety of depositional environments, albeit in small fragment sizes recoverable only through screening.

Further work on the nature of burning and a full zooarchaeological profile of T-17 Unit 3 can test the hypothesis of differential survivorship, as the open-air site does have sedimentary evidence of active and fluctuating water movement. Preliminary faunal analyses indicate that there is a very low presence of bone in the Stiner et al. [27] 1-3 stages, but calcined bone identified as Stage 6 is recovered in comparatively considerable amounts (Table 2). Future work considering archaeological assemblages of burnt bone as proxies for natural and anthropogenic fire therefore should consider biases that may influence the differential survivorship of burnt bone, as properties of burnt bone vary widely between burning thresholds.

Conclusion

The spectroscopic measurements reported here encompass a large reference collection of burning in highly controlled conditions 100-1200˚C in fresh solid bone cores, extending previous modern and archaeological datasets that consider the structural and compositional changes to burnt bone. Especially of note is the average crystallite size difference between bone burnt above 700˚C, a metric which may be used to distinguish different burning temperatures within fully calcined archaeological bone, which are all considered as Stiner et al. [27] Stage 6.

Our results highlight the vulnerability of charred and carbonized bone to diagenetic agents and generate hypotheses regarding the differential survivorship of bone burnt to different temperatures. Here the nature of the hydrological environment is proposed to be a significant threat to bone burnt to low temperatures, as water fluctuation is a large factor in fresh bone bioapatite dissolution and charred and carbonized bone has similar bone mineral properties but has had organic protection already eliminated prior to burial.

This study reiterates the importance of small bone fragments in the study of ancient fire, observations also noted in previous studies [27,88]. Excluding the screened faunal material from T-17 would have obscured the presence of fire almost entirely, as all burnt bone except for a single fragment was recovered in the screened fraction likely due to the friability of burnt bone. The identification and recognition of further biases in addition to the mechanical fragility of burnt bone, and the variation within bones burnt to different temperatures, is emphasized here as burnt bone exhibits a range of structural and thermodynamic properties across zooarchaeological scales of visible burning intensity.

While burnt bone can provide valuable and detailed information on the properties of ancient fire relevant to behaviors of interest, the presence of fire which used fuel with low burning temperatures, such as grass, and from environments with fluctuating water interaction may not have surviving faunal evidence. Therefore, future studies should consider potential differential survivorship in archaeological assemblages of burnt bone, the impact of biased survivorship, and therefore the visibility of burnt fauna.

Acknowledgements:

The authors would like to thank Dr. Teresa Steele for her continued feedback and illuminating discussions, and Cody Prang and Chris Gallo for their many helpful explanations. We additionally thank Drs. Eerkens, Darwent, and Parikh for their generosity and lab resources, as well as the patience, assistance, and expertise of the Peter A. Rock Thermochemistry Laboratory, NEAT research group, and the Parikh Environmental Soil Chemistry Group at the University California Davis. The authors also recognize the support given from the UC Davis Veterinary Medical Teaching Hospital, the UC Davis JD Wheat Veterinary Orthopedic Laboratory, the UC Davis AMCaT Laboratory, the UC Davis DHI Transdisciplinary Research Cluster "The Cluster for Archaeology and Soil Synergy", the Prehistory department of Université Liège, and the Center for Experimental Archaeology at UC Davis (CEAD).

References

1. Mentzer, S. M., Romano, D. G., & Voyatzis, M. E. (2017). Micromorphological contributions to the study of ritual behavior at the ash altar to Zeus on Mt. Lykaion, Greece. *Archaeological and Anthropological Sciences*, *9*(6), 1017-1043.
2. Thompson T. Recent advances in the study of burned bone and their implications for forensic anthropology. Forensic Science International. 2004;146:S203–5.
3. Ubelaker DH. The forensic evaluation of burned skeletal remains: A synthesis. Forensic Science International. 2009 Jan;183(1–3):1–5.
4. Iriarte, E., García-Tojal, J., Santana, J., Jorge-Villar, S. E., Teira, L., Muñiz, J., & Ibañez, J. J. (2020). Geochemical and spectroscopic approach to the characterization of earliest cremated human bones from the Levant (PPNB of Kharaysin, Jordan). *Journal of Archaeological Science: Reports*, *30*, 102211.
5. Costamagno S, Théry-Parisot I, Brugal J-P, Guibert R. Taphonomic consequences of the use of bones as fuel. Experimental data and archaeological applications. In: Biosphere to lithosphere: new studies in vertebrate taphonomy. Oxbow Books Oxford; 2005. p. 51–62.
6. Morin E. Taphonomic implications of the use of bone as fuel. Palethnologie. 2010;2:209-17.
7. Théry-Parisot I. Fuel management (bone and wood) during the Lower Aurignacian in the Pataud rock shelter (Lower Palaeolithic, Les Eyzies de Tayac, Dordogne, France). Contribution of experimentation. Journal of Archaeological Science. 2002;29(12):1415–21.
8. Schiegl S, Goldberg P, Pfretzschner H-U, Conard NJ. Paleolithic burnt bone horizons from the Swabian Jura: Distinguishing between in situ fireplaces and dumping areas. Geoarchaeology. 2003;18(5):541–65.
9. Costamagno S, Théry-Parisot I, Castel JC, Brugal JP. Combustible ou non? Analyse multifactorielle et modèles explicatifs sur des ossements brûlés paléolithiques. *Geston des combustibles au Paléolithique et au Mésolithique: nouveaux outils, nouvelles interpretations*. 2009:61

10. Speth J, Clark J. Hunting and overhunting in the Levantine Late Middle Palaeolithic. Before Farming. 2006 Jan;2006(3):1–42.
11. Barkai R, Rosell J, Blasco R, Gopher A. Fire for a reason: Barbecue at middle Pleistocene Qesem cave, Israel. Current Anthropology. 2017 Aug 1;58(S16):S314-28.
12. Turner-Walker G. The Chemical and Microbial Degradation of Bones and Teeth. In: Pinhasi R, Mays S, editors. Advances in Human Palaeopathology [Internet]. Chichester, UK: John Wiley & Sons, Ltd; 2007:3–29.
13. Binford LR, Bertram JB. Bone frequencies and attritional processes. In (LR Binford, Ed.) For Theory Building in Archaeology. 1977.
14. Lyman RL. Bone density and differential survivorship of fossil classes. Journal of Anthropological archaeology. 1984;3(4):259–99.
15. Grayson DK. Bone transport, bone destruction, and reverse utility curves. Journal of Archaeological Science. 1989 Nov 1;16(6):643–52.
16. Outram AK. A new approach to identifying bone marrow and grease exploitation: why the "indeterminate" fragments should not be ignored. Journal of archaeological science. 2001;28(4):401–10.
17. Munson PJ, Garniewicz RC. Age-mediated survivorship of ungulate mandibles and teeth in canid-ravaged faunal assemblages. Journal of Archaeological Science. 2003;30(4):405–16.
18. Robinson S, Nicholson RA, Pollard AM, O'Connor TP. An Evaluation of Nitrogen Porosimetry as a Technique for Predicting Taphonomic Durability in Animal Bone. Journal of Archaeological Science. 2003 Apr;30(4):391–403.
19. Von Endt DW, Ortner DJ. Experimental effects of bone size and temperature on bone diagenesis. Journal of Archaeological Science. 1984 May;11(3):247–53.
20. Lam YM, Chen X, Pearson OM. Intertaxonomic variability in patterns of bone density and the differential representation of bovid, cervid, and equid elements in the archaeological record. American Antiquity. 1999;64(2):343–62.
21. Lyman RL. Vertebrate taphonomy. Cambridge University Press; 1994.
22. Nielsen-Marsh CM, Hedges REM. Patterns of Diagenesis in Bone I: The Effects of Site Environments. Journal of Archaeological Science. 2000 Dec;27(12):1139–50.
23. Nielsen-Marsh CM, Smith CI, Jans MME, Nord A, Kars H, Collins MJ. Bone diagenesis in the European Holocene II: taphonomic and environmental considerations. Journal of Archaeological Science. 2007 Sep;34(9):1523–31.
24. Nielsen-Marsh C, Gernaey A, Turner-Walker G, Hedges R, Pike A, Collins M. The chemical degradation of bone. Human Osteology in archaeology and forensic science. 2000: 439-454.
25. Denys C. Taphonomy and experimentation. Archaeometry. 2002 Aug;44(3):469–84.
26. Maurer A-F, Person A, Tütken T, Amblard-Pison S, Ségalen L. Bone diagenesis in arid environments: An intra-skeletal approach. Palaeogeography, Palaeoclimatology, Palaeoecology. 2014 Dec;416:17–29.
27. Stiner MC, Kuhn SL, Weiner S, Bar-Yosef O. Differential Burning, Recrystallization, and Fragmentation of Archaeological Bone. Journal of Archaeological Science. 1995 Mar;22(2):223–37.
28. Martin RB, Burr DB, Sharkey NA, Fyhrie DP. Skeletal tissue mechanics. Second edition. New York: Springer; 2015. 762 p.

29. Stout SD, Cole ME, Agnew AM. Histomorphology. In: Ortner’s Identification of Pathological Conditions in Human Skeletal Remains [Internet]. Elsevier; 2019 [cited 2019 Oct 20]. p. 91–167.
30. Rollin-Martinet S, Navrotsky A, Champion E, Grossin D, Drouet C. Thermodynamic basis for evolution of apatite in calcified tissues. American Mineralogist. 2013 Nov 1;98(11–12):2037–45.
31. Drouet C, Aufray M, Rollin-Martinet S, Vandecandelaère N, Grossin D, Rossignol F, et al. Nanocrystalline apatites: The fundamental role of water. American Mineralogist. 2018 Apr 1;103(4):550–64.
32. Figueiredo M, Fernando A, Martins G, Freitas J, Judas F, Figueiredo H. Effect of the calcination temperature on the composition and microstructure of hydroxyapatite derived from human and animal bone. Ceramics International. 2010 Dec;36(8):2383–93
33. Greiner M, Rodríguez-Navarro A, Heinig MF, Mayer K, Kocsis B, Göhring A, et al. Bone incineration: An experimental study on mineral structure, colour and crystalline state. Journal of Archaeological Science: Reports. 2019;25:507–18.
34. Rey C, Combes C, Drouet C, Glimcher MJ. Bone mineral: update on chemical composition and structure. Osteoporos Int. 2009 Jun;20(6):1013–21.
35. Bala Y, Farlay D, Boivin G. Bone mineralization: from tissue to crystal in normal and pathological contexts. Osteoporos Int. 2013 Aug;24(8):2153–66.
36. Berna F, Matthews A, Weiner S. Solubilities of bone mineral from archaeological sites: the recrystallization window. Journal of archaeological Science. 2004 Jul 1;31(7):867-82.
37. Nyman JS, Ni Q, Nicolella DP, Wang X. Measurements of mobile and bound water by nuclear magnetic resonance correlate with mechanical properties of bone. Bone. 2008;42(1):193–9.
38. Trueman CN, Privat K, Field J. Why do crystallinity values fail to predict the extent of diagenetic alteration of bone mineral? Palaeogeography, Palaeoclimatology, Palaeoecology. 2008 Sep;266(3–4):160–7.
39. Koeppenkastrop D, Eric H. Sorption of rare-earth elements from seawater onto synthetic mineral particles: An experimental approach. Chemical geology. 1992;95(3–4):251–63.
40. Reynard B, Lécuyer C, Grandjean P. Crystal-chemical controls on rare-earth element concentrations in fossil biogenic apatites and implications for paleoenvironmental reconstructions. Chemical Geology. 1999;155(3–4):233–41.
41. Buikstra JE, Swegle M. Bone modification due to burning: experimental evidence. Bone modification. 1989;247–58.
42. Tripp JA, Squire ME, Hedges REM, Stevens RE. Use of micro-computed tomography imaging and porosity measurements as indicators of collagen preservation in archaeological bone. Palaeogeography, Palaeoclimatology, Palaeoecology. 2018 Dec;511:462–71.
43. Smith CI, Nielsen-Marsh CM, Jans MME, Collins MJ. Bone diagenesis in the European Holocene I: patterns and mechanisms. Journal of Archaeological Science. 2007;34(9):1485–93.
44. Hedges REM, Millard AR. Bones and Groundwater: Towards the Modelling of Diagenetic Processes. Journal of Archaeological Science. 1995 Mar;22(2):155–64.
45. Hedges REM. Bone diagenesis: an overview of processes. Archaeometry. 2002 Aug;44(3):319–28.

46. Wescott, D. J. (2019). Postmortem change in bone biomechanical properties: Loss of plasticity. *Forensic science international*, *300*, 164-169.
47. Trueman CN, Behrensmeyer AK, Potts R, Tuross N. High-resolution records of location and stratigraphic provenance from the rare earth element composition of fossil bones. Geochimica et Cosmochimica Acta. 2006 Sep;70(17):4343–55.
48. Baig AA. Influences of carbonate content and crystallinity on the solubility behavior of synthetic and biological apatites. Department of Pharmaceutics and Pharmaceutical Chemistry, University of Utah; 1997.
49. Baig AA, Fox JL, Wang Z, Higuchi WI, Miller SC, Barry AM, et al. Metastable Equilibrium Solubility Behavior of Bone Mineral. Calcified Tissue International. 1999 Apr 1;64(4):329–39.
50. Trueman, C. N., & Tuross, N. (2002). Trace elements in recent and fossil bone apatite. *Reviews in mineralogy and geochemistry*, *48*(1), 489-521.
51. Pfretzschner H-U. Fossilization of Haversian bone in aquatic environments. Comptes Rendus Palevol. 2004 Oct;3(6–7):605–16.
52. Shipman P, Foster G, Schoeninger M. Burnt bones and teeth: an experimental study of color, morphology, crystal structure and shrinkage. Journal of Archaeological Science. 1984 Jul;11(4):307–25.
53. Thompson T, Ulgium, P. Burned Human Remains. In Handbook of Forensic Anthropology and Archaeology. Routledge. 2016: 295-303.
54. Ellingham STD, Thompson TJU, Islam M, Taylor G. Estimating temperature exposure of burnt bone — A methodological review. Science & Justice. 2015 May;55(3):181–8.
55. Reidsma FH, van Hoesel A, van Os BJH, Megens L, Braadbaart F. Charred bone: Physical and chemical changes during laboratory simulated heating under reducing conditions and its relevance for the study of fire use in archaeology. Journal of Archaeological Science: Reports. 2016 Dec;10:282–92.
56. Thompson TJU. The Analysis of Heat-Induced Crystallinity Change in Bone. In: The Analysis of Burned Human Remains. Elsevier; 2015. p. 323–37.
57. Thompson TJU, Islam M, Bonniere M. A new statistical approach for determining the crystallinity of heat-altered bone mineral from FTIR spectra. Journal of Archaeological Science. 2013 Jan;40(1):416–22.
58. Etok SE, Valsami-Jones E, Wess TJ, Hiller JC, Maxwell CA, Rogers KD, et al. Structural and chemical changes of thermally treated bone apatite. J Mater Sci. 2007 Sep 21;42(23):9807–16.
59. Pramanik S, Hanif A, Pingguan-Murphy B, Abu Osman N. Morphological Change of Heat Treated Bovine Bone: A Comparative Study. Materials. 2013. Dec 21;6(1):65–75.
60. Waterhouse, K. (2013). The effect of weather conditions on burnt bone fragmentation. *Journal of forensic and legal medicine*, *20*(5), 489-495.
61. Herrmann, N. P., & Bennett, J. L. (1999). The differentiation of traumatic and heat-related fractures in burned bone. *Journal of Forensic Science*, *44*(3), 461-469.
62. Wang, X., Bank, R. A., TeKoppele, J. M., Hubbard, G. B., Althanasiou, K. A., & Agrawal, C. M. (2000). Effect of collagen denaturation on the toughness of bone. *Clinical Orthopaedics and Related Research®*, *371*, 228-239.
63. McKinley, J. I. (1994). Bone fragment size in British cremation burials and its implications for pyre technology and ritual. *Journal of Archaeological Science*, *21*(3), 339-342.

64. Lanting, J. N., Aerts-Bijma, A. T., & van der Plicht, J. (2001). Dating of cremated bones. *Radiocarbon*, *43*(2A), 249-254.
65. Major, I., Dani, J., Kiss, V., Melis, E., Patay, R., Szabó, G., ... & Jull, T. A. (2018). Adoption and evaluation of a sample pretreatment protocol for radiocarbon dating of cremated bones at HEKAL. *Radiocarbon*.
66. Zazzo, A., & Saliège, J. F. (2011). Radiocarbon dating of biological apatites: a review. *Palaeogeography, Palaeoclimatology, Palaeoecology*, *310*(1-2), 52-61.
67. Zwyns N, Paine CH, Tsedendorj B, Talamo S, Fitzsimmons KE, Gantumur A, et al. The Northern Route for Human dispersal in Central and Northeast Asia: New evidence from the site of Tolbor-16, Mongolia. Sci Rep. 2019 Dec;9(1):11759.
68. Zwyns N, Gladyshev SA, Gunchinsuren B, Bolorbat T, Flas D, Dogandžić T, et al. The open-air site of Tolbor 16 (Northern Mongolia): Preliminary results and perspectives. Quaternary International. 2014 Oct;347:53–65.
69. Gladyshev SA, Olsen JW, Tabarev AV, Jull AJT. The Upper Paleolithic of Mongolia: Recent finds and new perspectives. Quaternary International. 2012 Dec;281:36–46.
70. Tabarev A., Gunchinsuren .B, Gillam J., Gladyshev S., Dogandžić T., Zwyns N. Kompleks Pamiatnikov Kamennogo Veka v Doline r.Ikh Tulberiin-Gol, Severnaya Mongolia (razvedochnye raboty s ispolzovaniem GIS-Technnologii v. 2011 g) [The Stone Age site complex in the Ikh-Tulberiin-Gol River Valley, North Mongolia (survey and research using GIS-technology in 2011)]. Studia Archaeologica Instituti Archaeologici Academiae Scientiarum Mongolicae. 2012;32:26–43
71. Gowlett JAJ. The early settlement of northern Europe: Fire history in the context of climate change and the social brain. Comptes Rendus Palevol. 2006 Jan;5(1–2):299–310.
72. Rybin EP, Khatsenovich AM, Gunchinsuren B, Olsen JW, Zwyns N. The impact of the LGM on the development of the Upper Paleolithic in Mongolia. Quaternary International. 2016 Dec;425:69–87.
73. Beasley MM, Bartelink EJ, Taylor L, Miller RM. Comparison of transmission FTIR, ATR, and DRIFT spectra: implications for assessment of bone bioapatite diagenesis. Journal of Archaeological Science. 2014 Jun;46:16–22.
74. Lebon, M., Zazzo, A., & Reiche, I. (2014). Screening in situ bone and teeth preservation by ATR-FTIR mapping. *Palaeogeography, Palaeoclimatology, Palaeoecology*, *416*, 110-119.
75. Lebon, M., Reiche, I., Gallet, X., Bellot-Gurlet, L., & Zazzo, A. (2016). Rapid quantification of bone collagen content by ATR-FTIR spectroscopy. *Radiocarbon*, *58*(1), 131.
76. Trueman, C. N., Behrensmeyer, A. K., Tuross, N., & Weiner, S. (2004). Mineralogical and compositional changes in bones exposed on soil surfaces in Amboseli National Park, Kenya: diagenetic mechanisms and the role of sediment pore fluids. *Journal of Archaeological Science*, *31*(6), 721-739.
77. Dal Sasso, G., Asscher, Y., Angelini, I., Nodari, L., & Artioli, G. (2018). A universal curve of apatite crystallinity for the assessment of bone integrity and preservation. *Scientific reports*, *8*(1), 1-13.
78. Gianfrate, G., D'Elia, M., Quarta, G., Giotta, L., Valli, L., & Calcagnile, L. (2007). Qualitative application based on IR spectroscopy for bone sample quality control in radiocarbon dating. *Nuclear Instruments and Methods in Physics Research Section B: Beam Interactions with Materials and Atoms*, *259*(1), 316-319.

79. Lebon, M., Reiche, I., Fröhlich, F., Bahain, J. J., & Falguères, C. (2008). Characterization of archaeological burnt bones: contribution of a new analytical protocol based on derivative FTIR spectroscopy and curve fitting of the ν 1 ν 3 PO 4 domain. *Analytical and Bioanalytical Chemistry*, *392*(7-8), 1479-1488.
80. Snoeck, C., Lee-Thorp, J. A., & Schulting, R. J. (2014). From bone to ash: Compositional and structural changes in burned modern and archaeological bone. *Palaeogeography, palaeoclimatology, palaeoecology*, *416*, 55-68.
81. Weiner S, Bar-Yosef O. States of preservation of bones from prehistoric sites in the Near East: A survey. Journal of Archaeological Science. 1990 Mar;17(2):187–96.
82. Thompson TJU, Gauthier M, Islam M. The application of a new method of Fourier Transform Infrared Spectroscopy to the analysis of burned bone. Journal of Archaeological Science. 2009 Mar;36(3):910–4.
83. Paschalis EP, DiCarlo E, Betts F, Sherman P, Mendelsohn R, Boskey AL. FTIR microspectroscopic analysis of human osteonal bone. Calcified tissue international. 1996 Dec 1;59(6):480-7.
84. Hollund HI, Ariese F, Fernandes R, Jans MME, Kars H. Testing an alternative high-throughput tool for investigating bone diagenesis: FTIR in attenuated total reflection (ATR) mode: An alternative tool for investigating bone diagenesis. Archaeometry. 2013 Jun;55(3):507–32.
85. Nakamoto K. Infrared and Raman Spectra of Inorganic and Coordination Compounds (Part A: Theory and Applications in Inorganic Chemistry)(Volume 1A)(Part B: Applications in Coordination, Organometallic, and Bioinorganic Chemistry)(Volume 1B). NY, John Wiley & Sons, Incorporated; 1997
86. Bruno TJ. Sampling Accessories for Infrared Spectrometry. Applied Spectroscopy Reviews. 1999 Jul 20;34(1–2):91–120.
87. Rietveld H. A profile refinement method for nuclear and magnetic structures. Journal of applied Crystallography. 1969 Jun 2;2(2):65-71.
88. Villa P, Castel JC, Beauval C, Bourdillat V, Goldberg P. Human and carnivore sites in the European Middle and Upper Paleolithic: similarities and differences in bone modification and fragmentation. Revue de paléobiologie. 2004;23(2):705-30.

Supporting information

S1 Table: Experimental modern bone FTIR-ATR relevant peak height values.

S2 Table: Calculated experimental modern sample values of FTIR C/P, IRSF, and bioapatite crystal size average as measured from XRD.

S3 Table: FTIR-ATR Unit 3 archaeological sample relevant peak height values.

S4 Table: Calculated archaeological T-17 Unit 3 C/P and IRSF values, and bioapatite crystal size average as measured from XRD.

S1 Appendix: SE microscopy imaging instrument and magnification details.

Figure

Click here to access/download;Figure;Fig 1.tif

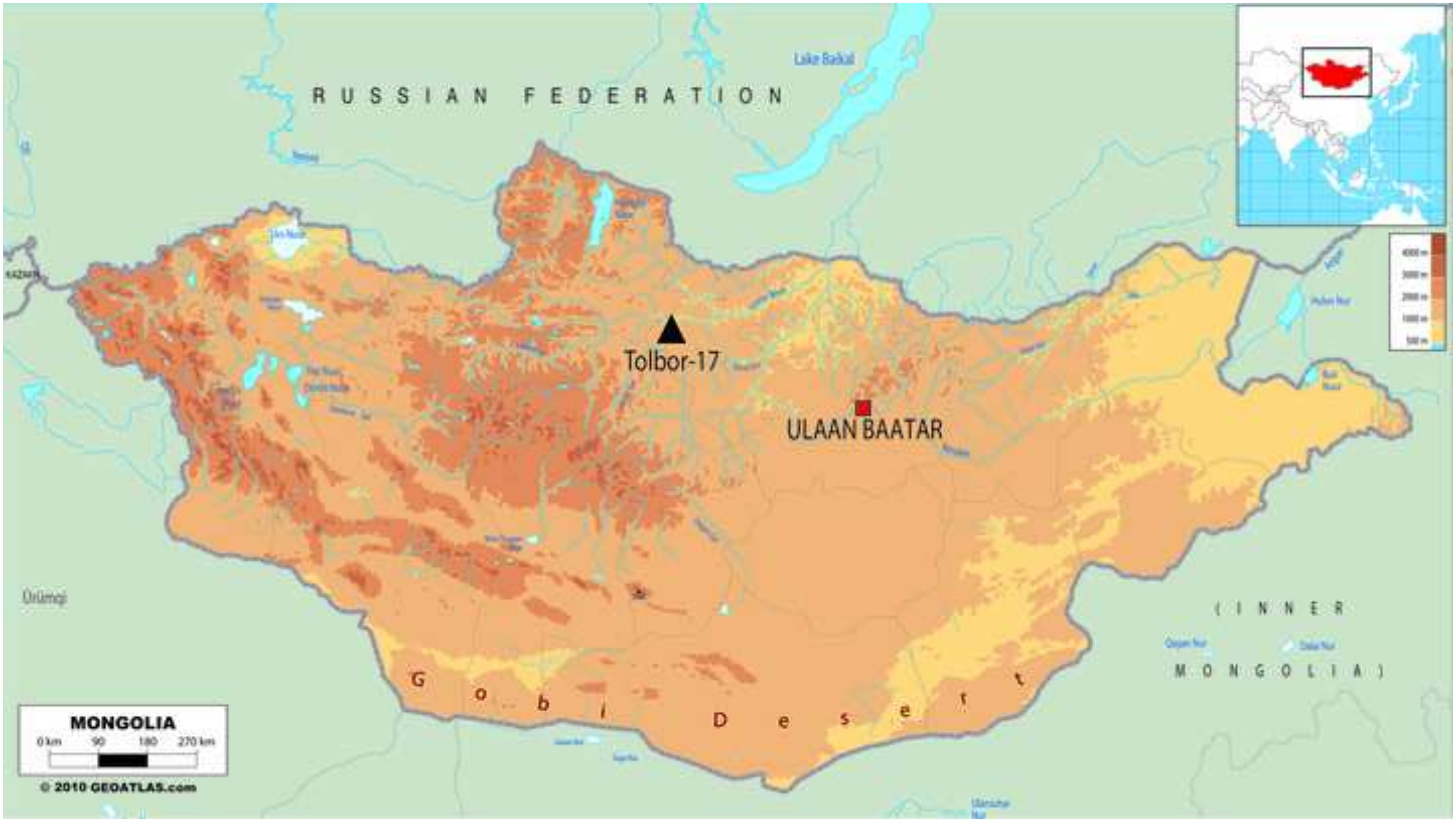

Figure Click here to access/download;Figure;Fig 2.tif

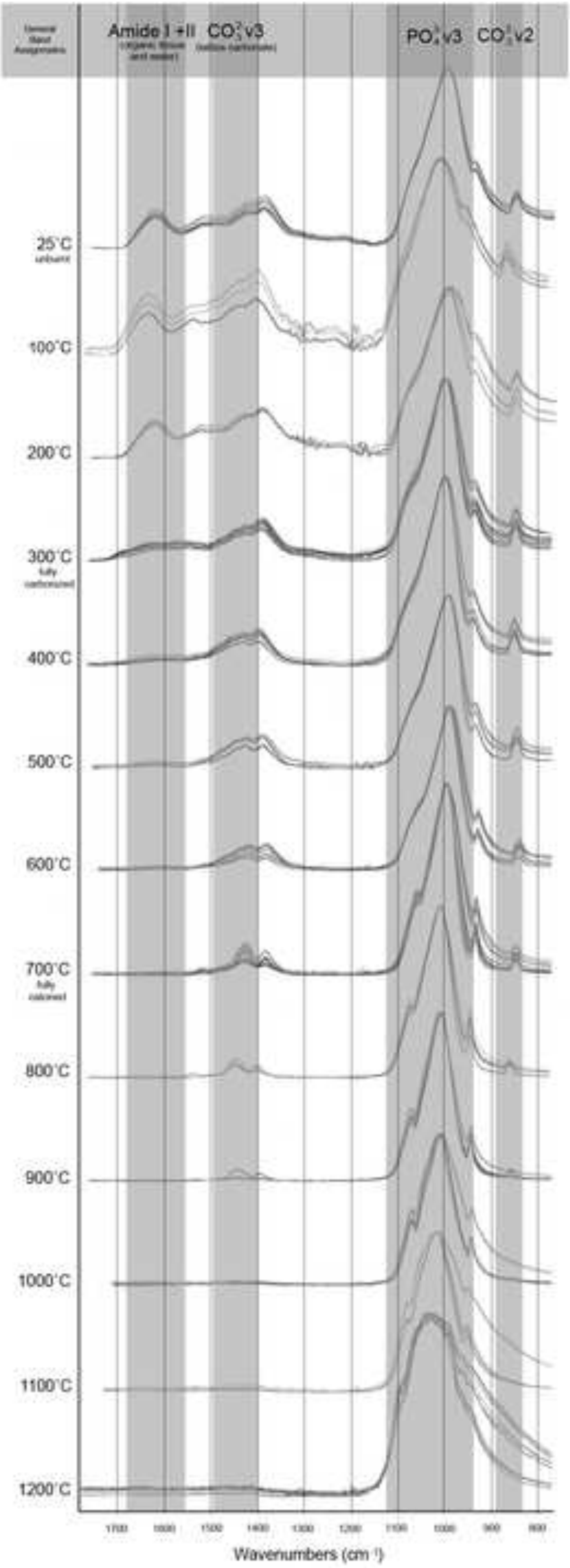

Figure
Click here to access/download;Figure;Fig 3.tif

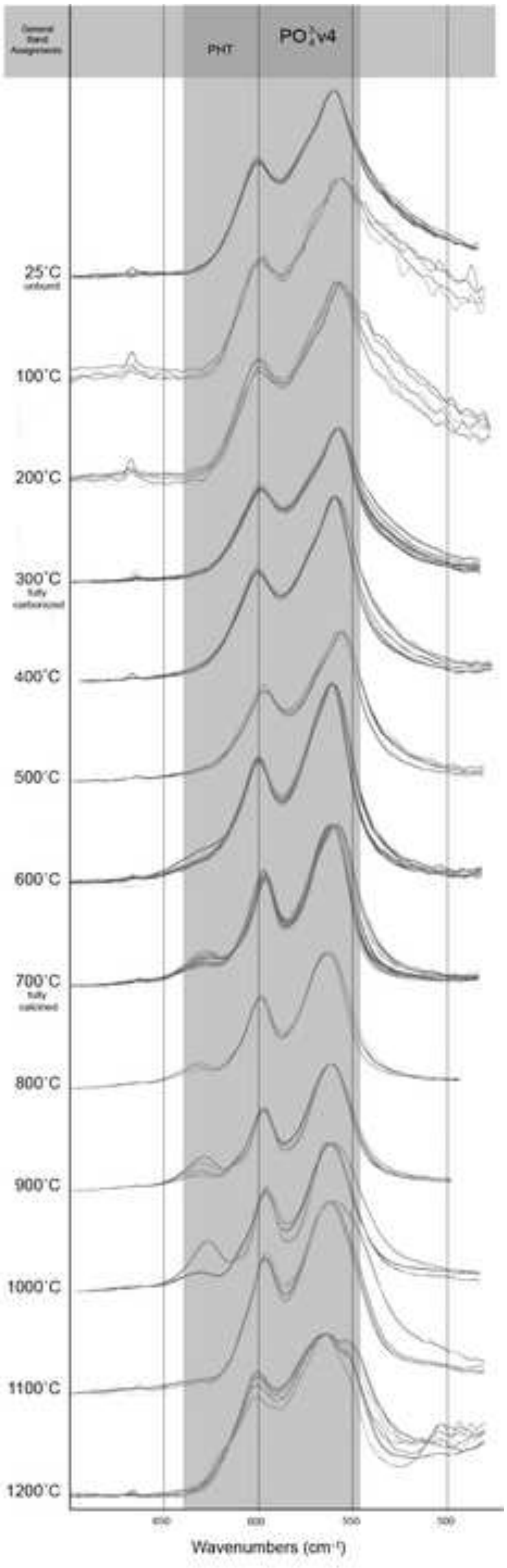

Figure Click here to access/download;Figure;Fig 4.tif

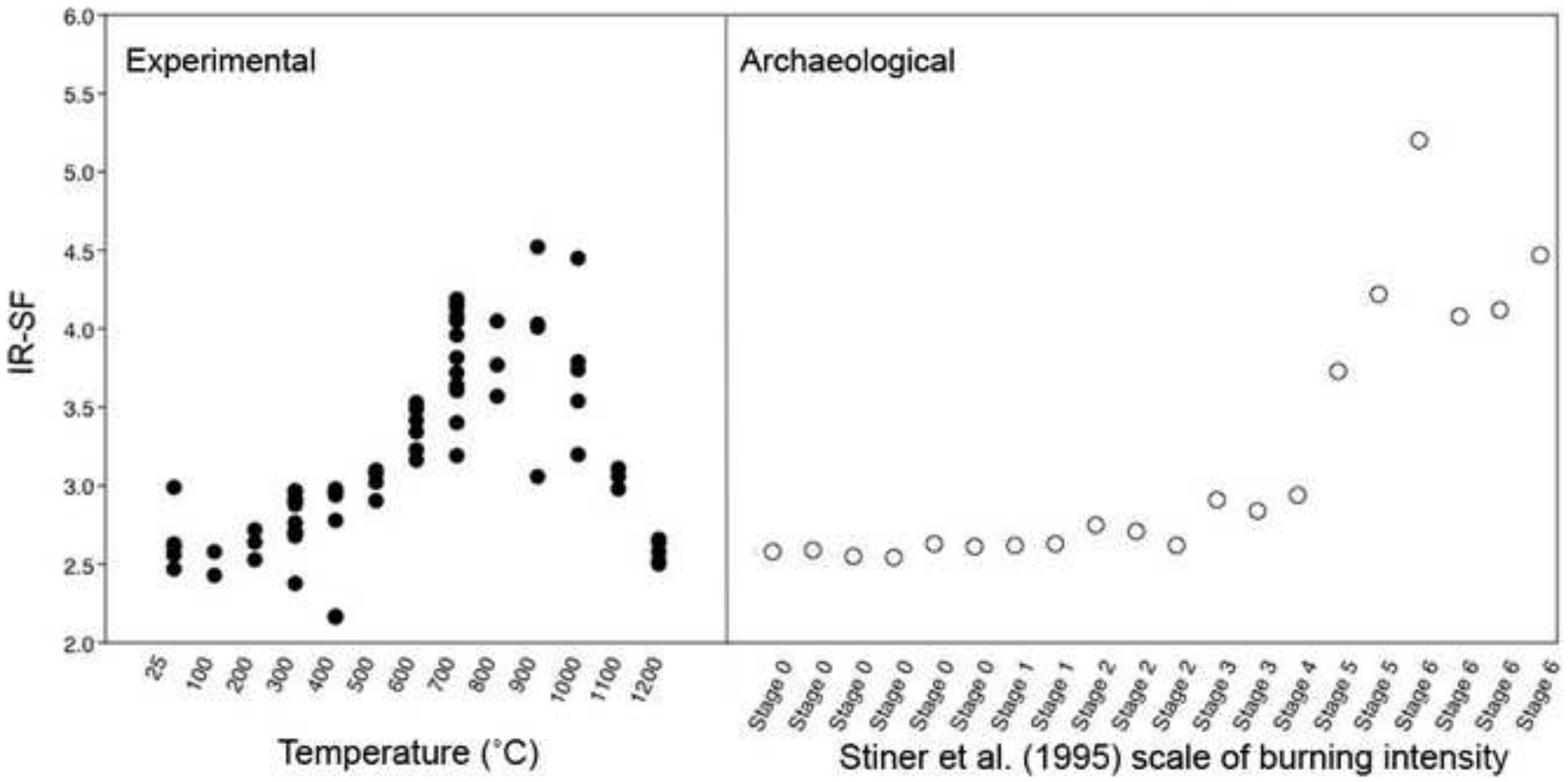

Figure

Click here to access/download;Figure;Fig 5.tif

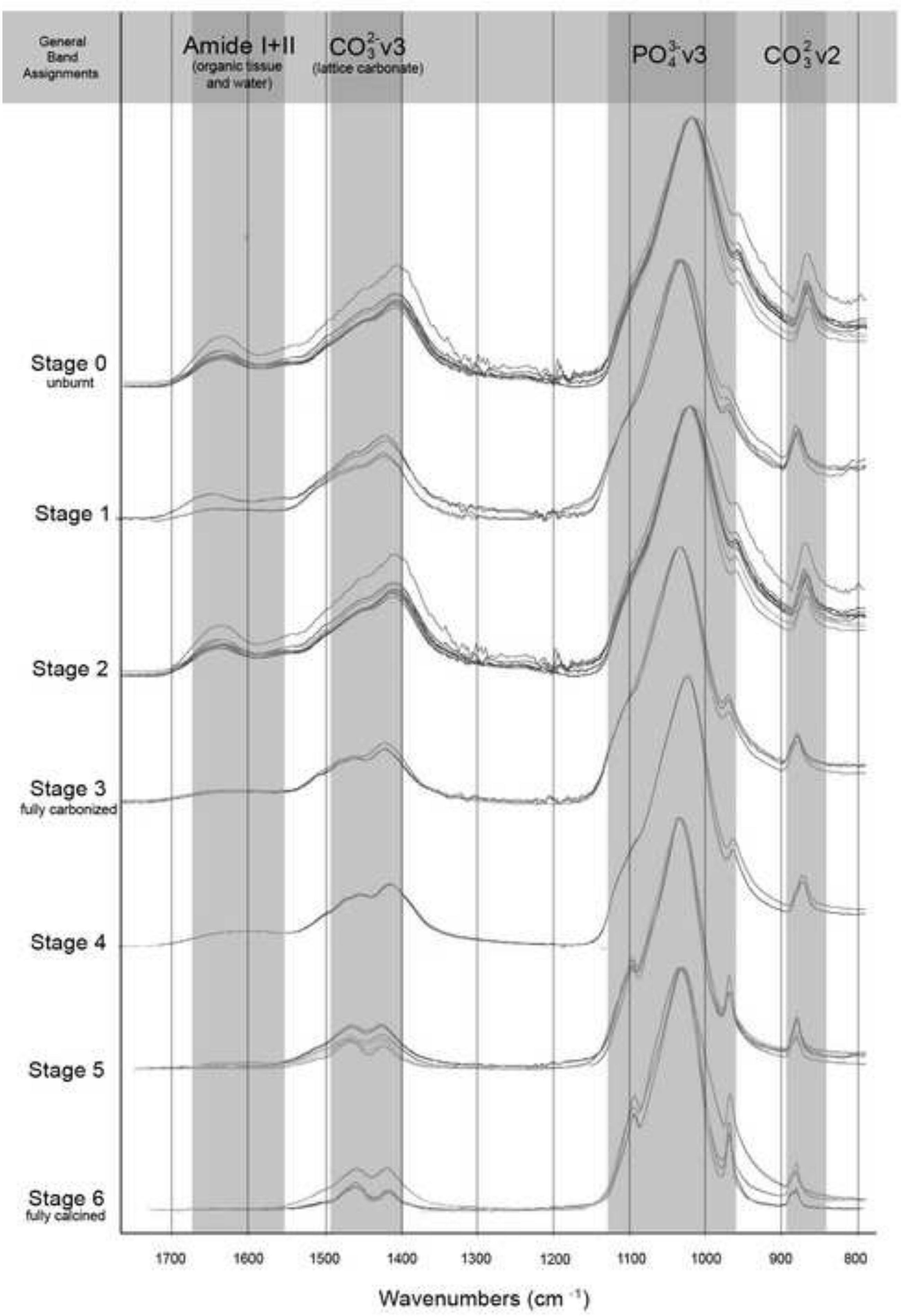

Figure

Click here to access/download;Figure;Fig 6.tif

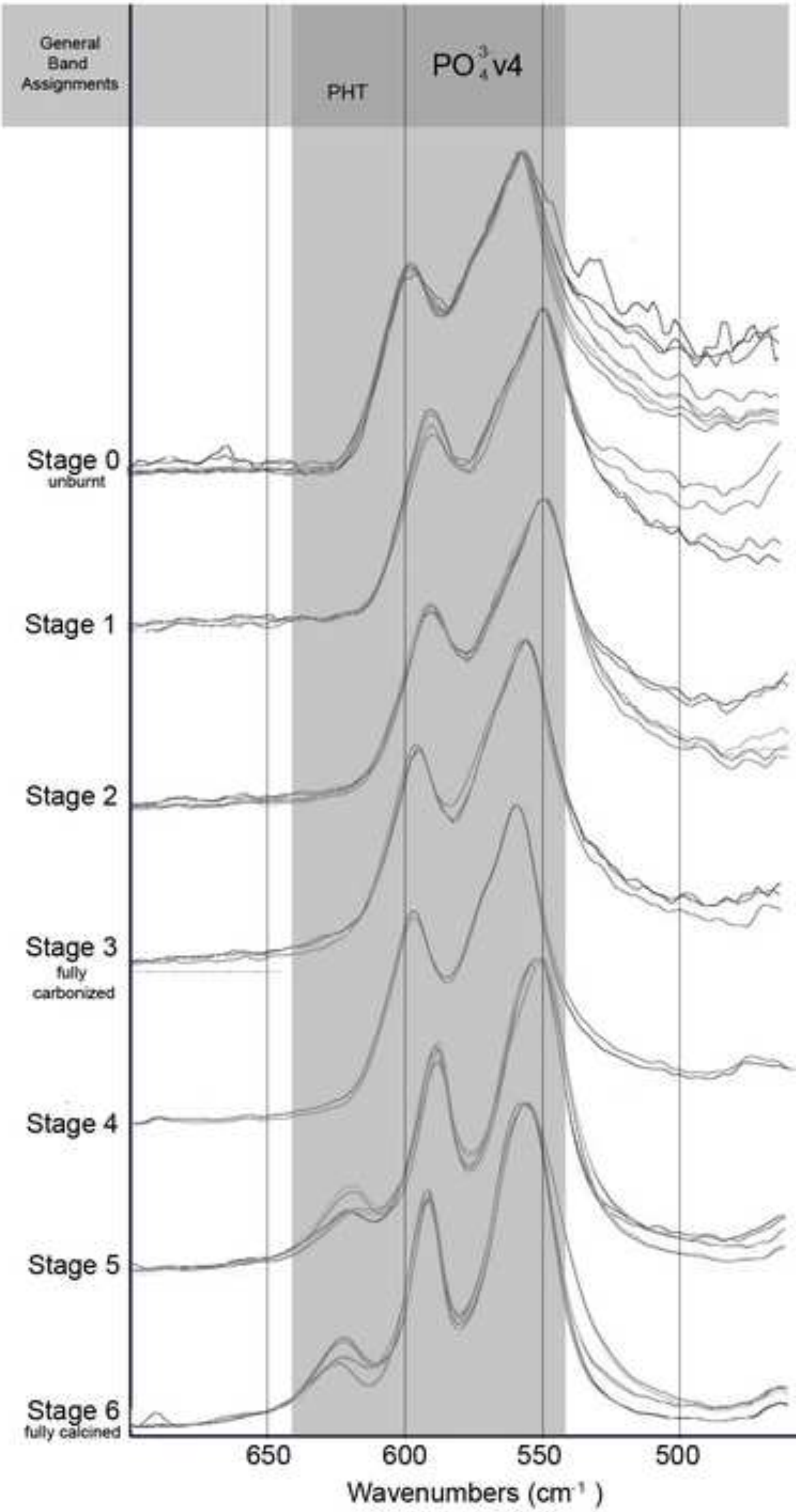

Click here to access/download;Figure;Fig 7.tif

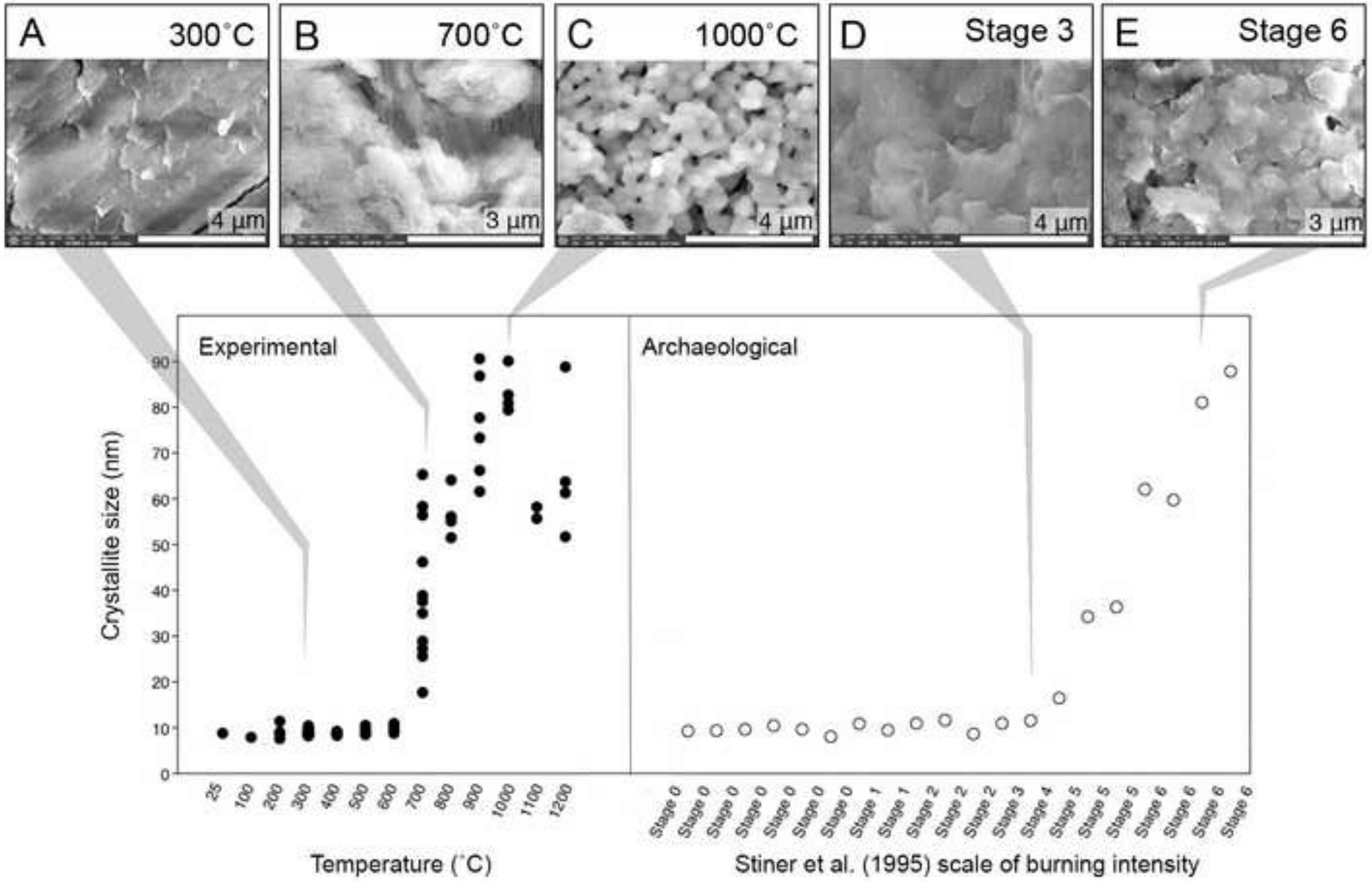

Click here to access/download
Supporting Information
S1 Table.pdf

Click here to access/download
Supporting Information
S2 Table.pdf

Click here to access/download
Supporting Information
S3 Table.pdf

Click here to access/download
Supporting Information
S4 Table.pdf

Click here to access/download
Supporting Information
S1 Appendix.pdf

Revised Manuscript with Track Changes

Long Title: Characterization of structural changes in modern and archaeological burnt bone: Implications for differential preservation bias
Short Title: Crystallite size growth in modern and archaeological burnt bone

~~**Authors:** Giulia Gallo[1,2*¶], Matthew Fyhrie[1¶], Cleantha Paine[3,4¶], Sergey V. Ushakov[5¶], Masami Izuho[6&], Gunchinsuren Byambaa[7,8&], Nicolas Zwyns[1,2,9¶], Alexandra Navrotsky[5&]~~

Authors: Giulia Gallo[1,2*], Matthew Fyhrie[1], Cleantha Paine[3], Sergey V. Ushakov[4], Masami Izuho[5], Byambaa Gunchinsuren[6], Nicolas Zwyns[1,2,7], Alexandra Navrotsky[4]

Affiliations:
1- Department of Anthropology, University of California Davis, 95616, Davis CA, USA
2- Center for Experimental Archaeology at Davis, University of California Davis, 95616, Davis CA, USA
~~3- Department of Archaeology, University of Cambridge, Cambridge, UK~~
~~4- Archaeology Institute University of the Highlands and Islands, Kirkwall, UK~~
~~5- School of Molecular Sciences and Center for Materials of the Universe, Arizona State University, 85287, Tempe AZ, USA~~
~~6- Tokyo Metropolitan University, Tokyo, Japan~~
~~7- Institute for History and Archaeology, Mongolia Academy of Science, Ulaanbaatar, Mongolia~~
~~8- Academy of Sciences, Mongolia~~
~~9- Department of Human Evolution, Max Planck Institute for Evolutionary Anthropology, 04103, Leipzig, Germany.~~

-1- Department of Anthropology, University of California Davis, 95616, Davis CA, USA
2- Center for Experimental Archaeology at Davis, University of California Davis, 95616, Davis CA, USA
3- Archaeology Institute University of the Highlands and Islands, Kirkwall, UK
4- School of Molecular Sciences and Center for Materials of the Universe, Arizona State University, 85287, Tempe AZ, USA
5- Tokyo Metropolitan University, Tokyo, 192-0397, Japan
6- Institute for History and Archaeology, Mongolia Academy of Science, Khukov Street-77, Ulaanbaatar, Mongolia
7- Department of Human Evolution, Max Planck Institute for Evolutionary Anthropology, 04103, Leipzig, Germany

*Corresponding author
Email: gtgallo@ucdavis.edu

[¶] These authors contributed equally to this work
[&]These authors contributed equally to this work

Abstract:

Formatted: Line spacing: single

Formatted: Line spacing: single

Formatted: Line spacing: single

Structural and thermodynamic factors which influence burnt bone survivorship in archaeological contexts have not been fully described. A highly controlled experimental reference collection of fresh, modern bone burned in temperature increments 100-1200˚C is presented here to document the changes to bone tissue relevant to preservation using Fourier transform infrared spectroscopy and X-ray diffraction. Specific parameters investigated here include the rate of organic loss, amount of bone mineral recrystallization, and average growth in bone mineral crystallite size. An archaeological assemblage ca. 30,000 years ago is additionally considered to confirm visibility of changes seen in the modern reference sample and relate changes to commonly used zooarchaeological scales of burning intensity. The timing of our results indicates that the loss of organic components in both modern and archaeological bone burnt to temperatures up to 700˚C are not accompanied by growth changes in the average crystallite size of bone mineral bioapatite, leaving the small and reactive bioapatite crystals of charred and carbonized bone exposed to diagenetic agents in depositional contexts. For bones burnt to temperatures of 700˚C and above, two major increases in average crystallite size are noted which effectively decrease the available surface area of bone mineral crystals, decreasing reactivity and offering greater thermodynamic stability despite the mechanical fragility of calcined bone.

The degree of alteration to the crystallinity, porosity, and organic content of burnt bone is dependent on the intensity of burning. The influence of the burial environment on bone preservation is partly a consequence of extent of these changes. Bones which are vulnerable to aqueous dissolution are of particular interest to this study, as groundwater interaction is demonstrated to highly impact the preservation of unburnt bone and has not yet been addressed for bone burnt to different temperatures. Here we describe the timing of changing structural and compositional qualities of experimentally burnt bone (100-1200˚C in oxygen atmospheres) alongside an open-air archaeological assemblage of burnt bone from a fluctuating hydraulic environment in northern Mongolia dating back to 30 ka. Trends in crystallite growth and loss of organics are described with Fourier transform infrared spectroscopy with attenuated total reflectance attachment (FTIR-ATR) as well as X-Ray Diffraction (XRD) and demonstrate the vulnerabilities of charred and carbonized bone to groundwater movement. Organic protection by collagen or large bioapatite crystal sizes are found to likely have a strong influence on bone preservation, describing a potential bias in archaeological assemblages of burnt bone.

Introduction:

Anthropogenic burnt bones can be indicative of many social and economic behaviors and can contribute to studies identifying evidence for ritual activity [1], cremations [2-4], bone fuel [5-9], hygienic practices [5,9], cooking and marrow warming [10], and locations of combustion features [11]. Burnt bones undergo substantive structural and compositional changes at different burning intensities, however, and the implications of these changes for differential bone survivorship in an archaeological fauna assemblage is of critical importance for studies utilizing burnt material.

Formatted: Indent: First line: 0.5"

Bone diagenesis can result in bone mineral disintegration or dissolution [12]. The rate, sequence, and extent of diagenetic processes are determined by many factors, including the nature of the depositional environment and the age, element, and species of the bone tissue. Postmortem bone preservation is well described in regards to the differences between compact and cancellous bone [13-16], juvenile and adult bone [17,18], intra- and interspecies variation in bone size and density [19-21], and different environmental conditions [12, 22-26]. However, zooarchaeological evaluations of burnt bone preservation have resulted in varied interpretations

and have not addressed the different structural properties of bone burnt to different temperatures [5, 27].

The aim of this study is to describe the range of structural modifications to bone mineral produced by burning at different temperature intensities, and to relate such changes to standardized scales utilized in zooarchaeological methods, specifically here the Stiner et al. [27] scale of burning intensity. This is done with the intention of describing differences between categories of burnt bone, including any vulnerabilities to diagenetic processes that could result in assemblage biases within burnt bone in archaeological contexts. Here we present the results of a controlled experimental reference library of fresh, modern bone burnt in increments of 100-1200˚C and analyzed with Fourier transform infrared spectroscopy (FTIR) with Attenuated Total Reflectance (ATR) attachment and X-ray diffraction (XRD). We additionally compare spectroscopic measurements to a sample of burnt fauna dated to ca. 30 ka to verify visibility of alterations in an archaeological assemblage.

Formatted: Highlight

The extrinsic and intrinsic variables which affect bone preservation have been studied with regard to the impact of differences between compact and cancellous bone [1-4], juvenile and adult bone [5, 6], intra- and interspecies variation in bone size and density [7-9] and environmental conditions [10-15]. Evaluations of the taphonomic biases impacting the preservation of burnt bone have resulted in varied interpretations [16]. For example, highly burnt calcined bone has been suggested to be both exceedingly mechanically fragile as well as capable of surviving in non-ideal conditions [17]. To date, the properties of burnt bone which may explain or inform these assumptions have not been directly addressed [17]. It is necessary to explore such relationships, as burnt bone is a valuable resource contributing to the study of ancient fire residue [17-20].

Formatted: Line spacing: single

Bones burned by humans can be indicative of many social and economic behaviors and can contribute to archaeological studies identifying evidence for cremations [21, 22], bone fuel [16, 23-27], hygienic practices [16, 25], cooking and marrow warming [26], and locations of anthropogenic fire [20]. The thermal alteration of bone, therefore, has been the focus of many studies, with early investigations focusing on macroscopic changes in bone color and texture [17, 28]. This has led to the development of common-use zooarchaeological standards in codifying burnt archaeological bone assemblages, such as the Stiner et al. [17] scale of burning (Table 1). While it is recognized that bones burnt to different temperatures have different structural properties on the nano-, micro- and macro-scales, this variation within burnt fauna has not been addressed regarding biases in preservation nor investigated within an archaeological assemblage of burnt bone [17, 29]

Formatted: Indent: First line: 0", Line spacing: single

Formatted: Line spacing: single

Table 1: Burning intensity stages based on visual characterizations following Stiner et al. [17]

Burning Stage	Description
0	Not burnt
1	Slightly burnt, < 50% carbonized
2	Majority burnt, > 50% carbonized
3	Fully carbonized
4	Slightly highly burnt, < 50% calcined
5	Majority highly burnt, > 50% calcined

Formatted: Left

Formatted: Left

Formatted: Left

Formatted: Left

Formatted: Left

Formatted: Left

Formatted: Left

6	Fully calcined

The aim of this study is to review the modifications to bone related to burning and to specifically characterize the heat induced growth of bone crystals and loss of organics that may influence the susceptibility of burnt bone to aqueous dissolution. These structural changes and the potential biases in preservation are described here within a modern assemblage of experimentally burnt bone as well as within an archaeological assemblage of burnt fauna which has a depositional history of fluctuating hydraulic regimes in a geographic area without abundant documentation of ancient fire. This integrated approach incorporating both a thermodynamic perspective of bone and inferences about taphonomic history is necessary to enhance our understanding of the links between zooarchaeological evidence of fire, the impact of the depositional environment on osseous material, and burnt bone survival and visibility.

Formatted: Left

Formatted: Line spacing: single

Formatted: Indent: First line: 0", Line spacing: single

Background:

Bone

Formatted: Line spacing: single

Bone is comprised of organic proteins primarily of collagen, inorganic mineral, and water, creating a composite material organized in compact bone in cylindrical structures of concentric lamellae surrounding an interior channel for a central blood canal [28,29]. This hierarchical arrangement provides and maintains the biological roles of skeletal tissue: mechanical strength to transmit force and protect organs, and the regulation of homeostasis through ionic regulation [28-31].

Living bone is very porous, with around 12% of bone volume comprised of open spaces [2]. The concentric systems, known as Haversian systems, constitute a large percentage of the bone matrix porosity, with the remainder composed of resorption bays and voids created between the organic and inorganic components [29-32]. The amount, size, and density of pores in bone is variable across elements, species, and ages, although trabecular bone does exhibit a higher porosity than compact bone due to its more open structure [21, 29, 32].

Inorganic bone constituent, bioapatite, is isostructural to mineral hydroxyapatite $Ca_5(PO_4)_3OH$. The specific chemical compositions of bioapatite reflect diet, biological age through history of bone remodeling, and variation can exist both within species and within the skeletal elements themselves [29]. Bioapatite contains 5-8 wt% carbonate which can substitute either phosphate or hydroxyl group in hydroxyapatite structure [33]. Bioapatite has a high degree of nonstoichiometry, and its composition can be described as $Ca_{10-x}(PO_4)_{6-x}(HPO_4, CO_3)x(OH,1/2CO_3)_{2-x}$ with 0<x<2 [31, 34, 35].

In vivo bioapatite has extremely small, thin, plate-like morphologies (1-7 nm thick, 15-200 nm in length, and 10-80 nm in width) which are cross-linked to organic collagen fibrils [34-36]. Water is found in bone as loose mobile water in the extracellular matrix, in void spaces to facilitate movement, and integrated within and around the organic and mineral components [31, 37]. Bioapatite crystallites have typical surface areas above 200 m^2g and are heavily hydrated [30, 31, 35-38]. These surface layers of ions play a key role in the regulation of homeostasis, as they can be easily exchanged and provide a necessary capacity to regulate ionic concentrations in living tissue [3, 28, 38-41].

Bone Diagenesis

Diagenesis, the postmortem changes to bone tissue in burial environments, includes the integrated processes of microbial attack, water activity, and mineral recrystallization and can

result in the complete disintegration of bone material [12]. The arrangement and size distribution of pores at the time of burial are large predictors of bone decay or bone survival, as pores mediate the access and extent of destructive agents such as bacteria and water [18, 22-24, 42-44]. Microbial attack itself is an active and immediate process accounting for a large amount of initial organic destruction, especially in warmer environments [42]. Microbial access to collagen degrades the protein chains, effectively removing the organic component of bone [19, 24, 32, 42, 44, 45]. The removal of the collagen component results in a more brittle biomaterial on the macroscale, and leaves bioapatite crystals unprotected on the micro- and nanoscale [46].

Exposed bioapatite is vulnerable to the incorporation of impurities and to disintegration, as postmortem crystals initially retain the specific morphology, reactivity, and thermodynamic instability of living bioapatite [47, 48]. The reaction between bone mineral and water is the most significant predicate of bioapatite disintegration at this stage of diagenesis, and bones buried in environments with active water movement are highly vulnerable to leeching and dissolution, noted to be heightened when bioapatite is exposed and easily accessible after organic removal [24, 48]. There is no universal thermodynamic model of bioapatite solubility due to the complexity of bone as a biomaterial, and rather each crystal domain is assigned its own Metastable Equilibium Solubility (MES): a distribution phenomenon dependent on aspects of bone quality such as carbonate substitutions, ion vacancies, low crystallinity, and small crystal sizes [48, 49]. Uptake of contamination from the burial environment, such as rare earth elements and secondary calcite, has been noted to reduce as bone mineral spontaneously recrystallizes without in-vivo regulation and larger crystals grow at the expense of smaller crystals [18, 50, 51]. This process results in bone mineral with a slightly higher crystallinity, effectively decreasing the available reactive surface area of bioapatite and therefore the overall solubility compared to fresh bone [18, 36, 50, 51].

Formatted: Line spacing: single

Bone is a composite material comprised of inorganic compounds (primarily hydroxyapatite), organics (primarily collagen) and water [30, 31]. These biomaterials are hierarchically arranged to provide the necessary mechanisms and structures that maintain the biological role of skeletal tissue: mechanical strength to transmit force and protect organs, and the regulation of homeostasis through ionic regulation [32, 33].

Bone mineral, or, bioapatite, is nonstoichiometric calcium phosphate apatite, $Ca_{10-x}(PO_4)_{6-x}(HPO_4,CO_3)_x(OH,1/2CO_3)_{2-x}$ with $0 \leq x \leq 2$ [32, 34]. The specific chemical compositions of bioapatite reflect diet, biological age, bone remodeling, and can vary within individuals and within skeletal elements themselves [33]. Compared to hydroxyapatite, bioapatite composition and microstructure are characterized by a higher degree of nonstoichiometry, vacancies in the crystal lattice, nano-sized platelet shapes, and by the presence of a hydrated ionic layer [32, 35]. Nonstoichiometry in bioapatite is related to a low degree of crystallinity, in that the minerals are poorly ordered with various degrees of strain introduced by different sized crystallites and carbonate substitutions [32, 34, 36]. Type-A (CO_3 for OH) and the more prevalent Type-B (CO_3 for PO_4) are the most common substitutions in the crystal lattice and comprise approximately 5-8% of bone by weight [35].

In vivo bioapatite has extremely small, thin, plate-like morphology (1-7 nm thick, 15-200 nm length, and 10-80 nm width) and is cross-linked to collagen fibrils [34, 36, 37]. Water is found in bone as loose mobile water in the extracellular matrix and in void spaces to facilitate movement between collagen fibrils and minerals, as well as both incorporated within and around

the organic and mineral components [32, 38]. Bioapatite specifically features water in a hydrated ionic layer around the crystalline core and in vacancies in the crystal lattice [32, 38]. This hydrated layer regulates homeostasis, as the surface layer of ions can be easily exchanged with the large, specific, and very reactive surface of the bone mineral, estimated at 240 m^2g [31, 36, 37, 39]. This is necessary to regulate ionic concentrations [22, 27, 28, 30]. The high surface to mass ratio and reactive hydrated layer of the small crystallites results in an exchange capacity with large sorption coefficients on the order of 10^6-10^8 [39-41].

Porosity

Bioapatite, collagen, and water molecules are organized on the microscale into Haversian systems: cylindrical structures of concentric lamellae surrounding an interior channel for a central blood canal [30, 33]. In addition to the Haversian canal, two other features constitute the typical porosity inherent in bone matrix: the lacunal-canaculi network, also known as resorption bays (Howship's lacunae), which are large pores for osteoblast/osteoclast remodeling, and the smaller spaces created between collagen and apatite (collagen apatite porosity, also known as CAP) [33, 42]. Living bone is very porous, with around 12 % of bone volume comprised of pores [12]. The amount, size, and density of pores in compact bone is variable across and within elements, species, and age [33, 42]. Studies specifically addressing the mineral density of bone across species with computer tomography (CT) imaging have found that bone density is less variable between taxa than within taxa once differences in health or age of the animal are taken into account [8, 9]. With aging, mature bone experiences a decrease in mineral density, but immature bone exhibits overall the highest macro- and micro- porosity [6].

Diagenesis and Solubility

Diagenesis, the changes to bony tissue in burial environments, includes the integrated reactions of hydrolysis, microbial attack, and mineral recrystallization [15]. The rate and sequence of these processes are determined by many factors, including the nature of the depositional environment, and the age, element, and species of bone in life. Early taphonomic history and the initial arrangement and size distribution of the pores are particularly important for diagenetic processes, as higher degrees of porosity decrease protection and allow greater access to digenetic agents [6, 10-12, 43-45]. The extent of porosity inherent in a bone therefore has a large impact on the degree and rate of either bone decay or bone survival [12].

Other influential factors include the degree of flesh cover, groundwater interaction, and soil pH [6, 10-12, 15, 43-45]. Microbial attack is an active and immediate process especially for fleshed elements, accounting for a large amount of organic destruction [42, 45]. This is found to be especially true in warmer environments [43, 46]. Bacterial action also is more pronounced in fleshed burials, and lowers the bone pH, which fosters beneficial conditions for later mineral recrystallization [43].

The specific interaction between hydrologic conditions and bone is hypothesized to be the most significant predicate of bioapatite dissolution [12, 15, 37]. Hydrolysis is a reaction with water that results in chemical disintegration, typically an early stage of diagenesis [12, 47]. The mechanisms of hydrolysis depend on the relationship between the mineral and organic components of bone and the available access to pores, as well as the water fluctuations in the depositional environment. Three hydrological regimes have been outlined to address the impact water can have on bone: diffusion, recharge, and hydraulic flow [12, 45]. For diffusion, a waterlogged environment with negligible water movement, the dissolution process is slowed,

aiding bone preservation [12, 45]. For recharge regimes, in which the relative amounts of water changes, and for flow regimes, which are categorized as environments with water movement, there is a much higher potential for bone dissolution as water acts as an agent to dissolve and leach bone mineral and organics [12, 15, 45].

Bone dissolution is dependent on the loss of organic protection and the nature of the structure and surface characteristics of bone apatite [37]. Type I collagen itself is insoluble due to hydrophobic interactions, the highly ordered arrangement with other collagen fibers, and cross-linked arrangements with bone mineral [15]. Access to collagen and rate of collagen removal is mediated by the amount and size of pores, which are made available by bacterial action and recrystallization of the bone mineral [12, 39, 43, 44, 47, 48]. After death, this microbial action and the degradation of the protein chains into smaller peptides, or, gelatinization, effectively eliminates the organic components of bone and therefore the protection of bone mineral [7, 12, 46, 47].

Bone mineral microstructure is thermodynamically unstable. Without *in vivo* regulation, larger bioapatite crystals begin to spontaneously increase in size and become more ordered at the expense of smaller crystals [6, 37, 47, 49]. This process, Ostwald ripening, decreases the available surface area of the bone mineral, therefore slowing the amount of chemical reactions and substitutions that can take place [6, 47]. This period of early diagenesis can rapidly incorporate impurities and substitutions post-mortem due to the high sorption and reactivity of bioapatite [50, 51]. The timing and degree of recrystallization is related to the composition of the crystal structures, the rate of organic loss, and the depositional environment [10-12].

As the organic material protects the mineral phase and recrystallization is not instantaneous, the removal of collagen leaves bioapatite vulnerable to dissolution. There is no universally accepted, consistent, single solubility behavior of bioapatite, as bone is a complex biomaterial [51]. The causes of this variability have been ascribed to the particle size of bioapatite, the imperfections of the crystal lattice, and the presence of substitutions and calcium phosphate phases [51]. In the absence of universal thermodynamic model or bioapatite solubility, each crystal domain of bone mineral assigned its own Metastable Equilibrium Solubility (MES), which is a distribution phenomenon dependent on many variables and influenced by the size of the minerals and the extent of their surface reactivity [51]. Several aspects of bone quality such as carbonate content, ion vacancies, low crystallinity, and nanometric crystal dimensions have an effect on the MES phenomenon and predict likely vulnerabilities [31, 51].

Carbonate incorporation, typically through substitutions for hydroxyl or phosphate groups in the structure, increases the solubility of bioapatite [31, 51]. The substitutions and nonstoichiometry introduced through ion vacancies create imperfections in the crystal lattice, which lower the crystalline order and introduce strain [31, 51]. This increases the dissolution tendency, therefore making the bioapatite with higher proportions of carbonate more susceptible to dissolution [32, 42, 51-53]. Apatite maturity, despite fluctuating values under the MES phenomenon, decreases solubility [31]. Changes in the highly reactive surface area either through shape or size in the plate-like bioapatite crystals can change the severity in susceptibility to diagenetic change.

Crystalline apatite and bioapatite are most soluble in acidic environments [12, 15, 54]. Bone exhibits low solubility and high survivorship in alkaline environments (pH > 7.5), except when such environments also have high carbon dioxide concentrations, which removes calcium ions and leaves bioapatite susceptible to demineralization [15].

Burnt Bone

Burning bone results in the decomposition of the organics and loss of water, as well as in massive changes to bioapatite crystal dimensions and structure. The extent and degree of these alterations are correlated to temperature and burning atmosphere, producing bones with different mechanical and thermodynamic properties dependent on the extent of burning. These micro- and nano- scale transformations have a notable impact on visible macroscopic changes to heat altered bone, including color changes, cracking, shrinkage, weight loss, and fragmentation [27, 41, 52-55]. Burnt bone coloration is generally correlated to burning intensity, and the ease of color identification has assisted in the proliferation and use of zooarchaeological scales of coding heat alteration, such as the Stiner et al. [27] classification of burning intensity (Table 1).

Table 1: Burning intensity scale based on macroscopic visual qualities following Stiner et al. [27]

Burning Scale	Description
0	Not burnt
1	Slightly burnt, < 50% carbonized
2	Majority burnt, > 50% carbonized
3	Fully carbonized
4	Slightly highly burnt, < 50% calcined
5	Majority highly burnt, > 50% calcined
6	Fully calcined

Scales of burning based on macroscopic visual cues are tremendously beneficial for processing archaeological assemblages of burnt fauna, but do not reflect the sequence of changes in the composition and structural properties of burnt bone. Observations on the nano- and micro-scale have therefore led to the definition of four stages of burning which are correlated to the transformation of bone mineral and removal of organics on the nano- and microscale: dehydration, decomposition, inversion, and fusion [2, 54-57]. These stages are accomplished at different temperature thresholds and were defined in oxidizing burning conditions [2, 54-57]. The rate and degree of temperature induced changes depend on variables such as flesh coverage, heating and cooling rates and oxygen availability [54, 55]. Dehydration, or, the loss of water, occurs between 100 and 600˚C [2, 54, 56-58]. This wide temperature range likely accounts for the quicker loss of the loosely bound water between 25 and 250˚C and the eventual loss of the additional water more structurally bound to the mineral in temperatures above 100˚C [2, 31, 56, 57].

After initial dehydration, the second stage of bone combustion is organic decomposition, from 300 to 800˚C [2, 56, 57]. With collagen degradation starting at 112 - 260˚C, above 300˚C a large proportion of the organics is reduced to a char [58]. Between 300 and 500˚C most mass, 50 – 55 %, is lost, and above 500˚C any remaining char is removed by 700˚C [58]. The macroscopic transformation most noticeable with the decomposition stage is the striking changes in coloring,

with bone becoming visibly blackened with the charring of organics (300˚C), corresponding to Stages 1-3 of the Stiner et al. [27] scale, and after the complete removal of organics (700˚C) transitioning to a grey and chalky white hue for Stiner et al. [27] Stages 4-6 [2, 27, 54, 56, 57]. Bone that is blackened is referred to as combusted or carbonized dependent on burning atmosphere, while grey and white bone with all organics removed can be referenced as calcined [27, 54, 55].

Simultaneous to the loss of organics is the alteration of the bioapatite mineral, or the inversion stage, between 500 and 1100˚C [2, 56, 57]. With the removal of the organic component at 300˚C, the larger, plate-like crystals can spontaneously grow at the expense of smaller crystals [2, 56-58]. Experiments with bone burnt while powdered and subsequently cleaned with acetone report mean crystallite size increasing to 10 - 30 nm, and crystallite thickness moving from 2 to 9 nm [58, 59]. Above 500˚C, additional growth has been observed, with reported crystallite sizes plateauing at 110 nm and with crystal thickness reaching 10 nm [58]. The crystals, transforming from platelet like to hexagonal, later become equiaxed at 900˚C, growing more spheroidal with overall dimensions reaching 300 - 550 nm [59].

The last stage of heat alteration to bioapatite, fusion, accounts for the microstructural changes noted with the inversion phase above 700˚C [2, 56, 57]. Bone porosity initially increases from the originally porous *in vivo* status with the loss and charring of organics (~300˚C), which also corresponds to a loss in bone density [32, 59]. Carbonized and charred bone is reported to be most porous right before temperatures of calcination (600˚C) [32]. Beginning at 700˚C there is a densification as the bioapatite crystal grains grow, and by 900˚C there is a total structural coalescence from the additional crystal growth, resulting in an interlocking structure and a marked decrease in porosity [2, 58, 59].

These changes are all products of burning in oxidizing conditions [55]. If a bone is brought to temperatures greater than 300˚C without access to oxygen, a different pattern of thermal alteration has been demonstrated in controlled experiments [55]. When heating occurs in reducing atmospheres, the organic char is not removed and instead becomes more ordered [55]. The crystallinity of the bioapatite does increase, however, although at a slower rate than indicated in oxidizing conditions [55]. New compounds, such as cyanamide, are also likely formed around 600˚-700˚C [55]. Bones burnt in reducing atmospheres above 600˚C do not lose the organic char component, and therefore remain black in coloring [55].

Burnt Bone Diagenesis

The rapid morphological and compositional changes to burnt bone tissue are similar to changes seen over prolonged periods of time in the diagenesis of unburnt bone. This includes the removal of organic components and incorporated water, as well as the recrystallization of the bioapatite crystals. The immediate and greater extent of these changes in bone burnt to both low and high temperatures, however, results in a markedly different biomaterial at time of burial than unburnt bone.

Burnt bone is more fragile than unburnt bone, with fragmentation a function of burning intensity [27]. The dehydration and eventual complete removal of collagen from bone tissue significantly changes the toughness and strength properties of bone, altering the density, the structural integrity, and the stress and strain relationship [27, 60-62]. This ultimately results in a

greater likelihood of mechanical fracture correlated to the amount of collagen lost, leaving calcined bone the most mechanically vulnerable [27, 60]. Due to this extreme friability, recovered burnt bone fragments do not reflect initial size at deposition and processes such as burial and trampling can severely and easily fragment burnt bone [27, 63].

The fragility, likely presence of small fragment sizes, and elimination of organic components of bone burnt to lower temperatures provides greater surface area and easy access for diagenetic agents in the context of burial environments. Bone mineral does, however, undergo tremendous crystallite growth and reorganization with burning at higher temperatures, enabling calcined bone to be protected from contamination [54, 58, 64, 65]. Because of this, calcined bone is recognized to be the most reliable source of inorganic C14 for radiocarbon dating, as the elevated crystallinity that accompanies heat alteration at high temperatures protects the Type A and B carbonate substitutions and secondary carbonate incorporated from the burning atmosphere from further alteration, which subsequently can be used to date the burning event [64, 66].

Questions about the changing vulnerabilities of differentially burnt bone prompted our investigations into the characterization of structural changes of modern and archaeological bone burnt at different temperatures. Of specific interest to this study is the timing of the organic loss, and therefore loss of bioapatite protection, in reference to the increases in crystallinity and crystal sizes of bone mineral. Archaeological bone, both unburnt and burnt, is considered in this study as an actualistic reference to relate implications to commonly used zooarchaeological scales of burning intensity, and to monitor the extent of alterations related to the spontaneous postmortem recrystallization of bone mineral which occurs over time in burial environments.

Burning bone results in tremendous changes to bioapatite crystal dimensions and structure, as well as removing organic components and incorporated water. Notable macroscopic changes to bone which has undergone heat alteration includes color changes, cracking, shrinkage, weight loss, and fragmentation [17, 28, 55, 56]. These visible transformations are indicators of temperature and burning atmosphere and are dependent on modifications happening on the micro- and nanoscales [56-58]. Processes such as manganese staining and sun bleaching may also influence bone color and to avoid the misidentification of burnt bone, changes in bone mineral crystal size, morphology and microstructure have been used to verify thermal alteration [21, 29, 49, 57, 58].

Four stages of burning have been defined that are correlated to the incineration and eventual removal of organics and transformation of bone mineral: dehydration, decomposition, inversion, and fusion [21, 57, 58, 60, 61]. These stages are accomplished at different temperature thresholds and were defined in oxidizing burning conditions [21, 57, 58, 60, 61].
The rate and degree of temperature induced changes depend on variables such as flesh coverage, heating and cooling rates and oxygen availability [57, 58]. Dehydration, or, the loss of water, occurs between 100 and 600˚C [21, 29, 57, 60, 61]. This wide temperature range likely accounts for the quicker loss of the loosely bound water between 25 and 250˚C and the eventual loss of the additional water more structurally bound to the mineral above 100˚C [21, 32, 60, 61].
After initial dehydration, the second stage of bone combustion is organic decomposition, from 300 to 800˚C [21, 60, 61]. With collagen degradation starting at 112 - 260˚C, above 300˚C a large proportion of the organics is reduced to a char [29]. Between 300 and 500˚C most mass, 50 – 55 %, is lost, and above 500˚C any remaining char is removed by 700˚C [29]. The macroscopic transformation most noticeable with the decomposition stage are the striking changes in coloring,

Formatted: Highlight

Formatted: Line spacing: single

Formatted: Indent: First line: 0", Line spacing: single

with bone becoming visibly blackened with the charring of organics (300˚C) and after the complete removal of organics (700˚C) transitioning to a grey and chalk white hue [17, 21, 57, 60, 61]. Bone that is blackened is referred to as charred or carbonized, while grey and white bone with all organics removed can be referenced as calcined [17, 57].

Simultaneous to the loss of organics is the alteration of the bioapatite mineral, or the inversion stage, between 500 and 1100˚C [21, 60, 61]. With the removal of the organic component at 300˚C, the larger, plate-like crystals can spontaneously grow at the expense of smaller crystals [21, 29, 60, 61]. Experiments with bone burnt while powdered and subsequently cleaned with acetone report mean crystallite size increasing to 10 – 30 nm, and crystallite thickness moving from 2 to 9 nm [29, 60, 62]. Above 500˚C, additional growth has been observed, with reported crystallite sizes plateauing at 110 nm, with crystal thickness reaching 10 nm [29]. The hexagonal crystals later become equiaxed at 900˚C, growing more spheroidal, with overall dimensions reaching 300 – 550 nm [62].

The last stage of heat alteration to bioapatite, fusion, accounts for the microstructural changes noted with the inversion phase above 700˚C [21, 60, 61]. Bone porosity initially increases from *in vivo* status with the loss and charring of organics (~300˚C), corresponding to a loss in bone density [42, 62]. Charred bone is reported to be most porous right before temperatures of calcination (600˚C) [42]. Beginning at 700˚C there is a densification as the bioapatite crystal grains grow, and by 900˚C there is a total structural coalescence resulting in an interlocking structure and a marked decrease in porosity [29, 21, 62].

These changes are all products of burning in oxidizing conditions [58]. If a bone is brought to temperatures greater than 300˚C without access to oxygen, a different pattern of thermal alteration has been demonstrated in controlled experiments [58]. When heating occurs in reducing atmosphere, the organic char is not removed and instead becomes more ordered [58]. The crystallinity of the bioapatite does increase, however, although at a slower rate than indicated in oxidizing conditions [58]. New compounds, such as cyanamide, are also likely formed around 600˚-700˚C [58]. Bones burnt in reducing atmospheres above 600˚C do not lose the organic char component, and therefore remain black in coloring [58].

Formatted: Line spacing: single

The site of Tolbor-17

The Ikh-Tolborin-Gol is a low altitude pass on the western flank of the Khangai Mountains of Northern Mongolia. It is part of the Selenga drainage system, the main river feeding Lake Baikal (Fig. 1) [64]. This river valley preserves a wealth of Upper Paleolithic (UP) locales including Tolbor-4, Tolbor-15, Tolbor-16, and Tolbor-17 (T-17) [64-67]. Most of the sites document periodic human occupations starting with the Initial Upper Paleolithic, ca. 45 ka, until the Holocene. The latter has recently been dated with polymineral post-IR IRSL, Quartz OSL, and radiocarbon to 42.5-45.6 ka, establishing the timing for a movement of population between the Siberian Altai and Northwestern China, contemporaneous with the earliest *Homo sapiens* fossils in the region [64]. The following occupations in the valley are associated with the Upper Paleolithic (UP) in the broad sense and were made by *Homo sapiens*. Although it is often assumed that fire is part of the modern human behavioral repertoire allowing expansion into cold climates, evidence of the use of fire in the UP Tolbor locales is rare and has been only briefly reported [68, 69].

Tolbor-17 provides a rare opportunity to investigate burning within faunal remains that are usually poorly preserved in the region. Like most of the other locales, T-17 is an open-air environment with a fluctuating low energy run-off constituting a recharge water regime. Initially

excavated as a series of two test pits with dimensions 2m x 1m, the excavators at T-17 piece plotted all finds > 2 cm, and the remaining sediment from each bucket volume of excavated material was dry sieved with 4mm and 2 mm mesh screens, with all material subsequently sorted. The T-17 lithological Unit 3 is characterized by the presence of UP lithic artifacts and organic faunal preservation, despite sedimentary evidence for episodic sheet erosion, prolonged groundwater interaction, chemical weathering, and long surface exposure. Based on its geological setting, the material studied here belongs to the second half of the Marine Isotope Stage (MIS) 3, ca. 40–30 ka cal. BP. Unburnt and burnt fauna have been successfully recovered from Unit 3; however, this assemblage has been identified as extremely fragmentary and traditional zooarchaeological analyses considering butchery and taxon identification are still in the preliminary stages.

The inclusion of the faunal material from T-17 in this study is both for the purposes of providing a prehistoric archaeological assemblage for spectroscopic reference, as well as to specifically address the potential biases within this burnt portion of this faunal assemblage.

Materials and Methods:

Modern bone sample collection and preparation

A controlled experimental reference collection was created with modern bone to investigate the timing and impact of thermal alteration on organic loss, recrystallization indices, and crystallite size growth. Cortical bone from three cow femurs and two horse metacarpals from five different individuals were selected for this study. Cow femurs were procured the day after butchery from a local butcher and were never frozen. Flesh was scraped manually to prepare for drilling. Horse metacarpals were obtained postmortem from completed forelimb tissue collections of horses humanely euthanized for purposes other than this study at the UC Davis School of Veterinary Medicine. Metacarpals were simmered in water with the addition of borax to assist with defleshing, although bones remained greasy.

A diamond drill coring bit was used to produce solid plugs of cortical bone 3mm x 3mm, with weights ranging from 53.2 to 58.1 mg. Coring was constrained to the cortical bone tissue from the mid-diaphysis of both cow and horse bones. Solid bone plugs were specifically utilized in lieu of bone powder to avoid the effects of powder heating, as powder has an increased surface area and would be more reactive to thermal alteration. To fit the dimensions of crucibles used for thermal analyses, plugs were filed with diamond files. Post-experimental heating, three samples were selected for imaging with Scanning Electron (SE) microscopy for visualization purposes. All bone samples were then powdered with an agate mortar and pestle and sieved with 234 µm mesh.

Modern bone thermal analysis

The controlled annealing of modern bone samples was performed with Setaram Labsys Evo thermal analyzer. Bone core samples were placed in a 100 µl Al_2O_3 crucible and air flow 40 ml/min was established. The samples were brought to desired temperatures from 100 to 1200°C in 100˚C increments, with heating rate 20˚C /min and held isothermally for 30 minutes. The weight change and heat flow traces were recorded continuously and corrected for the baseline. Additional samples were produced at 300 and 700˚C with ramp 50°C /min and one hour dwell time for comparison.

Formatted: Font: (Default) Times New Roman, 12 pt

Formatted: Line spacing: single

Samples of archeological bones were characterized using Fourier transform infrared spectroscopy (FTIR) with an attenuated total reflectance (ATR) attachment, powder X-ray diffraction (XRD), and environmental scanning electron microscopy (eSEM). Burnt modern animal bone samples were annealed in a thermal analyzer to different temperatures and characterized using the same techniques to provide a controlled reference.

Archaeological case study sample collection and preparation

Archaeological unburnt and burnt bone samples were collected from the site of Tolbor-17, an open air locale on a low altitude pass on the western flank of the Khangai Mountains of Northern Mongolia. The Ikh-Tolborin-Gol is part of the Selenga drainage system, the main river feeding Lake Baikal (Fig. 1) [67]. This river valley preserves a wealth of Upper Paleolithic (UP) locales including Tolbor-4, Tolbor-15, Tolbor-16, and Tolbor-17 (T-17) [67-70]. Most of the sites document periodic human occupations starting with the Initial Upper Paleolithic, ca. 45 ka, until the Holocene. The latter has recently been dated with polymineral post-IR IRSL, Quartz OSL, and radiocarbon to 42.5-45.6 ka, establishing the timing for a movement of population between the Siberian Altai and Northwestern China, contemporaneous with the earliest *Homo sapiens* fossils in the region [67]. The following occupations in the valley are most likely associated with *Homo sapiens*, and are characterized as Upper Paleolithic (UP) in the broad sense. Although it is often assumed that fire is part of the modern human behavioral repertoire allowing expansion into cold climates, evidence of the use of fire in the UP Tolbor locales is rare and has been only briefly reported [71, 72].

Tolbor-17 provides a rare opportunity to investigate faunal remains, as organic material is usually poorly preserved in the region and burnt fauna has not yet been described in detail. Like most of the other locales, T-17 is an open-air environment with a fluctuating low energy run-off, constituting a fluctuating recharge water regime [24, 44]. Initially excavated as a series of two test pits with dimensions 2 m x 1 m, the excavators at T-17 piece plotted all finds > 2 cm, and the remaining sediment from each bucket volume of excavated material was dry sieved with 4 mm and 2 mm mesh screens, with all material subsequently sorted. The T-17 lithological Unit 3 is characterized by the presence of UP lithic artifacts and organic faunal preservation, despite sedimentary evidence for episodic sheet erosion, prolonged groundwater interaction, chemical weathering, and long surface exposure. Based on its geological setting, the material studied here belongs to the second half of the Marine Isotope Stage (MIS) 3, ca. 40 -30 ka cal. BP and is described as UP. Unburnt and burnt fauna have been successfully recovered from Unit 3; however, this assemblage is extremely fragmentary and traditional zooarchaeological analyses based on taxonomic identification and prey selection and processing are still in the preliminary stages.

Mapped (> 2 cm) and screened (< 2 cm-2 mm) faunal remains from the T-17 UP assemblage were cleaned, sorted following the Stiner et al. [27] seven stage visual scale of burning intensity, and weighed (Table 2). No burning was noted in bone > 2 cm except for a single fragment, but fauna < 2 cm- 2mm recovered in the screened material is found to span all stages of burning intensity within Unit 3 of the exposed excavation surface (Table 2). Of the burnt fauna, a large percentage is nearly or fully calcined, a notable observation due to the recognized mechanical fragility of calcined bone and the unprotected open-air environment of T-17. All excavated fauna was assigned burning stages following Stiner et al. [27] and 20 bones from the same test pit of the Unit 3 assemblage were sub-sampled. A minimum of one category representing bones from this sample were selected for subsequent spectroscopic analyses to

Formatted: Highlight

Formatted: Highlight

Formatted: Highlight

confirm heat alteration and investigate organic composition, crystallinity, and crystallite size. Bones were cleaned with ionic water sonication, and two samples representing before and after calcination were selected for imaging with SE microscopy prior to all samples being powdered with a diamond file and an agate mortar and pestle. All archaeological bone powder samples were then sieved with 234 µm mesh. No permits were required for the described study, which complied with all relevant regulations.

Table 2: T17 Unit 3 fauna summary with burning stages following Stiner et al.[27].

-	Screened (< 2 cm)	Piece plotted (>2 cm)	Total
Burning Stage	Weight (g)	Weight (g)	Weight (g)
0	141.36	213.31	354.47
1	1.04	0	1.04
2	5.17	1.01	6.18
3	0.69	0	0.69
4	1.95	0	1.95
5	1.37	0	1.37
6	4.5	0	4.5

Formatted: Line spacing: single

Archaeological bone sample collection and preparation

Mapped (> 2 cm) and screened (< 2 cm-2 mm) faunal remains from the T17 UP assemblage were cleaned, weighed in milligrams, and sorted following the Stiner et al. [17] seven stage visual scale of burning intensity (Table 2). No burning was noted in bone > 2 cm except for a single fragment, but fauna < 2 cm-2mm recovered in the screened material is found to span all stages of burning intensity within Unit 3 of the exposed excavation surface (Table 2). Of the burnt fauna, a large percentage is nearly or fully calcined, a notable observation due to the described mechanical fragility of calcined bone and unprotected open-air environment. 20 bones from the Unit 3 assemblage, representing each burning category and from the same meter square, were sampled for subsequent spectroscopic analyses to confirm heat alteration and investigate crystallite size. Bones were cleaned with ionic water sonication, and bone powder was produced using a diamond file and an agate mortar and pestle. All archaeological bone powder samples were sieved with 234 µm mesh.

Table 2: T17 Unit 3 fauna summary with burning stages following Stiner et al.[17].

-	Screened (< 2 cm)	Piece plotted (>2 cm)	Total
Burning Stage	Weight (mg)	Weight (mg)	Weight (mg)
0	141.36	213.31	354.47
1	1.04	0	1.04
2	5.17	1.01	6.18
3	0.69	0	0.69
4	1.95	0	1.95
5	1.37	0	1.37

6	4.5	0	4.5

Formatted: Line spacing: single

Modern bone sample collection and preparation

A controlled experimental reference collection was created with modern bone to investigate the impact of thermal alteration on crystallite size and loss of organics. Cortical bone from three cow femurs and two horse metacarpals from five different individuals were selected for this study. Cow femurs were procured the day after butchery and were never frozen. Flesh was scraped manually to prepare for drilling. Horse metacarpals were also procured fresh, but simmered in water with the addition of borax to clean, although bones remained greasy.

A diamond drill coring bit was used to produce solid plugs of cortical bone 3mm x 3mm, with weights ranging from 53.2 to 58.1 mg. Coring was constrained to the cortical bone tissue from the mid-diaphysis of both cow and horse bones. Solid bone plugs were specifically utilized in lieu of bone powder to avoid the effects of powder heating, as powder has an increased surface area and would be more reactive to thermal alteration. Subsequent to drilling, plugs were filed with a diamond file to fit the dimensions of crucibles used for thermal analysis

Formatted: Indent: First line: 0.5", Line spacing: single

Formatted: Line spacing: single

Thermal analysis of modern bone samples

The controlled annealing of modern bone samples was performed with Setaram Labsys Evo thermal analyzer. Bone core samples were placed in a 100 µl Al_2O_3 crucible and air flow 40 ml/min was established. The samples were brought to desired temperatures in 100 -1200°C range with heating rate 20˚C /min and held isothermally for 30 minutes. The weight change and heat flow traces were recorded continuously and corrected for the baseline. Additional samples were produced at 300 and 700˚C with ramp 50°C /min and one hour dwell time for comparison. Post heating, bone plug samples were powdered with an agate mortar and pestle and sieved with 234 µm mesh.

Infrared spectroscopy data collection and analysis

Infrared spectroscopy data collection and analysis

FTIR spectroscopy is a semi-quantitative method which characterizes bond vibrations, absorbed at specific wavelengths of transmitted incident light from infrared radiation, to identify compositional and structural properties of materials [54, 73]. When applied to bone, FTIR spectroscopy can yield valuable information regarding the presence and quality of preserved organic components, as well as the relative degree of structural order, size, and strain of bioapatite crystals [74-78]. This is particularly useful for the detection of organic preservation in samples screened prior to radiocarbon and stable isotope studies, and for diagenetic studies evaluating the integrity of bone mineral [77-78].

FTIR spectroscopy has additionally had success identifying thermally altered bone, as changes to bone composition and bioapatite crystallinity can be monitored though several heat induced peak transformations which cannot be mistaken for macroscopic staining or bleaching [4, 33, 53-55, 79, 80]. The identification of FTIR spectral peaks associated with the thermal alteration of bone has been extensively documented, with major alterations monitored through: (1) the ratio of carbonate to phosphate present in the sample, the C/P ratio, (2) the depletion of the presence of amide I and II functional groups, representing the organic components of bone, and (3) the presence of heat specific peak splitting, such as the loss of the peak at 874 cm^{-1} correlated to CO_3^{2-} v2 at temperatures over 1000˚C, and the PHT shoulder peak at temperatures

over 700˚C [54, 55, 57, 81-83]. Measures of the crystallinity of a sample can be inferred from the infrared splitting factor (IRSF), which extrapolates the changing size and order of bioapatite crystals through increase of splitting seen in the PO_4^{3-} v4 peaks [54, 57, 81].

Specific peaks relevant to this study and their inferred functional groups include the 1650 cm^{-1} and 1550 cm^{-1} peaks for the measurement of amide I and II, the 874 cm^{-1} and 1415 cm^{-1} peaks indicating presence of the v2 and v3 of carbonate, and the 900-1200 cm^{-1} and 50-600 cm^{-1} spectral regions related to the v3 and v4 phosphate components (Table 3). Additionally, the appearance of a 625cm^{-1} shoulder peak is attributed here to PO_4^{3-} v4 bending, known as the phosphate high temperature (PHT) [57].

A Nicolet 6700 Fourier transform infrared spectrometer with an ATR attachment and a deuterated triglycine sulfate (DTGS) detector and single bounce diamond crystal was used. The ATR method uses an attachment with a diamond or zinc crystal to produce spectra through the phenomenon of internal reflectance [84-86]. The application of ATR minimizes sample preparation, which in turn minimizes contamination [73, 82, 84]. Spectra were collected with 256 scans in the 4000 - 400 cm^{-1} frequency region and with an 8 mm spectral range. Each archaeological and modern bone powder sample was retested for quality control.

Eight peak measurements were monitored for 168 scans representing 84 individual samples for this study, 62 modern and 22 archaeological. Each sample was tested twice, and measurements presented here represent the average values of both scans. FTIR-ATR spectra were processed with OMNIC software.

Table 3: FTIR-ATR wavenumbers associated with likely functional groups relevant to this study and the thermal alteration of bone.

Wavenumber	Inferred peak assignment	Peak transformation relevant to this study
1630-1660 cm^{-1}	organic tissue and water, amide I + II	decrease and absence
1400-1550 cm^{-1}	CO_3^{2-} v3	1415 cm^{-1} peak a component of C/P ratio
1028-1100 cm^{-1}	PO_4^{3-} v3	1035 cm^{-1} peak a component of the C/P ratio
874 cm^{-1}	CO_3^{2-} v2	peak loss
565 cm^{-1}, 605 cm^{-1}	PO_4^{3-} v4	growth of 565 cm^{-1} and 605 cm^{-1} and decrease of the 595 cm^{-1} trough utilized for the infrared splitting factor (IRSF); phosphate high temperature (PHT) shoulder growth at 625 cm^{-1}

The IRSF measurements were procured for all samples following Weiner and Bar-Yosef [81].

$$\text{Infrared Splittng Factor:} \frac{(565\ \text{cm}^{-1}\ peak\ ht + 605\ \text{cm}^{-1}\ peak\ ht)}{595\ \text{cm}^{-1}\ \ peak\ ht}$$

An additional measure of the carbonate to phosphate content, the C/P ratio, was also determined for all samples. The C/P ratio decreases with burning and utilizes the 1035 cm^{-1} phosphate peak unaffected by IRSF changes [73, 82].

$$\frac{\mathrm{C}}{\mathrm{P}}\ \text{ratio:} \frac{1415\ \mathrm{cm}^{-1}\ peak\ ht}{1035\ \mathrm{cm}^{-1}\ peak\ ht}$$

Other peaks observed for this analysis were noted as they are related to the loss of organics and specific heat-induced changes [54, 55, 57]. (Table 3).

Formatted: Line spacing: single

Infrared spectroscopy can yield information regarding the relative degree of structural order, size, and strain of bioapatite crystals through the measurement of spectral peaks associated primarily with the different vibrational frequencies of bone carbonate and phosphate. This is relevant for the study of the thermal alteration of bone, as it can verify changes associated with burning that cannot be mistaken for macroscopic staining or bleaching [23]. These changes include the increase of crystallinity that accompanies burning as bone minerals become more ordered, as larger crystals grow at the expense of smaller crystals, and as the organic components are eventually lost [70, 71].

A Nicolet 6700 Fourier transform infrared spectrometer with an ATR attachment and a deuterated triglycine sulfate (DTGS) detector and single bounce diamond crystal was used. The ATR method uses an attachment with a diamond or zinc crystal to produce spectra through the phenomenon of internal reflectance [72, 73]. The application of ATR minimizes sample preparation, which in turn minimizes contamination [49, 70, 71]. Spectra were collected with 256 scans in the 4000 – 400 cm^{-1} frequency region and with an 8 mm spectral range. Each archaeological and modern bone powder sample was retested for quality control.

The identification of FTIR spectral peaks associated with bone has been extensively documented (75). Relevant peaks to this study and their inferred functional groups include the 1650 cm^{-1} and 1550 cm^{-1} peaks for the measurement of amide I and II, the 874 cm^{-1} and 1415 cm^{-1} peaks indicating presence of the v2 and v3 of carbonate, and the 900-1200 cm^{-1} and 50-600 cm^{-1} spectral regions related to the v3 and v4 phosphate components (Table 3). Additionally, the appearance of a 625cm^{-1} shoulder peak is attributed here to PO_4^{3-} v4 bending, known as the phosphate high temperature (PHT).

FTIR-ATR spectra were processed with OMNIC software to verify heat alteration and measure the relative degree of crystallinity. Thermal alteration can be monitored in bioapatite through: (1) the ratio of carbonate to phosphate present in the sample, the C/P ratio, (2) the depletion of the presence of amide I and II functional groups, representing the organic components of bone, and (3) the presence of heat specific peak splitting, such as the loss of the peak at 874 cm^{-1} correlated to CO_3^{2-} v2 at temperatures over 1000˚C, and the PHT shoulder peak at temperatures over 700˚C [57, 58, 61]. Measures of the crystallinity of a sample can be inferred from the infrared splitting factor (IRSF), which extrapolates the changing size and order of bioapatite crystals through increase of splitting seen in the PO_4^{3-} v4 peaks [57, 61, 74]. Eight peak measurements were monitored for 168 scans representing 84 individual samples for this study, 62 modern and 22 archaeological. Each sample was tested twice, and measurements presented here represent the average values of both scans.

Table 3: FTIR-ATR wavenumbers associated with likely functional groups relevant to this study and the thermal alteration of bone. Wavenumber, functional groups, and peak transformations adapted from Ellingham et al. [57], Reidsma et al. [58], and Thompson et al. [61].

Wavenumber	Inferred peak assignment	Peak transformation relevant to this study

1630-1660 cm^{-1}	organic tissue and water, amide I + II	decrease and absence
1400-1550 cm^{-1}	CO_3^{2-} v3	1415 cm^{-1} peak a component of C/P ratio
1028-1100 cm^{-1}	PO_4^{3-} v3	1035 cm^{-1} peak a component of the C/P ratio
874 cm^{-1}	CO_3^{2-} v2	peak loss
565 cm^{-1}, 605 cm^{-1}	PO_4^{3-} v4	growth of 565 cm^{-1} and 605 cm^{-1} and decrease of the 595 cm^{-1} trough utilized for the infrared splitting factor (IRSF); phosphate high temperature (PHT) shoulder growth at 625 cm^{-1}

Formatted: Line spacing: single

The IRSF measurements were procured for all samples following Weiner and Bar-Yosef [74].

$$\text{Infrared Splittng Factor:} \frac{(565\ \text{cm}^{-1}\ peak\ ht + 605\ \text{cm}^{-1}\ peak\ ht)}{595\ \text{cm}^{-1}\ peak\ ht}$$

An additional measure of the carbonate to phosphate content, the C/P ratio, was also determined for all samples. The C/P ratio decreases with burning and utilizes the 1035 cm^{-1} phosphate peak unaffected by IRSF changes [49, 71].

$$\frac{C}{P}\ \text{ratio:} \frac{1415\ \text{cm}^{-1}\ peak\ ht}{1035\ \text{cm}^{-1}\ peak\ ht}$$

Other peaks observed for this analysis were noted as they are related to the loss of organics and specific heat-induced changes [57, 58, 61] (Table 3).

X-ray diffraction analysis

X-ray Diffraction (XRD) can be used to measure the relative sizes of bioapatite crystals [32, 54, 58, 59]. [29, 42, 57, 62]. Powder XRD patterns here were obtained using Bruker D2 Phaser and Bruker D8 advance diffractometers using CuKα radiation. Bone powder samples were taken from solid bone plugs and spread with ethanol on a zero background silicon sample holder, and run from 10 to 90 °2θ with 0.02° step. Dwell time was chosen to obtain at least thousand counts on the most intense peaks. The average crystallite size of analyzed samples was obtained from diffraction peaks broadening using whole pattern fitting (Rietveld refinement) procedure as implemented in Jade MDI software [76]. Diffraction profile was modeled using hydroxyapatite $Ca_5(PO_4)_3OH$ structure (space group P63/m) and pseudo-Voigt profile shape function. The instrumental broadening was accounted for by calibration with NIST LaB_6 profile shape standard. The uncertainties in crystallite sizes are reported as obtained from least squares refinement.

Scanning Electron Microscopy

Secondary electron (SE) images were acquired for two samples of archaeological bone and three samples of modern bone annealed at different temperatures (300, 700, and 1200˚C) in a Quattro environmental scanning electron microscope (eSEM), manufactured by ThermoFischer Scientific. The SE images were obtained at an accelerating voltage of either 10kV or 20kV and with an electron beam width, or size spot, of 3.0. Spot 3 is commonly used to attain sufficient signal without compromising resolution. To prevent the buildup of charge on sample surfaces, the SE images were acquired in low vacuum mode with partial pressure of water set at 400 Pa. A low vacuum detector (LVD), which is optimized for this pressure range, was used to measure the SE image signal.

Results:

FTIR-ATR of modern bone

FTIR modern samples

Formatted: Indent: First line: 0"

The FTIR-ATR spectra were obtained for each sample, 25-1200˚C. All modern samples above 200˚C were found to exhibit spectra indicative of the thermal alteration of bone in oxygen atmospheres supported by previous research (Fig 2, 3), including the decrease of C/P ratio, decrease of organic components by 300˚C with complete absence seen by 400˚C, the absence the 874 cm^{-1} peak above 1000˚C, and the presence of the PHT peak splitting above 700˚C (S1 Table, S2 Table). No differences were indicated in the reheated or increased rate samples taken to 300 and 700˚C from the single-heated or controlled rate counterparts.

Fig. 2: FTIR-ATR spectra of experimentally modern burnt samples grouped by with functional groups highlighted in the range of 1700-800 cm^{-1}.

Fig. 3: FTIR-ATR spectra of experimentally modern burnt samples with functional groups highlighted in the range of 700-500 cm^{-1}.

The IRSF of all modern samples also followed reported trends in bioapatite crystallinity, with order, size and strain increasing alongside intensifying temperatures and clearly demonstrated with the presence of calcination (Fig. 2, 3, 4; S1 Table, S2 Table) [54, 56]. This increase in crystallinity is seen until 1000˚C, after which there is a marked decrease in IRSF coinciding with the equiaxing of bioapatite crystals (Fig. 4). Despite the acceptance of the IRSF metric and general consensus with previously described trends, the values of modern IRSF here do exhibit large variations [54, 56]. This is seen most dramatically in the range of IRSF values reported for all samples at 900˚C in this study (Fig. 4; S2 Table). No changes in crystallinity were detected in samples which were reheated or heated with increased rates.

Fig. 4: Infrared Splitting Factor (IRSF) of the experimental modern and archaeological collection measured from FTIR-ATR spectra following Weiner and Bar Yosef [81].

Formatted: Line spacing: single

The FTIR-ATR analyses were run on each modern sample for the verification of heat alteration and the determination of the size and relative order of the bioapatite crystals at each temperature threshold. All modern samples were found to exhibit spectra indicative of the thermal alteration of bone in oxygen atmospheres supported by previous research (Fig 2, 3), including the decrease of C/P ratio, decrease of organic components by 300˚C with complete absence seen by 400˚C, the absence the 874 cm^{-1} peak above 1000˚C, and the presence of the PHT peak splitting above 700˚C (SI Tables 1, 2). No differences were indicated in the reheated or increased rate samples taken to 300 and 700˚C from the single-heated or controlled rate counterparts.

The IRSF of all modern samples also followed reported trends in bioapatite crystallinity, with order, size and strain increasing alongside intensifying temperatures (Fig. 2, 3, 6; SI Tables 1, 2) [57, 60]. This increase in crystallinity is seen until 1000˚C, after which there is a marked decrease in IRSF coinciding with the equiaxing of bioapatite crystals (Fig. 6). Despite the general acceptance of the IRSF and the application of this ratio to study bone quality and thermal alteration in bone, the values of IRSF can have large variations despite consensus on general trends [57, 60]. This is seen most dramatically in the range of IRSF values reported for all samples at 900˚C in this study (Fig. 6; SI Table 1). No changes in IRSF were noted in samples reheated or heated with increased rates.

FTIR-ATR of archaeological bone

FTIR archaeological samples

The FTIR-ATR spectra produced from the T-17 archaeological collection is consistent with the Stiner et al. [27] stages of temperature intensity assignments based on color alteration. Unburnt bone is supported as non-heat altered and burnt bone does not indicate signs of intrusive staining or bleaching (Fig. 5, 6). Good agreement is seen with the relative decreases of C/P ratio and the loss of organic components by Stage 3 (fully carbonized) between the archaeological and modern samples (Fig. 5, 6; S3 Table, S4 Table). The appearance of the PHT with bones identified as Stage 5 supports the presence of temperatures above 700˚C at T-17, although the continued presence of CO_3^{2-} v2 inferred by the 874 cm^{-1} peak indicates temperatures likely did not reach above 1000˚C (Fig. 5, 6; S3 Table).

Fig. 5: FTIR-ATR spectra of archaeological fauna from T-17 grouped by stage of burning intensity following Stiner et al. [27] with functional groups highlighted in the range of 1700-800 cm^{-1}.

Fig. 6: FTIR-ATR spectra of archaeological fauna from T-17 grouped by stage of burning intensity following Stiner et al. [27] with functional groups highlighted in the range of 700-500 cm^{-1}.

The IRSF of the T-17 samples also follows the trends of the experimental modern collection, with gradual increases seen through Stage 3 (fully carbonized) and demonstrably higher values reported with the presence of calcination at Stages 4, 5, and 6 (Fig. 4, S3 Table, S4 Table). Elevated values are not seen within the Stage 0 unburnt samples of T-17 bone, demonstrating that the spontaneous recrystallization of bone mineral that accompanies diagenesis does not exceed here values of modern or archaeological samples which have been burnt at low or high temperatures. As expected, the IRSF values can distinguish between calcined and non-calcined samples but cannot distinguish between low temperature burning samples.

Formatted: Indent: First line: 0"

The FTIR-ATR spectra produced from the T-17 archaeological collection, organized by the Stiner et al. [17] stage of burning intensity, supports the macroscopic indication of heat alteration and no intrusive staining or bleaching (Fig. 4, 5). Good agreement is seen with the relative decreases of C/P ratio and the loss of organic components by Stage 3 (fully carbonized) between the archaeological and modern samples (Fig. 4, 5). The appearance of the PHT with bones identified as Stage 5 supports the presence of temperatures above 700˚C at T-17, although the continued presence of CO_3^{2-} v2 inferred by the 874 cm^{-1} peak indicates temperatures likely did not reach above 1000˚C (Fig. 4, 5).

The IRSF of the T-17 samples also follows the trends of the experimental modern collection, with gradual increases seen through Stage 3 and higher values reported with the presence of calcination with Stages 4, 5, and 6 (Fig. 6). Elevated values are not seen within the Stage 0 unburnt samples of T-17 bone, demonstrating crystallinity values were not heightened due to diagenesis [17]. As expected, the IRSF values can distinguish between calcined and non-calcined samples, but cannot distinguish between low temperature burning samples.

XRD of modern bone

The results of the XRD analyses on the modern collection demonstrate the increasing crystallite size correlated to temperature, specifically above temperatures of calcination (700˚C) (Fig. 7). An average size threshold is clearly noted, with all samples unburnt through 600˚C averaging 9 nm, while all samples burnt at 700˚C jump to an average of 41 nm (Fig. 7). An additional increase in crystallite size by approximately 30 nm is noted at 900˚C, coinciding with the fusion stage of thermal alteration of bone, with samples reaching an average of 72 nm (Fig. 7).

Formatted: Line spacing: single

XRD modern samples

Formatted: Indent: First line: 0"

The results of the XRD analyses on the modern samples demonstrate the increasing crystallite size correlated to temperature, specifically above temperatures of calcination (700˚C) (Fig. 7; S2 Table). An average size threshold is clearly noted, with all samples unburnt through 600˚C averaging 9 nm, while all samples burnt at 700˚C jump to an average of 41 nm (Fig. 7). An additional increase in crystallite size by approximately 30 nm is noted at 900˚C, coinciding with the fusion stage of thermal alteration of bone, with samples reaching an average of 72 nm (Fig. 7). The XRD results also demonstrate that within fully calcined samples, corresponding to Stiner et al. [27] burning scale Stage 6, two temperature thresholds can be distinguished: the maximum crystal size of calcined bone >700˚C not exceeding 70 nm, and the maximum crystal size of calcined bone >900-1200˚ C exceeding 90 nm (Fig. 7). This study therefore suggests average crystallite size as a metric to distinguish temperatures obtained within calcined bone classified as Stiner et al. Stage 6, > 700˚C and > 900˚C.

Fig. 7: XRD results of averaged crystallite size (nm) alongside selected SE images of experimental modern (A-C) and archaeological (D-E) bone to visualize the changes to crystal shape with heat alteration. Images A and D highlight the small, platelike shape of bioapatite burned to at least 300˚C. Images B and C, both modern, demonstrate morphological range of bones burnt above 700 ˚C, both of which are fully calcined and are considered Stage 6 on the

Stiner et al. [27] scale of burning intensity. The initial growth of hexagonal crystals ~700˚C (B) and eventual equaixing and further growth of crystals above 900 ˚C (C) are clearly seen in images B and C, and when compared to the archaeological sample of a fully calcined Stage 6 bone (E), the bioapatite crystals correspond closely to the size and shape of experimental bones burned above 900˚C.

Formatted: Line spacing: single

~~**XRD of archaeological bone**~~

XRD archaeological samples

Formatted: Indent: First line: 0"

The average crystallite size measurements for the archaeological T-17 bioapatite samples confirm that the natural postmortem recrystallization of unburnt archaeological bone does not exceed crystal sizes of modern or archaeological bones burnt to low temperatures. The crystallite sizes do increase considerably with the presence of calcination at Stiner et al. [27] Stages 4, 5, and 6 and can be distinguished from lower temperature burning and unburnt bone (Fig. 7; S4 Table). Utilizing the metric of crystal size, we also suggest that for two of the archaeological samples of Stage 6 fully calcined bone there is evidence of burning > 900˚C - <1100˚C.

Formatted: Line spacing: single

~~The average crystallite size measurements for the archaeological T-17 samples confirm the presence of calcination within samples at Stages 4, 5, and 6 with considerable crystal growth (Fig. 8). The XRD results also demonstrate that within Stage 6 temperature thresholds can be distinguished, with two of the Stage 6 samples reaching crystallite sizes that group with the ≥ 900˚C experimental group (Fig. 8). These crystal changes, including the transformation of plate-like crystals to larger equiaxed morphologies, are depicted clearly with eSEM images taken of both collections (Fig. 8).~~

~~The average crystallite growth noted in this study provides a pathway for distinguishing between temperatures within the macroscopic identification of Stage 6 burning based on color. The results presented here provide a metric to speak to two categories of burning within Stage 6: >700˚C and >900˚C.~~

~~**Discussion:**~~

Discussion

The measurements presented here regarding the loss of organics, the degree of crystallinity, and the crystallite sizes are in consensus with trends reported by previous studies, with small variance likely introduced by the burning of solid bone versus powdered samples and different experimental sample preparation and burning regimes. Our results demonstrate that for bone burnt to lower temperatures before calcination, Stiner et al. [27] Stages 1-3, bioapatite retains the small reactive crystal sizes of unburnt bone but faces rapid organic loss. The depletion of the organic component is most complete just prior to temperatures of calcination (~700˚C). This disintegration of the collagen in charred or carbonized bone is recognized to produce a very open porosity, leaving bone mineral burnt highly exposed [58].

Formatted: Highlight

The increase in crystallinity and crystal size growth seen by 700˚C corresponding to calcination and Stiner et al., [27] Stages 4-6 immediately reduces the surface to mass ratio and active surface area of bone mineral. This lower surface area results in a product with a lower solubility potential than unburnt and charred or carbonized bone. For fully calcined Stage 6

Formatted: Highlight

bones which have also been exposed to temperatures above 900˚C in oxygen atmospheres, there is the additional benefit of reported compaction and closing of the porosity, further limiting the access of any destructive agents to the exposed crystallites [58]. The heat induced dimensional changes are also considerably larger than the small amounts of diagenetic recrystallization which assists with the prevention of contamination in fresh bone, as evidenced by the inclusion of unburnt archaeological samples

Diagenetic agents such as water dissolution are therefore inferred here to be a serious threat to bone burnt to temperatures under 700˚C, because partially and wholly carbonized and charred burnt bone has low levels of crystallinity and small crystals which preserve a reactive surface area similar to unburnt fresh bone at the time of deposition. The additional lack of organic protection, open porosity, and likely small fragment sizes due to the recognized friability of burnt bones calls attention to the further vulnerability of charred and carbonized fauna in Stiner et al. [27] Stages 1-3 due to bioapatite dissolution and disintegration (Figure 8). Correspondingly, the lack of organic presence in burnt bones, especially bones corresponding to Stiner et al. [27] Stages 3-6 is interpreted here to be a less attractive target for the diagenetic agent of microbial action than fresh and fleshed unburnt bone.

Figure 8: Schematic visualization of the heat induced changes to bone porosity, organic, and inorganic components, with the shaded background area corresponding to Stages 1-3 of the Stiner et al. [27] scale of burning intensity highlighting the structural and compositional vulnerabilities of bone burnt to low temperatures to diagenetic agents, specifically water dissolution.

The results of this study propose a hypothesis of differential survivorship in bones burnt to different temperature thresholds in assemblages of archaeological fauna with fluctuating water movement. This study would further suggest that bones with low density and high porosity, such as trabecular and juvenile bone, are further susceptible to dissolution if charred or carbonized. This is due to the assumption that open porosities, likely small fragment sizes, and no organic protection exacerbates the access of diagenetic agents such as fluctuating water to their small and reactive crystals.

Bone which has been calcined, including trabecular and juvenile bone, potentially has a greater likelihood to resist dissolution due to the large crystal sizes and greater thermodynamic stability. These properties have been similarly recognized to contribute to the suitability of calcined bone for inorganic C14 dating analyses [64]. Calcined bone has been shown to be more mechanically fragile than both unburnt and charred or carbonized bone, however, and traumatic action including post-depositional movement and trampling can be highly destructive. Calcined bone therefore may be expected to preserve well in a variety of depositional environments, albeit in small fragment sizes recoverable only through screening.

Further work on the nature of burning and a full zooarchaeological profile of T-17 Unit 3 can test the hypothesis of differential survivorship, as the open-air site does have sedimentary evidence of active and fluctuating water movement. Preliminary faunal analyses indicate that there is a very low presence of bone in the Stiner et al. [27] 1-3 stages, but calcined bone identified as Stage 6 is recovered in comparatively considerable amounts (Table 2). Future work considering archaeological assemblages of burnt bone as proxies for natural and anthropogenic fire therefore should consider biases that may influence the differential survivorship of burnt bone, as properties of burnt bone vary widely between burning thresholds.

Formatted: Highlight

Conclusion

The spectroscopic measurements reported here encompass a large reference collection of burning in highly controlled conditions 100-1200˚C in fresh solid bone cores, extending previous modern and archaeological datasets that consider the structural and compositional changes to burnt bone. Especially of note is the average crystallite size difference between bone burnt above 700˚C, a metric which may be used to distinguish different burning temperatures within fully calcined archaeological bone, which are all considered as Stiner et al. [27] Stage 6.

Our results highlight the vulnerability of charred and carbonized bone to diagenetic agents and generate hypotheses regarding the differential survivorship of bone burnt to different temperatures. Here the nature of the hydrological environment is proposed to be a significant threat to bone burnt to low temperatures, as water fluctuation is a large factor in fresh bone bioapatite dissolution and charred and carbonized bone has similar bone mineral properties but has had organic protection already eliminated prior to burial.

This study reiterates the importance of small bone fragments in the study of ancient fire, observations also noted in previous studies [27,88]. Excluding the screened faunal material from T-17 would have obscured the presence of fire almost entirely, as all burnt bone except for a single fragment was recovered in the screened fraction likely due to the friability of burnt bone. The identification and recognition of further biases in addition to the mechanical fragility of burnt bone, and the variation within bones burnt to different temperatures, is emphasized here as burnt bone exhibits a range of structural and thermodynamic properties across zooarchaeological scales of visible burning intensity.

While burnt bone can provide valuable and detailed information on the properties of ancient fire relevant to behaviors of interest, the presence of fire which used fuel with low burning temperatures, such as grass, and from environments with fluctuating water interaction may not have surviving faunal evidence. Therefore, future studies should consider potential differential survivorship in archaeological assemblages of burnt bone, the impact of biased survivorship, and therefore the visibility of burnt fauna.

Formatted: Line spacing: single

~~The FTIR-ATR and XRD results in the modern and archaeological collection address timing of organic loss, changes in crystallinity, and crystallite size growth with increases in temperature intensity in oxygen atmospheres. The modern component of this study clearly demonstrates the loss of organics with carbonization and a massive crystal growth threshold that accompanies calcination by 700˚C. These modifications have large implications for the susceptibility of bone burnt to different temperatures to dissolution, especially when considered alongside the other factors inherent in bone diagenesis: organic protection and porosity.~~

~~Unburnt bone has small, highly reactive crystals but is overall less friable than burnt bone and is more likely to not be highly fragmented. Larger sizes, along with an intact insoluble organic component, protects the bone mineral from hydrolysis and microbial attack. Carbonized bone also has small, highly reactive crystals, but is more likely to be fragmented, as fragmentation is a function of burning intensity [17]. Bone burnt fully to temperatures of carbonization, 300˚C and Stiner et al. [17] Stage 3, also has the organic component eliminated resulting in the removal of protection for the bone mineral. This also results in a more open porosity which is not altered until very high temperatures, ~900˚C [61]. Carbonized and charred~~

bone, therefore, is especially vulnerable to aqueous dissolution, as it is likely to be fragmented and small, has lost organic protection, has a large degree of porosity, and has very reactive small crystals. Bone calcined by 700˚C, starting at Stiner et al. [17] Stage 4 and completely calcined by Stiner et al. [17] Stage 6, is mechanically fragile, but the massive increase in crystal sizes reduces the surface to mass ratio (active surface area) of bioapatite. This results in minerals that are much less soluble. Bone which has been thermally altered above 900˚C, which can only be identified macroscopically as Stiner et al. [17] Stage 6, benefits further from a closing of the porosity, providing additional protection.

The measurements presented here regarding the loss of organics, crystallinity, and crystallite sizes are in consensus with trends reported by previous studies, with variance likely introduced by the burning of bulk versus powder samples and the impacts of the MES phenomenon, different depositional environments under consideration, and the variance in experimental protocols and burning regimes. Additionally, treatment of modern bone is often not reported, despite the tremendous effect freezing was found to exhibit on sample porosity [6].

Due to the immediate presence of low energy water movement and the effects of prolonged exposure in the open-air environment, it is likely that the T-17 UP burnt faunal assemblage from Unit 3 is biased and has an underrepresentation of carbonized bone. This inference is in agreement with other studies of diagenesis, which have noted the large effects of water interaction on bone preservation [10, 11, 37, 44]. Despite the projected likelihood of bias in temperature distribution, the presence of fire at T-17 can be described as reaching temperatures of at least 900˚C, as evidenced by two samples having crystallite sizes grouping with the ≥ 900˚C threshold, but constrained by the continued presence of CO_3^{2-} v2 in two samples of Stage 6 FTIR-ATR spectra, which is lost at temperatures at and above 1000˚C. Future work at T-17 will investigate the microstratigraphy and association of these faunal remains to address the potential of this evidence for an in situ open air anthropogenic fire event.

Conclusion:

The aim of this study is to describe the timing of the heat induced changes to the organic and mineral components of bone, and the impact these alterations would have on burial environments with water interaction. The results are contextualized alongside an archaeological assemblage evaluated with standard zooarchaeological methods of burning identification. The importance of small bone fragments in the study of ancient fire is additionally highlighted, matching observations noted in previous studies [17, 77]. Excluding the screened faunal material, the T-17 fauna sample would have obscured the evidence for ancient fire, as nearly all burned bones except for one fragment were recovered in the < 2cm screened fraction.

Faunal remains are a valuable tool to describing fire characteristics and identifying combustion features, but bone burnt to different temperature thresholds will not be impacted uniformly by water dissolution due to the heat induced differences in crystallite size, the amount of organic components, and the degree of porosity. Studies engaging with fauna to describe ancient fires must recognize that bones burnt to lower temperatures are more vulnerable to hydrolysis and may be greatly underrepresented, while bones which are heated to the point of calcination, though mechanically fragile, have greater protection against dissolution. This bias against bone burnt to lower temperatures has large implications on the identification of ancient fire from a faunal perspective: burnt osseous material resulting from fires that utilize fuel with low burning temperatures, such as grass, and in environments with greater water interaction may be less visible in the archaeological record [18].

Acknowledgements:

The authors would like to thank Dr. Teresa Steele for her continued feedback and illuminating discussions, as well Chris Gallo for his many helpful explanations. We additionally thank Drs. Eerkens and Darwent for their generosity and lab resources, as well as the patience, assistance, and expertise of the Peter A. Rock Thermochemistry Laboratory, NEAT research group, and the Parikh Environmental Soil Chemistry Group at the University California Davis. The authors also recognize the support given from the UC Davis Veterinary Medical Teaching Hospital, the UC Davis AMCaT Laboratory, the UC Davis DHI Transdisciplinary Research Cluster "The Cluster for Archaeology and Soil Synergy", the Prehistory department of Universite Liege, and the Center for Experimental Archaeology at UC Davis (CEAD). The excavation at T-17 is supported by the NSF (PI Zwyns, Grant #156074) and the PaleoAsia Project (Izuho, Nishiaki).

References

1. Mentzer, S. M., Romano, D. G., & Voyatzis, M. E. (2017). Micromorphological contributions to the study of ritual behavior at the ash altar to Zeus on Mt. Lykaion, Greece. *Archaeological and Anthropological Sciences*, *9*(6), 1017-1043.
2. Thompson T. Recent advances in the study of burned bone and their implications for forensic anthropology. Forensic Science International. 2004;146:S203–5.
3. Ubelaker DH. The forensic evaluation of burned skeletal remains: A synthesis. Forensic Science International. 2009 Jan;183(1–3):1–5.
4. Iriarte, E., García-Tojal, J., Santana, J., Jorge-Villar, S. E., Teira, L., Muñiz, J., & Ibañez, J. J. (2020). Geochemical and spectroscopic approach to the characterization of earliest cremated human bones from the Levant (PPNB of Kharaysin, Jordan). *Journal of Archaeological Science: Reports*, *30*, 102211.
5. Costamagno S, Théry-Parisot I, Brugal J-P, Guibert R. Taphonomic consequences of the use of bones as fuel. Experimental data and archaeological applications. In: Biosphere to lithosphere: new studies in vertebrate taphonomy. Oxbow Books Oxford; 2005. p. 51–62.
6. Morin E. Taphonomic implications of the use of bone as fuel. Palethnologie. 2010;2:209-17.
7. Théry-Parisot I. Fuel management (bone and wood) during the Lower Aurignacian in the Pataud rock shelter (Lower Palaeolithic, Les Eyzies de Tayac, Dordogne, France). Contribution of experimentation. Journal of Archaeological Science. 2002;29(12):1415–21.
8. Schiegl S, Goldberg P, Pfretzschner H-U, Conard NJ. Paleolithic burnt bone horizons from the Swabian Jura: Distinguishing between in situ fireplaces and dumping areas. Geoarchaeology. 2003;18(5):541–65.
9. Costamagno S, Théry-Parisot I, Castel JC, Brugal JP. Combustible ou non? Analyse multifactorielle et modèles explicatifs sur des ossements brûlés paléolithiques. *Geston des combustibles au Paléolithique et au Mésolithique: nouveaux outils, nouvelles interpretations*. 2009:61
10. Speth J, Clark J. Hunting and overhunting in the Levantine Late Middle Palaeolithic. Before Farming. 2006 Jan;2006(3):1–42.

11. Barkai R, Rosell J, Blasco R, Gopher A. Fire for a reason: Barbecue at middle Pleistocene Qesem cave, Israel. Current Anthropology. 2017 Aug 1;58(S16):S314-28.
12. Turner-Walker G. The Chemical and Microbial Degradation of Bones and Teeth. In: Pinhasi R, Mays S, editors. Advances in Human Palaeopathology [Internet]. Chichester, UK: John Wiley & Sons, Ltd; 2007:3–29.
13. Binford LR, Bertram JB. Bone frequencies and attritional processes. In (LR Binford, Ed.) For Theory Building in Archaeology. 1977.
14. Lyman RL. Bone density and differential survivorship of fossil classes. Journal of Anthropological archaeology. 1984;3(4):259–99.
15. Grayson DK. Bone transport, bone destruction, and reverse utility curves. Journal of Archaeological Science. 1989 Nov 1;16(6):643–52.
16. Outram AK. A new approach to identifying bone marrow and grease exploitation: why the “indeterminate” fragments should not be ignored. Journal of archaeological science. 2001;28(4):401–10.

17. Munson PJ, Garniewicz RC. Age-mediated survivorship of ungulate mandibles and teeth in canid-ravaged faunal assemblages. Journal of Archaeological Science. 2003;30(4):405–16.
18. Robinson S, Nicholson RA, Pollard AM, O'Connor TP. An Evaluation of Nitrogen Porosimetry as a Technique for Predicting Taphonomic Durability in Animal Bone. Journal of Archaeological Science. 2003 Apr;30(4):391–403.
19. Von Endt DW, Ortner DJ. Experimental effects of bone size and temperature on bone diagenesis. Journal of Archaeological Science. 1984 May;11(3):247–53.
20. Lam YM, Chen X, Pearson OM. Intertaxonomic variability in patterns of bone density and the differential representation of bovid, cervid, and equid elements in the archaeological record. American Antiquity. 1999;64(2):343–62.
21. Lyman RL. Vertebrate taphonomy. Cambridge University Press; 1994.
22. Nielsen-Marsh CM, Hedges REM. Patterns of Diagenesis in Bone I: The Effects of Site Environments. Journal of Archaeological Science. 2000 Dec;27(12):1139–50.
23. Nielsen-Marsh CM, Smith CI, Jans MME, Nord A, Kars H, Collins MJ. Bone diagenesis in the European Holocene II: taphonomic and environmental considerations. Journal of Archaeological Science. 2007 Sep;34(9):1523–31.
24. Nielsen-Marsh C, Gernaey A, Turner-Walker G, Hedges R, Pike A, Collins M. The chemical degradation of bone. Human Osteology in archaeology and forensic science. 2000: 439-454.
25. Denys C. Taphonomy and experimentation. Archaeometry. 2002 Aug;44(3):469–84.
26. Maurer A-F, Person A, Tütken T, Amblard-Pison S, Ségalen L. Bone diagenesis in arid environments: An intra-skeletal approach. Palaeogeography, Palaeoclimatology, Palaeoecology. 2014 Dec;416:17–29.
27. Stiner MC, Kuhn SL, Weiner S, Bar-Yosef O. Differential Burning, Recrystallization, and Fragmentation of Archaeological Bone. Journal of Archaeological Science. 1995 Mar;22(2):223–37.
28. Martin RB, Burr DB, Sharkey NA, Fyhrie DP. Skeletal tissue mechanics. Second edition. New York: Springer; 2015. 762 p.
29. Stout SD, Cole ME, Agnew AM. Histomorphology. In: Ortner's Identification of Pathological Conditions in Human Skeletal Remains [Internet]. Elsevier; 2019 [cited 2019 Oct 20]. p. 91–167.
30. Rollin-Martinet S, Navrotsky A, Champion E, Grossin D, Drouet C. Thermodynamic basis for evolution of apatite in calcified tissues. American Mineralogist. 2013 Nov 1;98(11–12):2037–45.
31. Drouet C, Aufray M, Rollin-Martinet S, Vandecandelaère N, Grossin D, Rossignol F, et al. Nanocrystalline apatites: The fundamental role of water. American Mineralogist. 2018 Apr 1;103(4):550–64.
32. Figueiredo M, Fernando A, Martins G, Freitas J, Judas F, Figueiredo H. Effect of the calcination temperature on the composition and microstructure of hydroxyapatite derived from human and animal bone. Ceramics International. 2010 Dec;36(8):2383–93
33. Greiner M, Rodríguez-Navarro A, Heinig MF, Mayer K, Kocsis B, Göhring A, et al. Bone incineration: An experimental study on mineral structure, colour and crystalline state. Journal of Archaeological Science: Reports. 2019;25:507–18.
34. Rey C, Combes C, Drouet C, Glimcher MJ. Bone mineral: update on chemical composition and structure. Osteoporos Int. 2009 Jun;20(6):1013–21.

35. Bala Y, Farlay D, Boivin G. Bone mineralization: from tissue to crystal in normal and pathological contexts. Osteoporos Int. 2013 Aug;24(8):2153–66.
36. Berna F, Matthews A, Weiner S. Solubilities of bone mineral from archaeological sites: the recrystallization window. Journal of archaeological Science. 2004 Jul 1;31(7):867-82.
37. Nyman JS, Ni Q, Nicolella DP, Wang X. Measurements of mobile and bound water by nuclear magnetic resonance correlate with mechanical properties of bone. Bone. 2008;42(1):193–9.
38. Trueman CN, Privat K, Field J. Why do crystallinity values fail to predict the extent of diagenetic alteration of bone mineral? Palaeogeography, Palaeoclimatology, Palaeoecology. 2008 Sep;266(3–4):160–7.
39. Koeppenkastrop D, Eric H. Sorption of rare-earth elements from seawater onto synthetic mineral particles: An experimental approach. Chemical geology. 1992;95(3–4):251–63.
40. Reynard B, Lécuyer C, Grandjean P. Crystal-chemical controls on rare-earth element concentrations in fossil biogenic apatites and implications for paleoenvironmental reconstructions. Chemical Geology. 1999;155(3–4):233–41.
41. Buikstra JE, Swegle M. Bone modification due to burning: experimental evidence. Bone modification. 1989;247–58.
42. Tripp JA, Squire ME, Hedges REM, Stevens RE. Use of micro-computed tomography imaging and porosity measurements as indicators of collagen preservation in archaeological bone. Palaeogeography, Palaeoclimatology, Palaeoecology. 2018 Dec;511:462–71.
43. Smith CI, Nielsen-Marsh CM, Jans MME, Collins MJ. Bone diagenesis in the European Holocene I: patterns and mechanisms. Journal of Archaeological Science. 2007;34(9):1485–93.
44. Hedges REM, Millard AR. Bones and Groundwater: Towards the Modelling of Diagenetic Processes. Journal of Archaeological Science. 1995 Mar;22(2):155–64.
45. Hedges REM. Bone diagenesis: an overview of processes. Archaeometry. 2002 Aug;44(3):319–28.
46. Wescott, D. J. (2019). Postmortem change in bone biomechanical properties: Loss of plasticity. *Forensic science international*, *300*, 164-169.
47. Trueman CN, Behrensmeyer AK, Potts R, Tuross N. High-resolution records of location and stratigraphic provenance from the rare earth element composition of fossil bones. Geochimica et Cosmochimica Acta. 2006 Sep;70(17):4343–55.
48. Baig AA. Influences of carbonate content and crystallinity on the solubility behavior of synthetic and biological apatites. Department of Pharmaceutics and Pharmaceutical Chemistry, University of Utah; 1997.
49. Baig AA, Fox JL, Wang Z, Higuchi WI, Miller SC, Barry AM, et al. Metastable Equilibrium Solubility Behavior of Bone Mineral. Calcified Tissue International. 1999 Apr 1;64(4):329–39.
50. Trueman, C. N., & Tuross, N. (2002). Trace elements in recent and fossil bone apatite. *Reviews in mineralogy and geochemistry*, *48*(1), 489-521.
51. Pfretzschner H-U. Fossilization of Haversian bone in aquatic environments. Comptes Rendus Palevol. 2004 Oct;3(6–7):605–16.
52. Shipman P, Foster G, Schoeninger M. Burnt bones and teeth: an experimental study of color, morphology, crystal structure and shrinkage. Journal of Archaeological Science. 1984 Jul;11(4):307–25.

53. Thompson T, Ulgium, P. Burned Human Remains. In Handbook of Forensic Anthropology and Archaeology. Routledge. 2016: 295-303.
54. Ellingham STD, Thompson TJU, Islam M, Taylor G. Estimating temperature exposure of burnt bone — A methodological review. Science & Justice. 2015 May;55(3):181–8.
55. Reidsma FH, van Hoesel A, van Os BJH, Megens L, Braadbaart F. Charred bone: Physical and chemical changes during laboratory simulated heating under reducing conditions and its relevance for the study of fire use in archaeology. Journal of Archaeological Science: Reports. 2016 Dec;10:282–92.
56. Thompson TJU. The Analysis of Heat-Induced Crystallinity Change in Bone. In: The Analysis of Burned Human Remains. Elsevier; 2015. p. 323–37.
57. Thompson TJU, Islam M, Bonniere M. A new statistical approach for determining the crystallinity of heat-altered bone mineral from FTIR spectra. Journal of Archaeological Science. 2013 Jan;40(1):416–22.
58. Etok SE, Valsami-Jones E, Wess TJ, Hiller JC, Maxwell CA, Rogers KD, et al. Structural and chemical changes of thermally treated bone apatite. J Mater Sci. 2007 Sep 21;42(23):9807–16.
59. Pramanik S, Hanif A, Pingguan-Murphy B, Abu Osman N. Morphological Change of Heat Treated Bovine Bone: A Comparative Study. Materials. 2013. Dec 21;6(1):65–75.
60. Waterhouse, K. (2013). The effect of weather conditions on burnt bone fragmentation. *Journal of forensic and legal medicine*, *20*(5), 489-495.
61. Herrmann, N. P., & Bennett, J. L. (1999). The differentiation of traumatic and heat-related fractures in burned bone. *Journal of Forensic Science*, *44*(3), 461-469.
62. Wang, X., Bank, R. A., TeKoppele, J. M., Hubbard, G. B., Althanasiou, K. A., & Agrawal, C. M. (2000). Effect of collagen denaturation on the toughness of bone. *Clinical Orthopaedics and Related Research®*, *371*, 228-239.
63. McKinley, J. I. (1994). Bone fragment size in British cremation burials and its implications for pyre technology and ritual. *Journal of Archaeological Science*, *21*(3), 339-342.
64. Lanting, J. N., Aerts-Bijma, A. T., & van der Plicht, J. (2001). Dating of cremated bones. *Radiocarbon*, *43*(2A), 249-254.
65. Major, I., Dani, J., Kiss, V., Melis, E., Patay, R., Szabó, G., ... & Jull, T. A. (2018). Adoption and evaluation of a sample pretreatment protocol for radiocarbon dating of cremated bones at HEKAL. *Radiocarbon*.
66. Zazzo, A., & Saliège, J. F. (2011). Radiocarbon dating of biological apatites: a review. *Palaeogeography, Palaeoclimatology, Palaeoecology*, *310*(1-2), 52-61.

67. Zwyns N, Paine CH, Tsedendorj B, Talamo S, Fitzsimmons KE, Gantumur A, et al. The Northern Route for Human dispersal in Central and Northeast Asia: New evidence from the site of Tolbor-16, Mongolia. Sci Rep. 2019 Dec;9(1):11759.
68. Zwyns N, Gladyshev SA, Gunchinsuren B, Bolorbat T, Flas D, Dogandžić T, et al. The open-air site of Tolbor 16 (Northern Mongolia): Preliminary results and perspectives. Quaternary International. 2014 Oct;347:53–65.
69. Gladyshev SA, Olsen JW, Tabarev AV, Jull AJT. The Upper Paleolithic of Mongolia: Recent finds and new perspectives. Quaternary International. 2012 Dec;281:36–46.
70. Tabarev A., Gunchinsuren .B, Gillam J., Gladyshev S., Dogandžić T., Zwyns N. Kompleks Pamiatnikov Kamennogo Veka v Doline r.Ikh Tulberiin-Gol, Severnaya Mongolia (razvedochnye raboty s ispolzovaniem GIS-Technnologii v. 2011 g) [The

Stone Age site complex in the Ikh-Tulberiin-Gol River Valley, North Mongolia (survey and research using GIS-technology in 2011)]. Studia Archaeologica Instituti Archaeologici Academiae Scientiarum Mongolicae. 2012;32:26–43

71. Gowlett JAJ. The early settlement of northern Europe: Fire history in the context of climate change and the social brain. Comptes Rendus Palevol. 2006 Jan;5(1–2):299–310.
72. Rybin EP, Khatsenovich AM, Gunchinsuren B, Olsen JW, Zwyns N. The impact of the LGM on the development of the Upper Paleolithic in Mongolia. Quaternary International. 2016 Dec;425:69–87.
73. Beasley MM, Bartelink EJ, Taylor L, Miller RM. Comparison of transmission FTIR, ATR, and DRIFT spectra: implications for assessment of bone bioapatite diagenesis. Journal of Archaeological Science. 2014 Jun;46:16–22.
74. Lebon, M., Zazzo, A., & Reiche, I. (2014). Screening in situ bone and teeth preservation by ATR-FTIR mapping. *Palaeogeography, Palaeoclimatology, Palaeoecology*, *416*, 110-119.
75. Lebon, M., Reiche, I., Gallet, X., Bellot-Gurlet, L., & Zazzo, A. (2016). Rapid quantification of bone collagen content by ATR-FTIR spectroscopy. *Radiocarbon*, *58*(1), 131.
76. Trueman, C. N., Behrensmeyer, A. K., Tuross, N., & Weiner, S. (2004). Mineralogical and compositional changes in bones exposed on soil surfaces in Amboseli National Park, Kenya: diagenetic mechanisms and the role of sediment pore fluids. *Journal of Archaeological Science*, *31*(6), 721-739.
77. Dal Sasso, G., Asscher, Y., Angelini, I., Nodari, L., & Artioli, G. (2018). A universal curve of apatite crystallinity for the assessment of bone integrity and preservation. *Scientific reports*, *8*(1), 1-13.
78. Gianfrate, G., D'Elia, M., Quarta, G., Giotta, L., Valli, L., & Calcagnile, L. (2007). Qualitative application based on IR spectroscopy for bone sample quality control in radiocarbon dating. *Nuclear Instruments and Methods in Physics Research Section B: Beam Interactions with Materials and Atoms*, *259*(1), 316-319.
79. Lebon, M., Reiche, I., Fröhlich, F., Bahain, J. J., & Falguères, C. (2008). Characterization of archaeological burnt bones: contribution of a new analytical protocol based on derivative FTIR spectroscopy and curve fitting of the ν 1 ν 3 PO 4 domain. *Analytical and Bioanalytical Chemistry*, *392*(7-8), 1479-1488.
80. Snoeck, C., Lee-Thorp, J. A., & Schulting, R. J. (2014). From bone to ash: Compositional and structural changes in burned modern and archaeological bone. *Palaeogeography, palaeoclimatology, palaeoecology*, *416*, 55-68.

81. Weiner S, Bar-Yosef O. States of preservation of bones from prehistoric sites in the Near East: A survey. Journal of Archaeological Science. 1990 Mar;17(2):187–96.
82. Thompson TJU, Gauthier M, Islam M. The application of a new method of Fourier Transform Infrared Spectroscopy to the analysis of burned bone. Journal of Archaeological Science. 2009 Mar;36(3):910–4.
83. Paschalis EP, DiCarlo E, Betts F, Sherman P, Mendelsohn R, Boskey AL. FTIR microspectroscopic analysis of human osteonal bone. Calcified tissue international. 1996 Dec 1;59(6):480-7.
84. Hollund HI, Ariese F, Fernandes R, Jans MME, Kars H. Testing an alternative high-throughput tool for investigating bone diagenesis: FTIR in attenuated total reflection

(ATR) mode: An alternative tool for investigating bone diagenesis. Archaeometry. 2013 Jun;55(3):507–32.

85. Nakamoto K. Infrared and Raman Spectra of Inorganic and Coordination Compounds (Part A: Theory and Applications in Inorganic Chemistry)(Volume 1A)(Part B: Applications in Coordination, Organometallic, and Bioinorganic Chemistry)(Volume 1B). NY, John Wiley & Sons, Incorporated; 1997

86. Bruno TJ. Sampling Accessories for Infrared Spectrometry. Applied Spectroscopy Reviews. 1999 Jul 20;34(1–2):91–120.

87. Rietveld H. A profile refinement method for nuclear and magnetic structures. Journal of applied Crystallography. 1969 Jun 2;2(2):65-71.

88. Villa P, Castel JC, Beauval C, Bourdillat V, Goldberg P. Human and carnivore sites in the European Middle and Upper Paleolithic: similarities and differences in bone modification and fragmentation. Revue de paléobiologie. 2004;23(2):705-30.

References:

1. Binford LR, Bertram JB. Bone frequencies and attritional processes. In (LR Binford, Ed.) For Theory Building in Archaeology. 1977
2. Lyman RL. Bone density and differential survivorship of fossil classes. Journal of Anthropological archaeology. 1984;3(4):259–99.
3. Grayson DK. Bone transport, bone destruction, and reverse utility curves. Journal of Archaeological Science. 1989 Nov 1;16(6):643–52.
4. Outram AK. A new approach to identifying bone marrow and grease exploitation: why the "indeterminate" fragments should not be ignored. Journal of archaeological science. 2001;28(4):401–10.
5. Munson PJ, Garniewicz RC. Age-mediated survivorship of ungulate mandibles and teeth in canid-ravaged faunal assemblages. Journal of Archaeological Science. 2003;30(4):405–16
6. Robinson S, Nicholson RA, Pollard AM, O'Connor TP. An Evaluation of Nitrogen Porosimetry as a Technique for Predicting Taphonomic Durability in Animal Bone. Journal of Archaeological Science. 2003 Apr;30(4):391–403.
7. Von Endt DW, Ortner DJ. Experimental effects of bone size and temperature on bone diagenesis. Journal of Archaeological Science. 1984 May;11(3):247–53.
8. Lam YM, Chen X, Pearson OM. Intertaxonomic variability in patterns of bone density and the differential representation of bovid, cervid, and equid elements in the archaeological record. American Antiquity. 1999;64(2):343–62.
9. Lyman RL. Vertebrate taphonomy. Cambridge University Press; 1994.
10. Nielsen-Marsh CM, Hedges REM. Patterns of Diagenesis in Bone I: The Effects of Site Environments. Journal of Archaeological Science. 2000 Dec;27(12):1139–50.
11. Nielsen-Marsh CM, Smith CI, Jans MME, Nord A, Kars H, Collins MJ. Bone diagenesis in the European Holocene II: taphonomic and environmental considerations. Journal of Archaeological Science. 2007 Sep;34(9):1523–31.
12. Nielsen-Marsh C, Gernaey A, Turner-Walker G, Hedges R, Pike A, Collins M. The chemical degradation of bone. Human Osteology in archaeology and forensic science. 2000: 439-454.
13. Denys C. Taphonomy and experimentation. Archaeometry. 2002 Aug;44(3):469–84.
14. Maurer A-F, Person A, Tütken T, Amblard-Pison S, Ségalen L. Bone diagenesis in arid environments: An intra-skeletal approach. Palaeogeography, Palaeoclimatology, Palaeoecology. 2014 Dec;416:17–29.
15. Turner-Walker G. The Chemical and Microbial Degradation of Bones and Teeth. In: Pinhasi R, Mays S, editors. Advances in Human Palaeopathology [Internet]. Chichester, UK: John Wiley & Sons, Ltd; 2007:3–29.
16. Costamagno S, Théry-Parisot I, Brugal J-P, Guibert R. Taphonomic consequences of the use of bones as fuel. Experimental data and archaeological applications. In: Biosphere to lithosphere: new studies in vertebrate taphonomy. Oxbow Books Oxford; 2005. p. 51–62.
17. Stiner MC, Kuhn SL, Weiner S, Bar-Yosef O. Differential Burning, Recrystallization, and Fragmentation of Archaeological Bone. Journal of Archaeological Science. 1995 Mar;22(2):223–37.
18. Berna F, Goldberg P, Horwitz LK, Brink J, Holt S, Bamford M, et al. Microstratigraphic evidence of in situ fire in the Acheulean strata of Wonderwerk Cave, Northern Cape province, South Africa. Proceedings of the National Academy of Sciences. 2012 May 15;109(20):E1215–20.
19. Goldberg P, Dibble H, Berna F, Sandgathe D, McPherron SJP, Turq A. New evidence on Neandertal use of fire: Examples from Roc de Marsal and Pech de l'Azé IV. Quaternary International. 2012 Jan;247:325–40.

20. Barkai R, Rosell J, Blasco R, Gopher A. Fire for a reason: Barbecue at middle Pleistocene Qesem cave, Israel. Current Anthropology. 2017 Aug 1;58(S16):S314-28.
21. Thompson T. Recent advances in the study of burned bone and their implications for forensic anthropology. Forensic Science International. 2004;146:S203–5.
22. Ubelaker DH. The forensic evaluation of burned skeletal remains: A synthesis. Forensic Science International. 2009 Jan;183(1–3):1–5.
23. Morin E. Taphonomic implications of the use of bone as fuel. Palethnologie. 2010;2:209-17.
24. Théry-Parisot I. Fuel management (bone and wood) during the Lower Aurignacian in the Pataud rock shelter (Lower Palaeolithic, Les Eyzies de Tayac, Dordogne, France). Contribution of experimentation. Journal of Archaeological Science. 2002;29(12):1415–21.
25. Schiegl S, Goldberg P, Pfretzschner H-U, Conard NJ. Paleolithic burnt bone horizons from the Swabian Jura: Distinguishing between in situ fireplaces and dumping areas. Geoarchaeology. 2003;18(5):541–65.
26. Speth J, Clark J. Hunting and overhunting in the Levantine Late Middle Palaeolithic. Before Farming. 2006 Jan;2006(3):1–42.
27. Costamagno S, Théry-Parisot I, Castel JC, Brugal JP. Combustible ou non? Analyse multifactorielle et modèles explicatifs sur des ossements brûlés paléolithiques. *Geston des combustibles au Paléolithique et au Mésolithique: nouveaux outils, nouvelles interpretations*. 2009:61
28. Buikstra JE, Swegle M. Bone modification due to burning: experimental evidence. Bone modification. 1989;247–58.
29. Etok SE, Valsami-Jones E, Wess TJ, Hiller JC, Maxwell CA, Rogers KD, et al. Structural and chemical changes of thermally treated bone apatite. J Mater Sci. 2007 Sep 21;42(23):9807–16.
30. Martin RB, Burr DB, Sharkey NA, Fyhrie DP. Skeletal tissue mechanics. Second edition. New York: Springer; 2015. 762 p.
31. Rollin-Martinet S, Navrotsky A, Champion E, Grossin D, Drouet C. Thermodynamic basis for evolution of apatite in calcified tissues. American Mineralogist. 2013 Nov 1;98(11–12):2037–45.
32. Drouet C, Aufray M, Rollin-Martinet S, Vandecandelaère N, Grossin D, Rossignol F, et al. Nanocrystalline apatites: The fundamental role of water. American Mineralogist. 2018 Apr 1;103(4):550–64.
33. Stout SD, Cole ME, Agnew AM. Histomorphology. In: Ortner's Identification of Pathological Conditions in Human Skeletal Remains [Internet]. Elsevier; 2019 [cited 2019 Oct 20]. p. 91–167.
34. Rey C, Combes C, Drouet C, Glimcher MJ. Bone mineral: update on chemical composition and structure. Osteoporos Int. 2009 Jun;20(6):1013–21.
35. Greiner M, Rodríguez-Navarro A, Heinig MF, Mayer K, Kocsis B, Göhring A, et al. Bone incineration: An experimental study on mineral structure, colour and crystalline state. Journal of Archaeological Science: Reports. 2019;25:507–18.
36. Bala Y, Farlay D, Boivin G. Bone mineralization: from tissue to crystal in normal and pathological contexts. Osteoporos Int. 2013 Aug;24(8):2153–66..
37. Berna F, Matthews A, Weiner S. Solubilities of bone mineral from archaeological sites: the recrystallization window. Journal of archaeological Science. 2004 Jul 1;31(7):867-82.
38. Nyman JS, Ni Q, Nicolella DP, Wang X. Measurements of mobile and bound water by nuclear magnetic resonance correlate with mechanical properties of bone. Bone. 2008;42(1):193–9.
39. Trueman CN, Privat K, Field J. Why do crystallinity values fail to predict the extent of diagenetic alteration of bone mineral? Palaeogeography, Palaeoclimatology, Palaeoecology. 2008 Sep;266(3–4):160–7.
40. Koeppenkastrop D, Eric H. Sorption of rare-earth elements from seawater onto synthetic mineral particles: An experimental approach. Chemical geology. 1992;95(3–4):251–63.
41. Reynard B, Lécuyer C, Grandjean P. Crystal-chemical controls on rare-earth element concentrations in fossil biogenic apatites and implications for paleoenvironmental reconstructions. Chemical Geology. 1999;155(3–4):233–41.
42. Figueiredo M, Fernando A, Martins G, Freitas J, Judas F, Figueiredo H. Effect of the calcination temperature on the composition and microstructure of hydroxyapatite derived from human and animal bone. Ceramics International. 2010 Dec;36(8):2383–93.
43. Tripp JA, Squire ME, Hedges REM, Stevens RE. Use of micro-computed tomography imaging and porosity measurements as indicators of collagen preservation in archaeological bone. Palaeogeography, Palaeoclimatology, Palaeoecology. 2018 Dec;511:462–71.

44. Smith CI, Nielsen-Marsh CM, Jans MME, Collins MJ. Bone diagenesis in the European Holocene I: patterns and mechanisms. Journal of Archaeological Science. 2007;34(9):1485–93.
45. Hedges REM, Millard AR. Bones and Groundwater: Towards the Modelling of Diagenetic Processes. Journal of Archaeological Science. 1995 Mar;22(2):155–64.
46. Hedges REM. Bone diagenesis: an overview of processes. Archaeometry. 2002 Aug;44(3):319–28.
47. Pfretzschner H-U. Fossilization of Haversian bone in aquatic environments. Comptes Rendus Palevol. 2004 Oct;3(6–7):605–16.
48. Tütken T, Vennemann TW, Pfretzschner H-U. Early diagenesis of bone and tooth apatite in fluvial and marine settings: Constraints from combined oxygen isotope, nitrogen and REE analysis. Palaeogeography, Palaeoclimatology, Palaeoecology. 2008 Sep;266(3–4):254–68.
49. Beasley MM, Bartelink EJ, Taylor L, Miller RM. Comparison of transmission FTIR, ATR, and DRIFT spectra: implications for assessment of bone bioapatite diagenesis. Journal of Archaeological Science. 2014 Jun;46:16–22.
50. Trueman CN, Behrensmeyer AK, Potts R, Tuross N. High-resolution records of location and stratigraphic provenance from the rare earth element composition of fossil bones. Geochimica et Cosmochimica Acta. 2006 Sep;70(17):4343–55.
51. Baig AA. Influences of carbonate content and crystallinity on the solubility behavior of synthetic and biological apatites. Department of Pharmaceutics and Pharmaceutical Chemistry, University of Utah; 1997.
52. Baig AA, Fox JL, Wang Z, Higuchi WI, Miller SC, Barry AM, et al. Metastable Equilibrium Solubility Behavior of Bone Mineral. Calcified Tissue International. 1999 Apr 1;64(4):329–39.
53. LeGeros RZ, Bonel G, Legros R. Types of "H 2 O" in human enamel and in precipitated apatites, Calcified Tissue Research. 1978 Dec 1; 26(1):111-8.
54. White EM, Hannus LA. Chemical Weathering of Bone in Archaeological Soils. American Antiquity. 1983;48(2):316–22.
55. Shipman P, Foster G, Schoeninger M. Burnt bones and teeth: an experimental study of color, morphology, crystal structure and shrinkage. Journal of Archaeological Science. 1984 Jul;11(4):307–25.
56. Thompson T, Ulgium, P. Burned Human Remains. In Handbook of Forensic Anthropology and Archaeology. Routledge. 2016: 295-303.
57. Ellingham STD, Thompson TJU, Islam M, Taylor G. Estimating temperature exposure of burnt bone — A methodological review. Science & Justice. 2015 May;55(3):181–8.
58. Reidsma FH, van Hoesel A, van Os BJH, Megens L, Braadbaart F. Charred bone: Physical and chemical changes during laboratory simulated heating under reducing conditions and its relevance for the study of fire use in archaeology. Journal of Archaeological Science: Reports. 2016 Dec;10:282–92.
59. Shahack-Gross R, Bar-Yosef O, Weiner S. Black-coloured bones in Hayonim Cave, Israel: differentiating between burning and oxide staining. Journal of archaeological Science. 1997 May 1;24(5):439-46.
60. Thompson TJU. The Analysis of Heat-Induced Crystallinity Change in Bone. In: The Analysis of Burned Human Remains. Elsevier; 2015. p. 323–37.
61. Thompson TJU, Islam M, Bonniere M. A new statistical approach for determining the crystallinity of heat-altered bone mineral from FTIR spectra. Journal of Archaeological Science. 2013 Jan;40(1):416–22.
62. Pramanik S, Hanif A, Pingguan-Murphy B, Abu Osman N. Morphological Change of Heat Treated Bovine Bone: A Comparative Study. Materials. 2012 Dec 21;6(1):65–75.
63. Ubelaker DH, Rife JL. The practice of cremation in the Roman-era cemetery at Kenchreai, Greece. Bioarchaeology of the Near East. 2007;1:35-57.
64. Zwyns N, Paine CH, Tsedendorj B, Talamo S, Fitzsimmons KE, Gantumur A, et al. The Northern Route for Human dispersal in Central and Northeast Asia: New evidence from the site of Tolbor-16, Mongolia. Sci Rep. 2019 Dec;9(1):11759.
65. Zwyns N, Gladyshev SA, Gunchinsuren B, Bolorbat T, Flas D, Dogandžić T, et al. The open-air site of Tolbor 16 (Northern Mongolia): Preliminary results and perspectives. Quaternary International. 2014 Oct;347:53–65.
66. Gladyshev SA, Olsen JW, Tabarev AV, Jull AJT. The Upper Paleolithic of Mongolia: Recent finds and new perspectives. Quaternary International. 2012 Dec;281:36–46.
67. Tabarev A., Gunchinsuren .B, Gillam J., Gladyshev S., Dogandžić T., Zwyns N. Kompleks Pamiatnikov Kamennogo Veka v Doline r.Ikh Tulberiin-Gol, Severnaya Mongolia (razvedochnye raboty s ispolzovaniem GIS-Technnologii v. 2011 g) [The Stone Age site complex in the Ikh-Tulberiin-Gol River

Valley, North Mongolia (survey and research using GIS-technology in 2011)]. Studia Archaeologica Instituti Archaeologici Academiae Scientiarum Mongolicae. 2012;32:26–43
68. Gowlett JAJ. The early settlement of northern Europe: Fire history in the context of climate change and the social brain. Comptes Rendus Palevol. 2006 Jan;5(1–2):299–310.
69. Rybin EP, Khatsenovich AM, Gunchinsuren B, Olsen JW, Zwyns N. The impact of the LGM on the development of the Upper Paleolithic in Mongolia. Quaternary International. 2016 Dec;425:69–87.
70. Hollund HI, Ariese F, Fernandes R, Jans MME, Kars H. Testing an alternative high-throughput tool for investigating bone diagenesis: FTIR in attenuated total reflection (ATR) mode: An alternative tool for investigating bone diagenesis. Archaeometry. 2013 Jun;55(3):507–32.
71. Thompson TJU, Gauthier M, Islam M. The application of a new method of Fourier Transform Infrared Spectroscopy to the analysis of burned bone. Journal of Archaeological Science. 2009 Mar;36(3):910–4.
72. Nakamoto K. Infrared and Raman Spectra of Inorganic and Coordination Compounds (Part A: Theory and Applications in Inorganic Chemistry)(Volume 1A)(Part B: Applications in Coordination, Organometallic, and Bioinorganic Chemistry)(Volume 1B). NY, John Wiley & Sons, Incorporated; 1997.
73. Bruno TJ. Sampling Accessories for Infrared Spectrometry. Applied Spectroscopy Reviews. 1999 Jul 20;34(1–2):91–120.
74. Weiner S, Bar-Yosef O. States of preservation of bones from prehistoric sites in the Near East: A survey. Journal of Archaeological Science. 1990 Mar;17(2):187–96.

75. Paschalis EP, DiCarlo E, Betts F, Sherman P, Mendelsohn R, Boskey AL. FTIR microspectroscopic analysis of human osteonal bone. Calcified tissue international. 1996 Dec 1;59(6):480-7.

76. Rietveld H. A profile refinement method for nuclear and magnetic structures. Journal of applied Crystallography. 1969 Jun 2;2(2):65-71.
77. Villa P, Castel JC, Beauval C, Bourdillat V, Goldberg P. Human and carnivore sites in the European Middle and Upper Paleolithic: similarities and differences in bone modification and fragmentation. Revue de paléobiologie. 2004;23(2):705-30.

Response to Reviewers:

Reviewer 1

…However, the originality of the paper needs to be more explicitly described, crystallinity changes are referred to burning but very little if any is referred to diagenetic influence in burnt archaeological specimens (which is the objective of the paper but finally not clearly discussed). A reorganization of the text and more clear statements and aspects to be discussed to demonstrate the goals of the paper is needed.

Thank you for your comment and for all your helpful suggestions. The manuscript is greatly improved with your feedback. To highlight the originality of the paper we have rewritten major portions of the introduction and background sections, attempting to summarize concisely the exact goals of the study and inclusion of the modern and archaeological samples. We believe this will reframe and clarify the role of the Tolbor-17 material for this study, as it is our intention that the Tobor-17 archaeological burnt bone serve as a comparative reference sample of changes in bioapatite crystallinity sizes and organic content for this study. Future zooarchaeological studies planned for Tolbor-17 will consider the hypothesis of differential preservation closely with supporting faunal and geoarchaeological data. We have therefore modified our conclusions to reflect this restructuring, using our results from the study as hypothesis generating observations for further closer study, with the intention to follow up more substantially in the forthcoming full zooarchaeological study of Tolbor-17.

Burning alone does not cause fragmentation, a subsequent movement or effort may produce fragmentation, but indication of which movement or effort acted after burning is not described. Further, fragmentation before and after burning can be caused by a large number of taphonomic agents (e.g. butchery, trampling, weathering, corrosion…).

Thank you for your observation. This perspective and related information will be the major focus of the next study planned for this project: a full zooarchaeological study of the Tolbor-17 faunal material. This planned study will include traditional zooarchaeological analyses including identifiable specimens, fragmentation degree, surface modifications, and preservation. We seek to describe many anthropogenic behaviors present in the Tolbor-17 fauna in the future study, and will pay specific attention to burning to test the hypothesis of differential preservation suggested by our current manuscript. This zooarchaeological investigation has had initial data collection completed in December 2019, with further analyses planned with international travel restrictions are lifted.

Definitively, the paper needs a better and more exhaustive description of the fossil assemblage: number of specimens, size of fossils, surface modifications, how many fossils are in each burning stage or which have also been affected by any other taphonomic agent both before and after burning should be described or included in a table.

We believe that through the reframing and clarification of the purpose of the archaeological material (as a reference sample confirming the visibility in changes of bioapatite crystallinity sizes and organic preservation in an archaeological assemblage and relating described changes to zooarchaeological observational scales of burning intensity) will clarify the intention of the archaeological sample inclusion. A sample of text addressing this specifically is now included in the introduction and is highlighted in the manuscript with tracked changes on page 3. We also have reframed our discussion of the archaeological material in the context of hypothesis generation, and the descriptive fossil assemblage desired here will be the focus of the next paper engaging with the planned zooarchaeological study of the full Tolbor-17 fauna from Unit 3. Language addressing this has been rewritten and is highlighted in page 23 of the manuscript with tracked changes.

Reviewer 2
I like very much the introductory part, it is a very good compilation/resume of the state of the art of bone transformation research. I will suggest to try to resume all the information in a graph where the X axe is the burning temperature/time (qualitative and/or quantitative) and all the mentioned parameter-changes (Y axe) are depicted.

Thank you very much for your time and helpful feedback for our manuscript. It was a concern of ours that the diverse researchers who may be interested in this topic (ranging in disciplines from zooarchaeology to material scientists and thermochemists) may need a full background to provide a comprehensive understanding of the many complex systems being considered, so it was our original intention to have a very extensive background section. From the feedback of our other reviewers, however, it was suggested that this be shortened and made more concise. We believe the revisions have keep the scope and scale of the original intention for the background section while eliminating some more extraneous details and clarifying the language.

Additionally, we have tried to create several versions of a schematic following your suggestion. All versions were found to be unsatisfactory, both due to the changing scenarios around burning with or without oxygen, and the variance found in reported data regarding porosity which we do not directly investigate in our own study. Our solution was to clarify the language regarding the timing of the described mechanisms, primarily found in our revised discussion section is highlighted on page 22 of the manuscript with tracked changes.

First paragraph of page 13: Describe better the sedimentological/postdepositional features indicating the mentioned features/processes. Any images of the deposits and/or bone remains?

We thank you for your suggestion. We have described the sedimentological context to the extent of our current knowledge, and have revised our text regarding our intention for the archaeological material to be a reference sample into bioapatite crystallinity and organic components of archaeological burned bone. In our forthcoming study on the full zooarchaeological material from Tolbor-17, of which the hypotheses generated by this paper will be tested with further studies, we plan on including a greater amount of detail on the post-depositional taphonomic processes and the greater geological context of Unit 3.

Include/discuss more FTIR-ATR related references for archaeological studies in the discussion, please reivew this recent work and references therein: Iriarte et al., 2020

We are grateful for your encouragement to include the very relevant work of Iriarte et al., 2020. We have now included this work in our discussion of the potential of burnt bone to make advances in our understanding of past human behavior.

Reviewer 3
The abstract is not informative about the results but mostly talk about methods. I suggest to rewrite it.

Thank you for your suggestion, and for your constructive feedback. The abstract has been fully rewritten to address your suggestion.

Introduction: you explain the aims of your study, but I think that a major implication of your work is not considered in this paper. I suggest to discuss in the introduction section (and maybe in the discussion or conclusion) a further implication: are your results relevant in the field of radiocarbon dating on bones or

collagen and DNA extraction? I think that you may discuss the importance of changes in crystallinity of bone apatite in the context of radiocarbon dating of archaeological bones.

We thank you for observation, and we have included the discussion of C14 dating of the inorganic component of bone in our revised manuscript. We are grateful for the expansion of the implications and relevance of our study, and this has provided an additional opportunity for us to contextualize and highlight the massive bioapatite mineral structural reorganization of calcined bone. Importantly, it is the same mechanisms which are responsible for the usefulness of calcined bioapatite for C14 dating (thermodynamically stable crystals that can resist contamination) that we describe in this study first at temperatures of calcination (~700˚C), and also a second threshold at higher temperatures (~900˚C). This discussion and many relevant citations is included in our revised section on burnt bone diagenesis which is highlighted on page 10 our manuscript with tracked changes.

We believe our revised text and clarification on the timing of organic loss, and therefore waning usefulness for collagen and DNA extraction, will benefit those audiences as our results provide reference spectroscopic datasets illustrating the presence and quick decay of organic components in burnt bone.

The Background section is really too long. It seems a summary of the background chapters of a PhD dissertation. I suggest to strongly reduce this section and possibly to add some parts to supplementary material or add a new table…

Thank you. We had major concerns about the background necessary for all varied audiences who may be interested in this paper, and it was our original intention to have a detailed background to meet those needs. Considering your suggestion, we have completely rewritten and restructured our background sections. We believe this revised version still has the breadth to inform readers from different disciplines, but is no longer dense and focused on tangential information.

I noted that you completely missed the many papers published by Gregorio dal Sasso of the University of Padua (Italy). His PhD project was dedicated to the issue of archaeological bones diagenesis after burying and he investigated in details the behaviour of buried bones in terms of changes in crystallinity and interaction with water in the archaeological deposit. I strongly suggest especially to consider the paper elaborating an universal curve for apatite crystallinity (https://www.nature.com/articles/s41598-018-30642-z).

Thank you for your suggestion to reference the research of Gregorio dal Sasso, specifically dal Sasso et al. (2018). We are familiar with the work of dal Sasso and believe the universal curve of apatite crystallinity described in dal Sasso et al. (2018) is a significant recent advancement in the use of spectroscopy to evaluate the preservation of archaeological bone. We plan on using this methodology more extensively in our future work planned on the entire Tolbor-17 faunal assemblage, a comprehensive zooarchaeological study which is forthcoming.

I would also suggest to add (maybe in supplementary material) the section of Tolbon indicating the stratigraphic position of sampled bones. Please condor also that if bones come from different parts of the archaeological despots, they may have suffered different (I mean local) diagenetic process (e.g. related to water percolation or variations of pH values of the host archaeological layer); can this influence your results? the sole map of the study region is completely useless if you don't supply data on the stratigraphy of the site. Finally, it is not clear why you selected this specific archaeological sequence to carry out your experiments.

Thank you for your suggestion. As all burned faunal material sampled comes from a constrained area of one of the Tolbor-17 test pits and was considered to be firmly within the stratigraphic Unit 3, we do not believe this will be a complicating factor for our current study. We do plan on including more detailed sedimentological and geographic information in our more comprehensive zooarchaeological study of the full Tolbor-17 faunal assemblage, however, which will test our hypothesis of preservation bias highlighted by our conclusions further. We have clarified the stratigraphic position of the samples in our text to address this, however, and revised text can be found highlighted on page 13 of our manuscript with tracked changes.

We hope that we have also been able to clarify the inclusion of the Tolbor-17 samples in our study as initial investigations into the novel faunal preservation of Upper Paleolithic burned bone from the Tolbor valley, as well as representative samples of archaeological material sufficient to include as a reference sample to compare to our modern experimental samples. Text to acknowledge this in greater detail can be found highlighted on page 13 in our manuscript with tracked changes.

The description of methods is also very long and many informations are very basic. I suggest to shorten this part of the manuscript or move some parts to supplementary material.

We have taken your comment into serious consideration, and have tried to use more concise language to describe our methodologies. Ultimately much of the basic information have remained due to our interest in providing comprehensive detail to those reading our paper from diverse sub-disciplines who perhaps would like to recreate our study.

Unfortunately, you do not present and discuss results of SEM investigation. Scanning microscope, I guess, may inform about the modification of apatite crystals of your samples; for instance changes in crystal shape or orientation, or can explain the evolution of diagenesis recording the process of recrystallization (evidence of dissolution and recrystallisation are very evident under the SEM). As you cited SEM in the Methods section, I think you should have interesting data to show. The only SEM images are in Fig. 7, but they are not discussed. SEM data would increase also the quality of discussion.

Thank you. For this study it was our original intention that the SE microscopy images be used for visualization purposes only, and have only selected five bones typical of their burning category for SE microscopy imaging (three from our modern experimental collection, and two from our archaeological assemblage). These bone samples were selected for imaging prior to being powdered for spectroscopic analyses, and it is no longer possible to image a more comprehensive sample of the other included burned modern and archaeological bone due to the powdering which took place. To address your suggestion, we have moved the detailed information regarding the SE microscopy instrument methodology to Supplemental Information and have clarified that the inclusion of SE images in this manuscript are for generalized visualization purposes only. In addition, greater detail of morphological observations are now included in the figure caption to address the processes which are present in our five images. We are excited for the suggestion to include a greater SE microscopy component in our future studies considering the thermal alteration of burnt bone, in which we will image a more thorough and comprehensive sample of material will be considered to conduct analyses and descriptions as you suggested.
