# Supplementary material for: Characterization of structural changes in modern and archaeological burnt bone: Implications for differential preservation bias
Source: PLoS One. 2021 Jul 28;16(7):e0254529. doi: 10.1371/journal.pone.0254529 (PMC8318310; doi:10.1371/journal.pone.0254529)
Supplement: Attachment — Submitted filename: Response to Reviewers .pdf [file pone.0254529.s009.pdf]

**Response to Reviewers:**

**Editorial comment:**

*Based on my own independent evaluation, I agree with Reviewer 2 that the current Discussion could very easily be expanded... However, given the general audience of PLOS One, it is equally critical to highlight to broader significance and importance of results.*

Thank you for your constructive suggestions and encouragement to expand our Discussion. We agree that broadening our scope and contextualizing the inclusion of archaeological material results in a much stronger manuscript which speaks to a larger audience.

*And as this presents novel data that has immediate relevance towards the archaeological sample under analysis, it would seem to be a significant missed opportunity to take these results on burnt bone and contextualise them in light of a larger consideration as to what significance this result will have to site-based interpretations.*

Thank you. We have modified our Discussion portion of the manuscript to revisit the site of Tolbor-17 and expand into greater detail regarding the biogeography and relevance of studying fire in the Ikh-Tolborin-Gol, as well as highlighting the importance and relevance of the Tolbor-17 site to address larger questions of human technology.

**Reviewer 1**

*There are very minor changes suggested included in the pdf file attached. These refer to including a reference, be more precise in the sample size and delete a sentence from conclusions that is not really conclusion form the manuscript.*

Thank you for your feedback and observations. The inclusion of van Hoesel et al. (2019) is an excellent contribution to our manuscript expanding on the experimental work regarding properties of combusted bone and a complement to the cited work of Reidsma et al. (2016). To address the faunal summary table, we have added number counts for the piece plotted material. We agree that the addition of this detail provides a more traditional representation of the zooarchaeology, and the quantification of the burnt bone fragments from screens will be a component of our future work on the Tolbor-17 fauna.

Thank you, additionally, for your recommendation regarding the sentence addressing small bone fragments in our conclusion. We have adjusted our text to clarify the recovery of nearly all burnt bone fragments were from the screened material, and have moved the majority of that text to our Methodology section to avoid references in the Conclusion portion of our manuscript.

**Reviewer 2**

*…[The] discussion is just focused in comparing experimental results with results from archaeological data, it is OK, but which are the implications of having burnt bones in T17 ? Why it is important to have burned bones? How is archaeologically interpreted Unit 3?*

Thank you for your suggestion and useful perspective on expanding our discussion of the archaeology and the significance of fire presence at Tolbor-17. We have added a much larger section of our Discussion paragraphs to address this, including contextualizing Tolbor-17 within the Ikh-Tolberin-Gol, as well as the ultimate reasons we are interested in investigating Upper Paleolithic fire further. We have also

detailed further the rare opportunity Tolbor-17 presents to study questions of this nature, as it is rare to have fire and faunal preservation in the valley.

*In the same way, in the title "differential preservation bias" is mentioned, but it is not discussed in the text...which bias is detected in T17 collection? Why?*

Thank you. We have clarified our text to specify that the experimental work characterizing bioapatite growth and crystallinity (demonstrated in both modern and archaeological bone) highlights a vulnerability of charred bones that has yet to be described. Our conclusions therefore provide implications for biases in archaeological assemblages of burnt bone that may impact the visibility and study of fire presence and properties from a faunal perspective, and can be hypothesis-generating for future studies considering site specific burnt bone preservation.
