# Supplementary material for: Characterization of structural changes in modern and archaeological burnt bone: Implications for differential preservation bias
Source: PLoS One. 2021 Jul 28;16(7):e0254529. doi: 10.1371/journal.pone.0254529 (PMC8318310; doi:10.1371/journal.pone.0254529)
Supplement: Attachment — Submitted filename: Response to Reviewers.docx [file pone.0254529.s007.docx]

**Response to Reviewers:**

**Reviewer 1**

*…However, the originality of the paper needs to be more explicitly described, crystallinity changes are referred to burning but very little if any is referred to diagenetic influence in burnt archaeological specimens (which is the objective of the paper but finally not clearly discussed). A reorganization of the text and more clear statements and aspects to be discussed to demonstrate the goals of the paper is needed.*

Thank you for your comment and for all your helpful suggestions. The manuscript is greatly improved with your feedback. To highlight the originality of the paper we have rewritten major portions of the introduction and background sections, attempting to summarize concisely the exact goals of the study and inclusion of the modern and archaeological samples. We believe this will reframe and clarify the role of the Tolbor-17 material for this study, as it is our intention that the Tobor-17 archaeological burnt bone serve as a comparative reference sample of changes in bioapatite crystallinity sizes and organic content for this study. Future zooarchaeological studies planned for Tolbor-17 will consider the hypothesis of differential preservation closely with supporting faunal and geoarchaeological data. We have therefore modified our conclusions to reflect this restructuring, using our results from the study as hypothesis generating observations for further closer study, with the intention to follow up more substantially in the forthcoming full zooarchaeological study of Tolbor-17.

*Burning alone does not cause fragmentation, a subsequent movement or effort may produce fragmentation, but indication of which movement or effort acted after burning is not described. Further, fragmentation before and after burning can be caused by a large number of taphonomic agents (e.g. butchery, trampling, weathering, corrosion…).*

Thank you for your observation. This perspective and related information will be the major focus of the next study planned for this project: a full zooarchaeological study of the Tolbor-17 faunal material. This planned study will include traditional zooarchaeological analyses including identifiable specimens, fragmentation degree, surface modifications, and preservation. We seek to describe many anthropogenic behaviors present in the Tolbor-17 fauna in the future study, and will pay specific attention to burning to test the hypothesis of differential preservation suggested by our current manuscript. This zooarchaeological investigation has had initial data collection completed in December 2019, with further analyses planned with international travel restrictions are lifted.

*Definitively, the paper needs a better and more exhaustive description of the fossil assemblage: number of specimens, size of fossils, surface modifications, how many fossils are in each burning stage or which have also been affected by any other taphonomic agent both before and after burning should be described or included in a table.*

We believe that through the reframing and clarification of the purpose of the archaeological material (as a reference sample confirming the visibility in changes of bioapatite crystallinity sizes and organic preservation in an archaeological assemblage and relating described changes to zooarchaeological observational scales of burning intensity) will clarify the intention of the archaeological sample inclusion. A sample of text addressing this specifically is now included in the introduction and is highlighted in the manuscript with tracked changes on page 3. We also have reframed our discussion of the archaeological material in the context of hypothesis generation, and the descriptive fossil assemblage desired here will be the focus of the next paper engaging with the planned zooarchaeological study of the full Tolbor-17 fauna from Unit 3. Language addressing this has been rewritten and is highlighted in page 23 of the manuscript with tracked changes.

**Reviewer 2**

*I like very much the introductory part, it is a very good compilation/resume of the state of the art of bone transformation research. I will suggest to try to resume all the information in a graph where the X axe is the burning temperature/time (qualitative and/or quantitative) and all the mentioned parameter-changes (Y axe) are depicted.*

Thank you very much for your time and helpful feedback for our manuscript. It was a concern of ours that the diverse researchers who may be interested in this topic (ranging in disciplines from zooarchaeology to material scientists and thermochemists) may need a full background to provide a comprehensive understanding of the many complex systems being considered, so it was our original intention to have a very extensive background section. From the feedback of our other reviewers, however, it was suggested that this be shortened and made more concise. We believe the revisions have keep the scope and scale of the original intention for the background section while eliminating some more extraneous details and clarifying the language.

Additionally, we have tried to create several versions of a schematic following your suggestion. All versions were found to be unsatisfactory, both due to the changing scenarios around burning with or without oxygen, and the variance found in reported data regarding porosity which we do not directly investigate in our own study. Our solution was to clarify the language regarding the timing of the described mechanisms, primarily found in our revised discussion section is highlighted on page 22 of the manuscript with tracked changes.

*First paragraph of page 13: Describe better the sedimentological/postdepositional features indicating the mentioned features/processes. Any images of the deposits and/or bone remains?*

We thank you for your suggestion. We have described the sedimentological context to the extent of our current knowledge, and have revised our text regarding our intention for the archaeological material to be a reference sample into bioapatite crystallinity and organic components of archaeological burned bone. In our forthcoming study on the full zooarchaeological material from Tolbor-17, of which the hypotheses generated by this paper will be tested with further studies, we plan on including a greater amount of detail on the post-depositional taphonomic processes and the greater geological context of Unit 3.

*Include/discuss more FTIR-ATR related references for archaeological studies in the discussion, please reivew this recent work and references therein: Iriarte et al., 2020*

We are grateful for your encouragement to include the very relevant work of Iriarte et al., 2020. We have now included this work in our discussion of the potential of burnt bone to make advances in our understanding of past human behavior.

**Reviewer 3**

*The abstract is not informative about the results but mostly talk about methods. I suggest to rewrite it.*

Thank you for your suggestion, and for your constructive feedback. The abstract has been fully rewritten to address your suggestion.

*Introduction: you explain the aims of your study, but I think that a major implication of your work is not considered in this paper. I suggest to discuss in the introduction section (and maybe in the discussion or conclusion) a further implication: are your results relevant in the field of radiocarbon dating on bones or collagen and DNA extraction? I think that you may discuss the importance of changes in crystallinity of bone apatite in the context of radiocarbon dating of archaeological bones.*

We thank you for observation, and we have included the discussion of C14 dating of the inorganic component of bone in our revised manuscript. We are grateful for the expansion of the implications and relevance of our study, and this has provided an additional opportunity for us to contextualize and highlight the massive bioapatite mineral structural reorganization of calcined bone. Importantly, it is the same mechanisms which are responsible for the usefulness of calcined bioapatite for C14 dating (thermodynamically stable crystals that can resist contamination) that we describe in this study first at temperatures of calcination (~700˚C), and also a second threshold at higher temperatures (~900˚C). This discussion and many relevant citations is included in our revised section on burnt bone diagenesis which is highlighted on page 10 our manuscript with tracked changes.

We believe our revised text and clarification on the timing of organic loss, and therefore waning usefulness for collagen and DNA extraction, will benefit those audiences as our results provide reference spectroscopic datasets illustrating the presence and quick decay of organic components in burnt bone.

*The Background section is really too long. It seems a summary of the background chapters of a PhD dissertation. I suggest to strongly reduce this section and possibly to add some parts to supplementary material or add a new table…*

Thank you. We had major concerns about the background necessary for all varied audiences who may be interested in this paper, and it was our original intention to have a detailed background to meet those needs. Considering your suggestion, we have completely rewritten and restructured our background sections. We believe this revised version still has the breadth to inform readers from different disciplines, but is no longer dense and focused on tangential information.

*I noted that you completely missed the many papers published by Gregorio dal Sasso of the University of Padua (Italy). His PhD project was dedicated to the issue of archaeological bones diagenesis after burying and he investigated in details the behaviour of buried bones in terms of changes in crystallinity and interaction with water in the archaeological deposit. I strongly suggest especially to consider the paper elaborating an universal curve for apatite crystallinity (*[*https://www.nature.com/articles/s41598-018-30642-z*](https://www.nature.com/articles/s41598-018-30642-z)*).*

Thank you for your suggestion to reference the research of Gregorio dal Sasso, specifically dal Sasso et al. (2018). We are familiar with the work of dal Sasso and believe the universal curve of apatite crystallinity described in dal Sasso et al. (2018) is a significant recent advancement in the use of spectroscopy to evaluate the preservation of archaeological bone. We plan on using this methodology more extensively in our future work planned on the entire Tolbor-17 faunal assemblage, a comprehensive zooarchaeological study which is forthcoming.

*I would also suggest to add (maybe in supplementary material) the section of Tolbon indicating the stratigraphic position of sampled bones. Please condor also that if bones come from different parts of the archaeological despots, they may have suffered different (I mean local) diagenetic process (e.g. related to water percolation or variations of pH values of the host archaeological layer); can this influence your results? the sole map of the study region is completely useless if you don't supply data on the stratigraphy of the site. Finally, it is not clear why you selected this specific archaeological sequence to carry out your experiments.*

Thank you for your suggestion. As all burned faunal material sampled comes from a constrained area of one of the Tolbor-17 test pits and was considered to be firmly within the stratigraphic Unit 3, we do not believe this will be a complicating factor for our current study. We do plan on including more detailed sedimentological and geographic information in our more comprehensive zooarchaeological study of the full Tolbor-17 faunal assemblage, however, which will test our hypothesis of preservation bias highlighted by our conclusions further. We have clarified the stratigraphic position of the samples in our text to address this, however, and revised text can be found highlighted on page 13 of our manuscript with tracked changes.

We hope that we have also been able to clarify the inclusion of the Tolbor-17 samples in our study as initial investigations into the novel faunal preservation of Upper Paleolithic burned bone from the Tolbor valley, as well as representative samples of archaeological material sufficient to include as a reference sample to compare to our modern experimental samples. Text to acknowledge this in greater detail can be found highlighted on page 13 in our manuscript with tracked changes.

*The description of methods is also very long and many informations are very basic. I suggest to shorten this part of the manuscript or move some parts to supplementary material.*

We have taken your comment into serious consideration, and have tried to use more concise language to describe our methodologies. Ultimately much of the basic information have remained due to our interest in providing comprehensive detail to those reading our paper from diverse sub-disciplines who perhaps would like to recreate our study.

*Unfortunately, you do not present and discuss results of SEM investigation. Scanning microscope, I guess, may inform about the modification of apatite crystals of your samples; for instance changes in crystal shape or orientation, or can explain the evolution of diagenesis recording the process of recrystallization (evidence of dissolution and recrystallisation are very evident under the SEM). As you cited SEM in the Methods section, I think you should have interesting data to show. The only SEM images are in Fig. 7, but they are not discussed. SEM data would increase also the quality of discussion.*

Thank you. For this study it was our original intention that the SE microscopy images be used for visualization purposes only, and have only selected five bones typical of their burning category for SE microscopy imaging (three from our modern experimental collection, and two from our archaeological assemblage). These bone samples were selected for imaging prior to being powdered for spectroscopic analyses, and it is no longer possible to image a more comprehensive sample of the other included burned modern and archaeological bone due to the powdering which took place. To address your suggestion, we have moved the detailed information regarding the SE microscopy instrument methodology to Supplemental Information and have clarified that the inclusion of SE images in this manuscript are for generalized visualization purposes only. In addition, greater detail of morphological observations are now included in the figure caption to address the processes which are present in our five images. We are excited for the suggestion to include a greater SE microscopy component in our future studies considering the thermal alteration of burnt bone, in which we will image a more thorough and comprehensive sample of material will be considered to conduct analyses and descriptions as you suggested.
